# Influenza-Induced Interferon Lambda Response Is Associated With Longer Time to Delivery Among Pregnant Kenyan Women

**DOI:** 10.3389/fimmu.2020.00452

**Published:** 2020-03-17

**Authors:** Christof Seiler, Nicholas L. Bayless, Rosemary Vergara, Jillian Pintye, John Kinuthia, Lusi Osborn, Daniel Matemo, Barbra A. Richardson, Grace John-Stewart, Susan Holmes, Catherine A. Blish

**Affiliations:** ^1^Department of Statistics, Stanford University, Stanford, CA, United States; ^2^Department of Data Science and Knowledge Engineering, Maastricht University, Maastricht, Netherlands; ^3^Immunology Program, Stanford University School of Medicine, Stanford, CA, United States; ^4^Department of Medicine, Stanford University School of Medicine, Stanford, CA, United States; ^5^Department of Global Health, University of Washington School of Medicine, Seattle, WA, United States; ^6^Kenyatta National Hospital, Nairobi, Kenya; ^7^Department of Biostatistics, University of Washington, Seattle, WA, United States; ^8^Chan Zuckerberg Biohub, San Francisco, CA, United States

**Keywords:** pregnancy, preterm, time to delivery, interferon, immune, influenza virus

## Abstract

Specific causes of preterm birth remain unclear. Several recent studies have suggested that immune changes during pregnancy are associated with the timing of delivery, yet few studies have been performed in low-income country settings where the rates of preterm birth are the highest. We conducted a retrospective nested case-control evaluation within a longitudinal study among HIV-uninfected pregnant Kenyan women. To characterize immune function in these women, we evaluated unstimulated and stimulated peripheral blood mononuclear cells *in vitro* with the A/California/2009 strain of influenza to understand the influenza-induced immune response. We then evaluated transcript expression profiles using the Affymetrix Human GeneChip Transcriptome Array 2.0. Transcriptional profiles of sufficient quality for analysis were obtained from 54 women; 19 of these women delivered <34 weeks and were defined as preterm cases and 35 controls delivered >37 weeks. The median time to birth from sample collection was 13 weeks. No transcripts were significantly associated with preterm birth in a case-control study of matched term and preterm birth (n = 42 women). In the influenza-stimulated samples, expression of IFNL1 was associated with longer time to delivery—the amount of time between sample collection and delivery (n = 54 women). A qPCR analysis confirmed that influenza-induced IFNL expression was associated with longer time to delivery. These data indicate that during pregnancy, *ex vivo* influenza stimulation results in altered transcriptional response and is associated with time to delivery in cohort of women residing in an area with high preterm birth prevalence.

## Introduction

Preterm birth, defined as birth before the 37th week of gestation, is a highly prevalent disorder of pregnancy that is a major driver of childhood morbidity and mortality (1–4). Preterm birth is divided into categories based on timing: extremely preterm (before 28 weeks), very preterm (28–32 weeks), and moderate to late preterm (32–37 weeks). These categories may differ in their pathogenic underpinnings, which can include genetic factors, multiple pregnancies, infections, and chronic conditions such as diabetes and high blood pressure (5, 6). While most research on the risk factors associated with preterm birth has occurred in higher-income countries, lower-income countries have higher rates of preterm births than high-income countries (12 vs. 9%), with more than 60% of preterm deliveries occurring in Africa and South Asia (7). An improved ability to predict who is at risk of preterm birth across different settings is of high public health importance.

Increasingly, attention has turned to the critical role the immune system plays in establishing and maintaining a healthy pregnancy (8). Changes in immune function during pregnancy may increase susceptibility to, or complications from, a wide range of infectious diseases (9). Further, pregnancy is not a globally immunosuppressed state; some cell types increase in frequency and function while others have diminished function, both locally and systemically, during pregnancy (8, 10–16). Thus, it is possible that immune mechanisms play a significant role in driving preterm birth, an idea supported by the fact that many of the known risk factors for preterm birth also alter immune function. Consistent with this idea, several studies have demonstrated that preterm birth is associated with altered inflammatory responses, particularly detectable in the placenta and in the amniotic fluid (5). For instance, elevated expression of the inflammatory cytokine tumor necrosis factor (TNF) in the amniotic fluid was associated with both infection and preterm birth (17). A recent study demonstrated that activated T cells are found at the maternal-fetal interface in women with spontaneous preterm delivery, and that, in a mouse model, treatment with progesterone can attenuate this inflammation and reduce risk of preterm birth (18).

Transcriptomic evaluation of the uterus and fundus during labor in term and preterm birth have identified inflammatory signatures associated with labor and preterm birth (19–22). Several additional studies have identified transcriptional signatures of inflammation in the placentas of women who undergo preterm delivery (23, 24). While it is clear that local inflammation is associated with preterm birth, several additional studies have sought to identify such signatures in the blood, as this will be a more appropriate biomarker to stage interventions in the long run. One group identified a blood transcriptomic inflammatory signature associated with preterm delivery, but as the women were in labor this could have been a signature of labor itself and not the underlying cause of preterm birth (25). This is particularly true as labor itself is associated with a significant inflammatory signature (26–28). Heng et al. aimed to address this concern, investigating maternal whole blood gene expression profiles associated with spontaneous preterm birth in asymptomatic pregnant women (29). In this study of 51 spontaneous preterm births and 114 term deliveries in Calgary, they determined significant clinical factors and differential gene expression profiles of inflammation including leukocyte migration, lysosomes, NK-kB activation, and pathways involving cytokines and their receptors (including IFN) were associated with spontaneous preterm birth (29). Consistent with the idea that preterm birth is an inflammatory state, monocytes from women with a history of preterm birth demonstrated enhanced inflammatory responses in response to stimulation *in vitro* (30). More recently, significant progress was made in identifying signatures predictive of preterm birth and delivery timing using cell-free RNA in the blood (31, 32). The studies performed to date have varied in the specific gene signatures identified, and in fact, there has been very poor agreement between studies in the genes identified (33). Despite significant heterogeneity in the specific genes identified, a consistent theme has been that an inflammatory signature is associated with preterm birth. One limitation of these studies is that all have been performed in North American women, and it is not clear if the signatures identified are generalizable to other populations.

In addition to finding genes associated with the risk of preterm birth, another goal has been to identify the factors that control the timing and onset of labor (34). One proposal is that the “clock” for pregnancy is controlled either through the placenta (35, 36) or the decidua (37). Building on this idea, one recent study sought to gain insight into associations between immune function and the timing of delivery using mass cytometry to profile a wide range of immune cells and their function throughout pregnancy in 18 healthy women who delivered at term (38). Their analysis revealed a large number of synchronous changes in various cellular lineages during pregnancy. These changes included increased STAT1 signaling responses following stimulation with interferon (IFN) alpha and beta in multiple innate and adaptive immune cell types and increased STAT5 activity among multiple T cell subsets (38). Overall, their data supported the idea that changes in cellular programs, not a single cell type, were predictive of the timing of pregnancy, leading to the idea that an “immune clock” could regulate pregnancy (38). Overall, these studies have indicated that assessing immune function may allow us to estimate the timing of delivery and the risk of preterm birth.

Here we set out to identify associations between immune function and pregnancy outcomes (preterm birth and the timing to delivery) in a cohort of women in Kisumu, Kenya, an area with a high prevalence of preterm birth. We profiled immune function using RNA microarrays in peripheral blood mononuclear cell (PBMC) samples from pregnant women who were followed until birth. In order to identify perturbations in the ability of immune cells to respond to threats, PBMC samples were tested directly *ex vivo* and also following an *in vitro* stimulation by viral infection.

## Materials and Methods

### Study Design

The objective of this study was to identify associations between immune function and pregnancy outcomes (preterm birth and the time to delivery) in a setting of high prevalence of preterm birth. We used previously banked samples from a prospective cohort study of HIV-1 acquisition in pregnant and postpartum Kenyan women, the *Mama Salama Study* (39). The *Mama Salama Study* enrolled 1304 women during pregnancy. Gestational age was estimated based on last menstrual period. Ultrasound was used to confirm gestational age when there was a difference in estimated age of more than 2 weeks between fundal height estimate and last menstrual period estimate. Participants were eligible for inclusion in this substudy if they had a PBMC sample collected at their enrollment visit during pregnancy with 2 aliquots available. Twenty-five potential cases were defined as ≤34 weeks gestation at birth, singleton birth, uninfected with HIV at enrollment and follow up, live birth, vaginal or unplanned cesarean delivery, and known delivery date. Fifty potential controls were defined as ≥37 weeks gestation at birth and with all other criteria of cases. Two analyses were performed: first a case control study comparing the women who gave birth preterm to the controls, and second a study evaluating associations between transcript levels and the time to delivery in all the women analyzed. All subjects gave informed and written consent. All studies were performed in accordance with the Declaration of Helsinki, and all study procedures were approved by the Institutional Review Boards of the University of Washington, Seattle, WA, USA (UW IRB #38472) and Kenyatta National Hospital, Nairobi, Kenya (KNH IRB P114/4/2010).

### Sample Procesesing and Microarrays

PBMCs were purified in Kisumu, Kenya by Ficoll density gradient centrifugation and stored in liquid nitrogen before shipping to the University of Washington and subsequently to Stanford University. PBMCs were thawed rapidly in a 37°C water bath before dropwise addition of 1 mL of warm complete RPMI media (supplemented with 10% FBS and antibiotics) containing Benzonase (nuclease added to reduce cell clumping). The sample was transferred to a 50 ml conical tube containing an additional 9 mL of pre-warmed media with Benzonase. Cells were spun for 10 min at 400 × g, the supernatant was decanted, and the cell pellet was resuspended in 10 mL complete RPMI before cell counting using a BioRad TC20 cell counter. Cells were spun again for 10 min at 400 × g, the supernatant was removed, the cells were resuspended in RPMI media and were dispensed into a 96-well U bottom plate at 10^6^ cells/well. To each well, either PBS or influenza virus (H1N1/California/2009) was added at an MOI of 1 for 1 h, followed by the addition of serum-containing media for 6 additional hours at 37°C. Following incubation, cells were centrifuged, washed once with complete RPMI (200 uL/well), then resuspended in RNA lysis buffer (Qiagen). RNA was extracted from each well using the column-based RNeasy kit (Qiagen) according to the manufacturer's instructions. Purified RNA was quantified and characterized using a Nanodrop 3000. For samples with RNA Integrity Number > 6, RNA aliquots were submitted for microarray analysis (Human Transcriptome Array, Affymetrix) at the Stanford PAN Facility (http://pan.stanford.edu/index.html).

### Preprocessing of Microarray Data

We normalized the raw expressions using the Robust Multichip Average (RMA) algorithm implemented in R package *oligo* (40). We removed background probes and probes that could not be mapped to known gene symbols. We then filtered the remaining probes by thresholding the mean of normalized counts to maximize the number of differentially detected probes at a fixed False Discovery Rate (FDR). We used automatic independent filtering as described in the *DESeq2* package (41). Finally, we explored the preprocessed expressions with Principal Component Analysis.

### Statistical Modeling of Microarray Data

We performed differential expression analysis using an empirical Bayes approach implemented in R package *limma* (42). We added biological context using the R package *BioNet* by assigning unadjusted *p*-values to nodes of a network (43, 44). *BioNet* derives the gene network structure form the protein-protein interaction database STRING (45). This allowed us to assemble differentially expressed gene networks controlled at a fixed FDR. We also performed pathway analysis by calculating the overlap between our discovered network with known pathways in KEGG (46) using R package *KEGGREST* (47). We calculated the overlap as the number of intersecting genes in a pathway and our network, divided by the total number of genes in a pathway. In all our models, we use gene expression as the response variable. The case-control analysis is an analysis on paired samples where each preterm and term match are a pair. We used *R* package *matchit* and the method “optimal.” Optimality is defined as the smallest average absolute distance across the matched pairs (48). The model is encoded in *limma* using the following design formula: gene expression ~ pair + preterm indicator. For the time to delivery analysis, we coded the model in *limma* as follows: gene expression ~ time to delivery + gestage age at delivery. For both analyses, we subsetted data to stimulated and unstimulated samples before fitting separate models to each subset. Initially, our analysis used interaction terms on the combined data which was harder to interpret, but yielded similar results.

### Real-Time PCR Analysis

Complementary DNA (cDNA) was generated using the SuperScript VILO Master Mix Kit (Thermo Fisher 11755250) using the total RNA isolated from each PBMC sample. Quantitative polymerase chain reaction (qPCR) was performed using the Taqman Universal Master Mix II Kit with UNG (Thermo Fisher 4440044). Primers and probes were ordered as separate oligos through ElimBio (Hayward, CA) with added modification 5 -FAM/3 -BHQ-1 to the probes: IFNL Forward Primer 5′-ATC TGT CAC CTT CAA CCT CTT C-3′, IFNL reverse primer 5′-GTA GGG CTC AGC GCA TAA ATA-3′, IFNL Prober 5′-AAT ATG TGG CCG ATG GGA ACC TGT- 3′, Samples were run on the Thermo Fisher Scientific Step One Real-Time PCR System. All values were standardized to the constitutively expressed hypoxanthine phosphoribosyltransferase 1 (HPRT1) endogenous control (Thermo Fisher 4326321E) and relative mRNA expression levels were determined using the 2-ΔΔCT analysis method.

### Statistical Modeling of Real-Time PCR Data

We fitted a robust linear model using an M-estimator with the Huber weight function, implemented in R package *MASS* (49). We fit one model to unstimulated samples, and a second model to stimulated samples. The models have the following terms: gene expression ~ time to delivery + gestational age at birth. We computed confidence intervals and *p*-values using bootstrap resampling (50) with R packages *car* (51) and *boot* (52, 53).

## Results

### Case-Control Analysis With Gestational Age Matching

To assess whether there were significant changes in expression patterns of transcripts between the women who gave birth preterm vs. those who gave birth at term in an area of high prevalence of preterm birth, we used PBMCs from 42 women enrolled in the Mama Salama study in Kisumu, Kenya in whom we obtained sufficient quality RNA. As there was variation in the gestational age at which PBMCs were collected, we matched preterm and term women on gestational age at sample collection. The matching results are illustrated with a dot plot in Supplementary Figure 1A (for stimulated samples) and Supplementary Figure 1B (for unstimulated samples). The demographics of these individuals are summarized in Table 1. The twenty cases gave birth at median gestational age of 30.2 weeks, which was significantly less than in the controls, which had a median gestational age of 39.2 weeks (Table 1). The median time to delivery (time from sample collection to delivery) also differed significantly, at 6.3 weeks for cases (preterm) and 19 weeks for controls (term). Cases and controls did not significantly differ in terms of the age of the women or other risk factors for preterm birth such as number of children, years since last birth, and STIs (Table 1)

**Table 1:** Characteristics of the study population for case control analysis, by case status^a^ (*n* = 42).

	**N (Percentage) or Median (Interquartile range)**				
**Characteristic**	***N***	**All women** **(*n* = 42)**	**Controls** **(*n* = 22)**	**Cases** **(*n* = 20)**	***p*-value^**e**^**
**Demographic**					
Age (years)	42	22.0 (20.0, 24.0)	22.5 (19.0, 24.0)	22.0 (20.0, 26.0)	0.69
Highest level of education (years)	42	8.0 (8.0, 12.0)	8.0 (8.0, 12.0)	8.0 (7.5, 8.5)	0.23
<8 years of completed education	42	27 (64%)	12 (55%)	15 (75%)	0.17
Currently married	42	34 (81%)	19 (86%)	15 (75%)	0.35
Duration of partnership (years)^b^	37	3.0 (1.0, 6.0)	2.5 (0.5, 4.5)	4.0 (2.0, 6.0)	0.10
Partner >10 years older^b^	34	6 (18%)	4 (24%)	2 (12%)	0.37
Employed	42	22 (52%)	13 (59%)	9 (45%)	0.36
Crowded living conditions (>3 people/room)	42	11 (26%)	6 (27%)	5 (25%)	0.87
**Sexual behavior and partner characteristics**					
Number of sexual acts (last 30 days)	42	2.0 (0.0, 4.0)	2.5 (1.0, 4.0)	1.0 (0.0, 2.5)	0.12
Any reported condomless sex (last 30 days)	42	28 (67%)	16 (73%)	12 (60%)	0.38
Number of sexual partners (last 30 days)	42	1.0 (1.0, 1.0)	1.0 (1.0, 1.0)	1.0 (1.0, 1.0)	0.55
Circumcised male partner^b^^,^ ^c^	38	11 (29%)	6 (30%)	5 (28%)	0.88
HIV-infected partner^b^^,^ ^c^	30	2.0 (0.0, 4.0)	2.5 (1.0, 4.0)	1.0 (0.0, 2.5)	0.12
**Gynecological history**					
Gestational age at enrolment	42	21.0 (19.0, 24.0)	21.0 (19.0, 23.0)	21.0 (19.0, 24.0)	0.89
Gestational age at birth	42	37.4 (30.9, 39.3)	39.2 (38.3, 41.0)	30.2 (27.0, 32.4)	<0.001^*^
Number of children	42	1.0 (1.0, 2.0)	1.0 (0.0, 2.0)	1.5 (1.0, 2.5)	0.48
<2 years since last birth^d^	27	3 (11%)	1 (8%)	2 (13%)	0.68
Any reported vaginal washing (last week)	42	16 (38%)	8 (36%)	8 (40%)	0.81
Any reported vaginal drying (last week)	42	4 (10%)	1 (5%)	3 (15%)	0.25
Self-reported history of STIs	42	2 (5%)	2 (9%)	0 (0%)	0.17
**Laboratory-confirmed STI diagnosis**					
*Trichomonas vaginalis*	42	2 (5%)	0 (0%)	2 (10%)	0.13
*Chlamydia trachomatis*	42	1 (2%)	1 (5%)	0 (0%)	0.33
*Neisseria gonorrhoeae*	42	1 (2%)	0 (0%)	1 (5%)	0.29
Syphilis	30	0 (0%)	0 (0%)	0 (0%)	-
Bacterial vaginosis	42	14 (33%)	8 (36%)	6 (30%)	0.66
Candidiasis	42	8 (19%)	2 (9%)	6 (30%)	0.12

*From the total of 42 women (some of which had only either stimulated or unstimulated samples available), we 1:1 matched 19 cases/controls in stimulated samples, and 20 cases/controls in unstimulated samples*.

* *p <0.05*.

a *Missing data not shown; all characteristics assessed at baseline unless indicated*.

b *Among women reporting current relationship*.

c *Male circumcision and HIV status of male partners reported by female partner*.

d *Among women with >1 children*.

e *Kruskall-Wallis tests for continuous measures and Chi-squared tests for proportions detected differences in baseline characteristics between preterm birth cases and controls. Fisher's exact tests were used for cell counts <10*.

**Figure 1:**
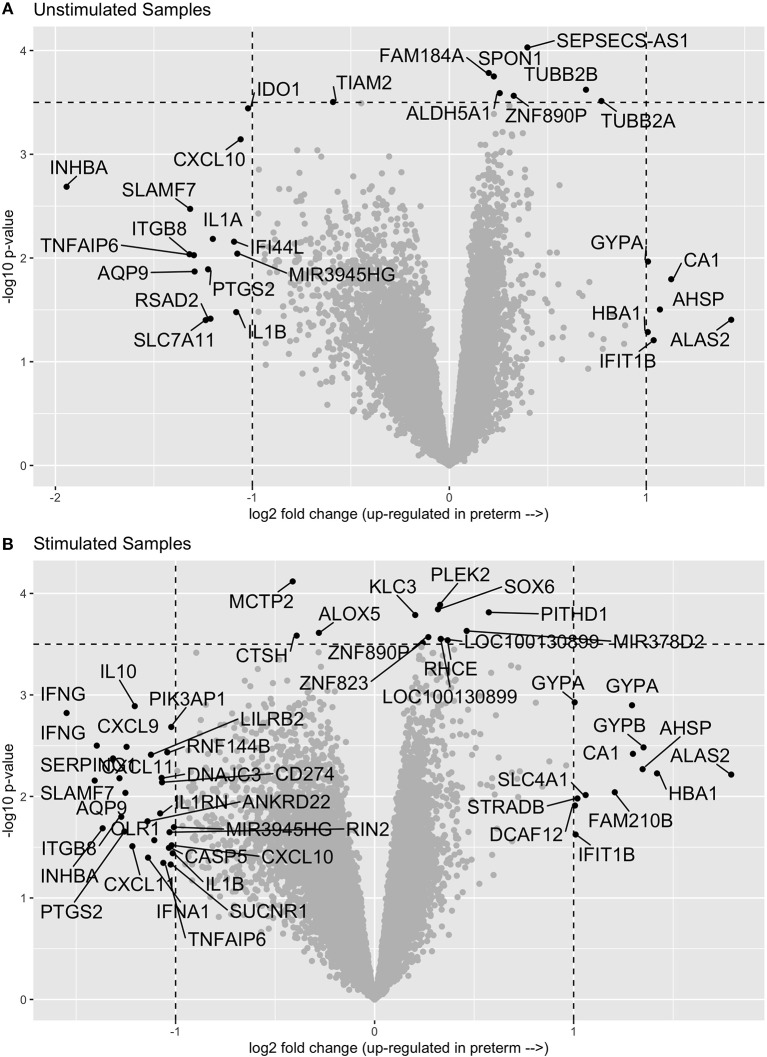
Volcano plots of gene expression comparisons between women who have birth preterm vs. at term in unstimulated **(A)** and influenza-virus stimulated **(B)** PBMC samples. No genes were significantly associated with preterm birth after correcting for multiple comparisons.

In comparing the transcriptome profiles between cases and controls, we chose to stimulate the samples in order to increase the chances of uncovering differences in immune function, which are best observed following stimulation. Thus, we evaluated gene expression in both unstimulated PBMCs and in PMBCs stimulated *in vitro* with the human influenza virus as previously described (10). We then compared the expression profiles of cases and controls in both the unstimulated and influenza-stimulated samples. After adjusting for multiple comparisons with an FDR of 0.1, we did not identify any genes that were significantly differentially expressed between cases and controls (Figure 1A and Supplementary Material). Similarly, no genes were significantly differentially expressed between preterm birth cases and controls in the influenza-stimulated samples (Figure 1B and Supplementary Material).

### Time to Delivery Analysis

In our secondary analysis, we evaluated samples from all 54 women with samples that yielded high-enough quality RNA in both stimulated and unstimulated samples. The demographics of these women are described in Table 2. We fitted a linear model to evaluate the associations between transcript levels and the time to delivery, controlling for gestational age at birth. The time to delivery was defined as the time interval between when the sample was collected and the actual birth.

**Table 2:** Characteristics of the study population for time to delivery analysis, by case status^a^ (*n* = 54).

	***N*** **(Percentage) or Median (Interquartile range)**	
**Characteristic**	***N***	**All women** **(*n* = 54)**
**Demographic**		
Age (years)	54	22.0 (19.0, 24.0)
Highest level of education (years)	54	8.0 (7.0, 12.0)
≤8 years of completed education	54	35 (65%)
Currently married	54	42 (78%)
Duration of partnership (years)^b^	47	3.0 (1.0, 6.0)
Partner ≥10 years older^b^	43	12 (28%)
Employed	54	25 (46%)
Crowded living conditions (≥3 people/room)	54	17 (31%)
**Sexual behavior and partner characteristics**		
Number of sexual acts (last 30 days)	54	1.0 (0.0, 3.0)
Any reported condomless sex (last 30 days)	54	35 (65%)
Number of sexual partners (last 30 days)	54	1.0 (1.0, 1.0)
Circumcised male partner^b^^,^ ^c^	49	14 (29%)
HIV-infected partner^b^^,^ ^c^	39	1 (3%)
**Gynecological history**		
Gestational age at enrolment	54	21.0 (19.0, 24.0)
Gestational age at birth	54	39.0 (31.7, 40.9)
Number of children	54	2.0 (1.0, 3.0)
<2 years since last birth^d^	34	4 (12%)
Any reported vaginal washing (last week)	54	23 (43%)
Any reported vaginal drying (last week)	54	6 (11%)
Self-reported history of STIs	54	3 (6%)
**Laboratory-confirmed STI diagnosis**		
*Trichomonas vaginalis*	54	2 (4%)
*Chlamydia trachomatis*	54	2 (4%)
*Neisseria gonorrhoeae*	54	1 (2%)
Syphilis	36	0 (0%)
Bacterial vaginosis	54	17 (31%)
Candidiasis	54	13 (24%)

a *Missing data not shown; all characteristics assessed at baseline unless indicated*.

b *Among women reporting current relationship*.

c *Male circumcision and HIV status of male partners reported by female partner*.

d *Among women with ≥1 children*.

**Figure 2:**
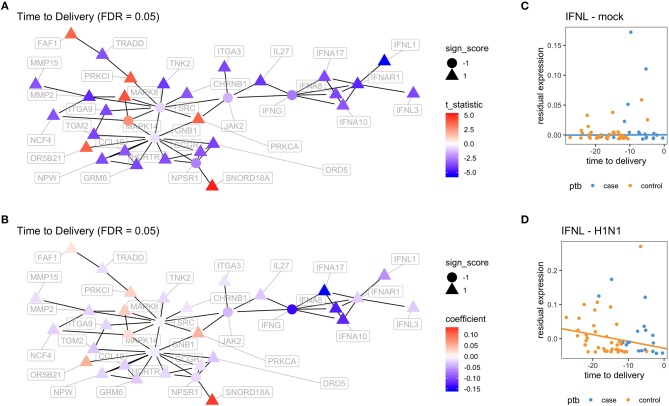
Virus-induced induction of interferon is associated with the time to delivery. **(A)** Network analysis with *p*-values from fitting a model that explains gene expression from time to delivery with gestational age at birth as a covariate. The shape of the nodes represent how these nodes contribute to the overall score of the network. Triangles contribute positively and increase the overall network false discovery rate (FDR), whereas circles contribute negatively and decrease overall FDR. The color gradient represents *t*-statistics. **(A)** positive *t*-statistic means that gene expression increases with approaching delivery. **(B)** Network analysis as in **(A)** with the color gradient representing the actual estimated regression coefficients. Confirmatory RT-PCR analysis of IFN-lamba expression was performed on unstimulated PBMCs **(C)** and on influenza-stimulated PBMCs **(D)**. Expression levels were fit to a linear model with the following covariates: intercept, time to delivery, and gestational age at birth. Shown are fitted time to delivery slopes for unstimulated mock **(C)** and stimulated H1N1 **(D)** samples. The slope on the H1N1 stimulated samples is significant (*p* = 0.002). The *y*-axis is the residual expression not explained by gestational age at birth.

In order to better visualize the biological significance of these findings, we fitted the same linear model individually on the unstimulated and stimulated samples. In our analysis on unstimulated samples, we found no genes whose expression levels could be explained by the time to delivery variable (TimeToDelivery_Microarray_Mock.pdf). In the analysis of samples stimulated with influenza virus, we detected 170 differentially expressed genes (TimeToDelivery_Microarray_H1N1.pdf, Supplementary Table 1) at an FDR of 0.1, and 6 genes at an FDR of 0.05 (Table 3). The top hit was IFNL1. To visualize the genes whose expression levels following influenza-virus stimulation were associated with time to delivery, we mapped these transcripts to known protein-protein interactions using the STRING database as previously described (54, 55). We colored coded this network analysis result using *t*-statistics (Figure 2A) and regression coefficients (Figure 2B). Gene nodes colored in red increase expression as women get closer to delivery, whereas blue nodes decrease expression. This analysis reveals networks of genes, particularly within the IFN pathway, that decrease in their expression in response to influenza stimulation as women get closer to delivery. A further network analysis shows that 8% of genes overlap with the Rig I Pathway (Supplementary Figure 2).

**Table 3:** Differentially expressed genes in stimulated samples for time to deliver term.

**Top 6 genes ordered by** ***p*****-value for time to delivery term**.			
**Gene symbol**	**Coefficient**	**Unadjusted *p*-value**	**Adjusted *p*-value**
IFNL1	−0.06	1e-07	0.018
NEURL1B	−0.03	1e-06	0.020
INPP5A	0.03	1e-06	0.020
SNORD18A	0.13	1e-06	0.024
KRT33A	−0.02	1e-05	0.049
PAQR4	−0.03	1e-05	0.049

*We included the following covariates in the linear model: intercept, time to delivery, and gestational age at birth*.

*In the table, we show the top 6 genes for the time to delivery term. See Figure 2 for a full network analysis on all genes. The full table is in the Supplementary Material*.

To confirm the association between influenza-induced IFN stimulation and the time to delivery, IFN lambda transcript levels were assessed by qPCR (TimeToDelivery_qPCR_H1N1.pdf). We fitted a robust linear regression model. The robust fit yielded a *p*-value of 0.002 for the time to delivery slope in the stimulated samples. Figures 2C,D shows the two estimated slopes from the robust regression fit. The fit suggests that, in stimulated samples, the expression of IFNL decreases as women approach delivery. This result further confirms our observations from the microarray data.

## Reproducibility

The entire analysis workflow is written in R markdown and available from our GitHub repository at https://github.com/ChristofSeiler/PTB_Study. The resulting reports can be found in the Supplementary Material. To rerun and reproduce all plots and results knit the corresponding Rmd files (e.g., in RStudio using Knit button). Before knitting make sure to copy all. CEL files into the same folder as the Rmd file.

## Discussion

Preterm birth is a major contributor to childhood morbidity and mortality worldwide, particularly in low income countries. Better understanding of the mechanisms underlying preterm birth may highlight pathways that could be therapeutically targeted to reduce the rates of preterm birth. One of the major challenges in identifying such mechanisms is the fact that several distinct pathways, including prior pregnancy history, infection, and genetic factors can contribute to preterm birth (2, 56). Many of these risk factors can significantly influence immune function, which may therefore act as a biomarker for preterm birth, highlighting the importance of understanding the immune system during pregnancy (57). Thus, here we set out to identify immune pathways associated with preterm birth and the timing of delivery in a cohort of women in Africa, an area where determinants of preterm birth are understudied.

Our case control analyses did not reveal any transcripts that were significantly associated with preterm birth, but we hope the availability of this data will inform future studies. We did find that the magnitude of the influenza-induced IFN-lambda transcriptional response significantly decreased as delivery approaches, suggesting that such inflammatory pathways are influenced temporarily during pregnancy. Overall, these data highlight the heterogeneity in these pathways and the difficulty in identifying a single factor predictive of preterm birth. In fact, a prior meta-analysis highlighted the heterogeneity in preterm birth (33). This study found minimal overlap in the genes associated with preterm birth across a range of studies, despite the fact that most were performed in North America. Given that prior studies have not found definitive gene signatures for preterm birth, we are not surprised that a more clear signature did not emerge in this novel study from sub-Saharan Africa.

Many aspects of immune function are altered during pregnancy to strike a balance between accommodating the semi-allogeneic fetus and protecting both mother and fetus from infection (9). These changes alter immunity to a wide range of pathogens, including influenza virus, which significantly drives morbidity and mortality in the pregnant population. In general, women have enhanced innate NK cell, monocyte, and dendritic cell responses to influenza virus (10, 11, 13, 58), yet there are also reports that there is reduced IFN-alpha and IFN-lambda production in response to influenza (59). In general, adaptive immune function is thought to be suppressed during pregnancy, though pregnant women respond adequately to influenza immunization (9, 60). Here we find that the IFN-lambda transcriptional response to influenza infection is blunted as delivery approaches, suggesting a less robust antiviral response. In fact, our network analysis demonstrated that not just IFN-lambda, but a variety of IFN genes and transcripts associated with the RIG-I pathway were reduced later in pregnancy. This could represent an adaptation necessary to tolerate the increasing burden of the semi-allogeneic fetus. This is consistent with the idea that baseline inflammation can blunt responses to stimuli (61). In fact, this could be entirely consistent with the inflammatory signatures identified in earlier studies (5, 17–24), all of which were performed in the absence of an exogenous stimulus.

Our finding that an entire transcriptional network of IFN-associated genes was temporally altered in pregnancy was consistent with the study by Aghaeepour and colleagues, in which they found multiple functional modules that were temporally altered during pregnancy, leading them to propose the idea of an “immune clock” (38). Both studies show temporal regulation of immune function during pregnancy despite a range of difference in approach, including evaluation of transcriptome vs. proteome, stimulation with virus vs. cytokine/chemical activation, inclusion of women who gave birth preterm and at term vs. full term only, and setting in Kenya vs. North America. Thus, while the pathways identified are different, this is not surprising as the prior study evaluated a more limited range of assays (based on the mass cytometry panels), compared to broader profiling by transcriptome. It is important to also note that transcriptome and proteome do not always agree, justifying the need to evaluate both. Interestingly, another study suggests that immune changes associated with preterm birth may be durable, as women with a history of preterm birth (but who were not actually pregnant at the time of evaluation) had alterations in innate immune signaling function, particularly classical monocytes (30).

Our study provides insight into potential immune mechanisms that could drive preterm birth, but did not identify a clear biomarker for preterm birth akin to the signature recently identified using analysis of cell-free DNA (31). In fact, it will be important to validate such signatures in cohorts drawn from a range of geographic areas, with a particular focus on Africa and Asia where preterm birth is most prevalent. New approaches to evaluate transcriptomic signatures at the single cell level have the potential to improve our understanding of the maternal fetal interface and better identify the mechanisms driving preterm birth (62–64).

This study has several important limitations, the first of which is the relatively modest sample size and availability of good quality PBMC and RNA samples for these analyses. Particularly in light of the multiple distinct risk factors for preterm birth, which could be operating through distinct immune pathways, this definitely hinders our ability to find a clear signal. Another possible limitation was highlighted by a recent paper demonstrating that Affymetrix arrays were the least sensitive method to identify signatures associated with preterm birth in placental tissues (23). We hope that by placing this data in the public domain, others will be able to combine it with other datasets for more robust analyses. Another limitation is that culturing the cells will change the transcriptome, though we controlled for this by treating unstimulated and stimulated samples identically. Finally, we did not have universal ultrasound available for gestational dating in the low-middle income setting. This could have led to misclassification of preterm vs. term births, and was one of the reasons for our secondary analysis of time to delivery, as that timing was known with greater precision.

Overall, many risk factors for preterm birth are well-characterized, and many could drive preterm birth by inducing changes in immune function. Here we sought to identify specific immune pathways associated with preterm birth in women in a setting of high preterm birth prevalence in Kenya. While we did not identify a clear signature of preterm birth, we did find that the IFN response following a viral stimulation is temporally regulated during pregnancy, with reduced IFN induction as delivery approaches. This adds to our knowledge of immune regulation during pregnancy, but more study is clearly needed to further understand this complex immunologic time.

## Data Availability Statement

The datasets generated for this study can be found in the Gene Expression Omnibus (GEO) under accession numbers GSE140939.

## Ethics Statement

All subjects gave informed and written consent. All studies were performed in accordance with the Declaration of Helsinki, and all study procedures were approved by the Institutional Review Boards of the University of Washington, Seattle, WA, USA and Kenyatta National Hospital, Nairobi, Kenya.

## Author Contributions

NB, GJ-S, and CB conceptualized this study. JP, JK, LO, DM, BR, and GJ-S developed and maintained the cohort. NB and CB designed experiments. NB and RV conducted experiments. CS, NB, RV, JP, BR, and SH analyzed the data. CS, NB, and CB wrote the manuscript. All authors contributed to revisions and approved the manuscript.

### Conflict of Interest

The authors declare that the research was conducted in the absence of any commercial or financial relationships that could be construed as a potential conflict of interest.
