# Supplementary material for: Influenza-Induced Interferon Lambda Response Is Associated With Longer Time to Delivery Among Pregnant Kenyan Women
Source: Front Immunol. 2020 Mar 17;11:452. doi: 10.3389/fimmu.2020.00452 (PMC7089959; doi:10.3389/fimmu.2020.00452)
Supplement: Supplementary file 1 [file Table_1.docx]

**Supplemental Table 1**. Differentially expressed genes in stimulated samples for time to deliver term at FDR of 0.1. We included the following covariates in the linear model: intercept, time to delivery, and gestational age at birth. In the table, we show the top 170 genes for the time to delivery term. See Figure 2 for a full network analysis on all genes.

| Gene symbol | Coefficient (log2) | Average expression (log2) | Unadjusted p-value | Adjusted p-value |
| --- | --- | --- | --- | --- |
| IFNL1 | -0.06 | 4.9 | 8e-07 | 0.018 |
| NEURL1B | -0.03 | 4.4 | 2e-06 | 0.020 |
| INPP5A | 0.03 | 4.7 | 3e-06 | 0.020 |
| SNORD18A | 0.13 | 5.7 | 4e-06 | 0.024 |
| KRT33A | -0.02 | 4.8 | 1e-05 | 0.049 |
| PAQR4 | -0.03 | 5.2 | 1e-05 | 0.049 |
| MICB | -0.03 | 5.6 | 2e-05 | 0.057 |
| RBMS2 | -0.03 | 5.0 | 2e-05 | 0.058 |
| ATP1A4 | -0.03 | 4.7 | 2e-05 | 0.058 |
| GAS6 | -0.02 | 5.0 | 3e-05 | 0.061 |
| CISD3 | -0.03 | 5.7 | 3e-05 | 0.069 |
| TMEM150B | -0.03 | 5.3 | 4e-05 | 0.069 |
| ETV4 | -0.03 | 4.5 | 4e-05 | 0.069 |
| MTX1 | -0.02 | 5.3 | 4e-05 | 0.069 |
| IFNAR1 | -0.02 | 3.6 | 5e-05 | 0.069 |
| ASPRV1 | -0.03 | 4.1 | 6e-05 | 0.069 |
| MMP2 | -0.02 | 5.3 | 6e-05 | 0.069 |
| TOGARAM1 | 0.03 | 3.6 | 7e-05 | 0.069 |
| BOLA1 | -0.02 | 6.1 | 7e-05 | 0.069 |
| HLA-DQB2 | -0.03 | 5.7 | 7e-05 | 0.069 |
| HLA-DQB2 | -0.03 | 5.7 | 7e-05 | 0.069 |
| IL22RA1 | -0.03 | 4.7 | 7e-05 | 0.069 |
| MICB | -0.03 | 5.4 | 8e-05 | 0.071 |
| MS4A15 | -0.02 | 4.6 | 8e-05 | 0.071 |
| KCTD5 | 0.03 | 6.1 | 8e-05 | 0.071 |
| FRY | 0.02 | 3.8 | 9e-05 | 0.072 |
| SNORD4B | 0.08 | 6.0 | 9e-05 | 0.072 |
| PRG2 | -0.02 | 3.7 | 1e-04 | 0.072 |
| CIB2 | -0.04 | 6.2 | 1e-04 | 0.072 |
| RNPC3 | 0.04 | 4.8 | 1e-04 | 0.072 |
| NBR2 | 0.02 | 4.8 | 1e-04 | 0.072 |
| LSP1P5 | -0.02 | 4.8 | 1e-04 | 0.075 |
| LINC00630 | 0.02 | 3.7 | 1e-04 | 0.075 |
| TCTN3 | 0.04 | 5.1 | 1e-04 | 0.075 |
| MICA | -0.02 | 5.1 | 1e-04 | 0.075 |
| NTN3 | -0.02 | 5.6 | 1e-04 | 0.075 |
| IL27 | -0.03 | 5.8 | 1e-04 | 0.075 |
| MAPK8 | 0.03 | 5.2 | 1e-04 | 0.075 |
| FOXD4L6 | -0.03 | 5.4 | 1e-04 | 0.077 |
| KCNG4 | -0.02 | 4.6 | 1e-04 | 0.077 |
| SLC16A8 | -0.02 | 5.3 | 1e-04 | 0.077 |
| PRR19 | -0.03 | 4.4 | 2e-04 | 0.077 |
| ELP1 | 0.03 | 5.1 | 2e-04 | 0.077 |
| MIR210HG | -0.04 | 5.8 | 2e-04 | 0.077 |
| TNK2 | -0.02 | 6.5 | 2e-04 | 0.077 |
| ULK4 | 0.02 | 4.1 | 2e-04 | 0.077 |
| LY6D | -0.02 | 5.2 | 2e-04 | 0.077 |
| MIR596 | -0.04 | 5.9 | 2e-04 | 0.077 |
| EPOP | -0.03 | 4.8 | 2e-04 | 0.077 |
| LHFPL4 | -0.02 | 5.2 | 2e-04 | 0.077 |
| TBC1D3P1-DHX40P1 | 0.02 | 4.8 | 2e-04 | 0.077 |
| LRRC30 | -0.03 | 4.2 | 2e-04 | 0.077 |
| ARHGAP27 | -0.02 | 5.6 | 2e-04 | 0.077 |
| FCN2 | -0.02 | 6.2 | 2e-04 | 0.077 |
| MIR210HG | -0.06 | 6.0 | 2e-04 | 0.077 |
| PWAR5 | 0.05 | 3.8 | 2e-04 | 0.077 |
| MATN4 | -0.02 | 4.9 | 2e-04 | 0.077 |
| TNKS | 0.03 | 4.5 | 2e-04 | 0.077 |
| ADAM33 | -0.03 | 5.7 | 2e-04 | 0.077 |
| HLA-DQB2 | -0.03 | 5.7 | 2e-04 | 0.077 |
| HLA-DQB2 | -0.03 | 5.7 | 2e-04 | 0.077 |
| SNORD20 | 0.05 | 5.8 | 2e-04 | 0.077 |
| AHI1 | 0.03 | 3.5 | 2e-04 | 0.077 |
| RNU4ATAC | 0.08 | 10.0 | 2e-04 | 0.077 |
| NFS1 | 0.01 | 5.0 | 2e-04 | 0.077 |
| CTD-2297D10.2 | -0.03 | 4.7 | 2e-04 | 0.077 |
| PNPLA5 | -0.02 | 4.5 | 2e-04 | 0.077 |
| THTPA | -0.03 | 5.3 | 2e-04 | 0.077 |
| CDK18 | -0.03 | 5.2 | 2e-04 | 0.077 |
| BEGAIN | -0.02 | 4.9 | 2e-04 | 0.077 |
| SLC2A10 | -0.03 | 4.5 | 3e-04 | 0.079 |
| OR7E91P | -0.02 | 4.1 | 3e-04 | 0.079 |
| SLC25A47 | -0.02 | 5.2 | 3e-04 | 0.079 |
| ATP23 | 0.03 | 5.1 | 3e-04 | 0.079 |
| C1QB | -0.03 | 5.5 | 3e-04 | 0.079 |
| PRKCI | 0.03 | 4.5 | 3e-04 | 0.084 |
| PFDN4 | 0.02 | 4.3 | 3e-04 | 0.085 |
| DRD5 | -0.03 | 5.2 | 3e-04 | 0.089 |
| HIST2H2AC | 0.04 | 10.0 | 3e-04 | 0.089 |
| SLC27A3 | -0.02 | 5.2 | 3e-04 | 0.089 |
| FBXL20 | 0.03 | 3.6 | 3e-04 | 0.089 |
| CDK8 | 0.03 | 4.4 | 3e-04 | 0.090 |
| HLA-DQB2 | -0.03 | 5.6 | 3e-04 | 0.091 |
| HLA-DQB2 | -0.03 | 5.6 | 3e-04 | 0.091 |
| GLCE | 0.02 | 3.5 | 4e-04 | 0.091 |
| MIR3178 | -0.03 | 5.8 | 4e-04 | 0.091 |
| ISOC2 | -0.02 | 5.5 | 4e-04 | 0.091 |
| FAM90A8P | -0.03 | 5.2 | 4e-04 | 0.091 |
| TMEM249 | -0.02 | 4.9 | 4e-04 | 0.092 |
| NUS1P3 | 0.03 | 4.2 | 4e-04 | 0.092 |
| TMEM9 | -0.02 | 5.2 | 4e-04 | 0.092 |
| PTBP2 | 0.03 | 4.1 | 4e-04 | 0.092 |
| TTC3P1 | -0.02 | 3.9 | 4e-04 | 0.092 |
| SCRN2 | -0.02 | 5.4 | 4e-04 | 0.092 |
| GRM6 | -0.03 | 4.8 | 4e-04 | 0.092 |
| MIR4277 | -0.03 | 4.9 | 4e-04 | 0.092 |
| SMTNL2 | -0.02 | 5.0 | 4e-04 | 0.092 |
| NRXN2 | -0.02 | 5.2 | 4e-04 | 0.092 |
| TGM2 | -0.03 | 5.6 | 4e-04 | 0.092 |
| ADAD2 | -0.02 | 5.4 | 4e-04 | 0.092 |
| FNDC8 | -0.02 | 3.8 | 4e-04 | 0.092 |
| TPBGL | -0.03 | 4.3 | 4e-04 | 0.092 |
| TMEM161B | 0.02 | 3.8 | 4e-04 | 0.092 |
| SMKR1 | -0.02 | 5.3 | 4e-04 | 0.092 |
| ITGA3 | -0.01 | 5.3 | 4e-04 | 0.092 |
| RTTN | 0.02 | 3.5 | 4e-04 | 0.092 |
| RARG | -0.03 | 5.5 | 5e-04 | 0.092 |
| TMEM229B | -0.03 | 5.9 | 5e-04 | 0.092 |
| IFNA8 | -0.13 | 4.2 | 5e-04 | 0.092 |
| MIR3650 | -0.03 | 5.6 | 5e-04 | 0.092 |
| UBR3 | 0.03 | 4.3 | 5e-04 | 0.092 |
| ITGA9 | -0.02 | 4.4 | 5e-04 | 0.092 |
| PRKCA | 0.05 | 6.1 | 5e-04 | 0.092 |
| MUC6 | -0.02 | 5.5 | 5e-04 | 0.092 |
| OLIG2 | -0.02 | 4.7 | 5e-04 | 0.092 |
| PDGFRL | -0.04 | 5.4 | 5e-04 | 0.092 |
| KPNB1 | -0.06 | 5.4 | 5e-04 | 0.092 |
| BHLHA15 | -0.02 | 5.9 | 5e-04 | 0.092 |
| LOC105373383 | -0.03 | 4.8 | 5e-04 | 0.092 |
| MICB | -0.03 | 6.0 | 5e-04 | 0.092 |
| GPR3 | -0.03 | 4.5 | 5e-04 | 0.092 |
| SDS | -0.02 | 4.8 | 5e-04 | 0.092 |
| UBQLNL | -0.03 | 3.5 | 5e-04 | 0.092 |
| ANGPTL4 | -0.03 | 5.0 | 5e-04 | 0.092 |
| MICB | -0.04 | 6.1 | 5e-04 | 0.092 |
| THAP11 | -0.03 | 4.1 | 5e-04 | 0.092 |
| IFNL3 | -0.03 | 5.5 | 5e-04 | 0.092 |
| FKBP2 | -0.02 | 7.2 | 5e-04 | 0.092 |
| OR5B21 | 0.05 | 5.5 | 5e-04 | 0.092 |
| TTPAL | 0.02 | 4.4 | 5e-04 | 0.092 |
| HLA-DQB2 | -0.02 | 5.6 | 5e-04 | 0.092 |
| HLA-DQB2 | -0.02 | 5.6 | 5e-04 | 0.092 |
| ODF3L1 | -0.02 | 3.8 | 6e-04 | 0.093 |
| MICB | -0.03 | 6.0 | 6e-04 | 0.093 |
| ZNF710 | -0.02 | 5.6 | 6e-04 | 0.093 |
| SYCN | -0.03 | 5.1 | 6e-04 | 0.093 |
| TMEM176B | -0.02 | 5.8 | 6e-04 | 0.093 |
| NCR3LG1 | 0.05 | 4.7 | 6e-04 | 0.094 |
| MIR338 | -0.03 | 4.4 | 6e-04 | 0.095 |
| ALPP | -0.02 | 5.0 | 6e-04 | 0.095 |
| ATF5 | -0.03 | 6.2 | 6e-04 | 0.095 |
| MYMK | -0.02 | 5.4 | 6e-04 | 0.095 |
| HRCT1 | -0.02 | 5.9 | 6e-04 | 0.095 |
| ST7L | 0.02 | 3.6 | 6e-04 | 0.095 |
| RRBP1 | -0.04 | 6.6 | 6e-04 | 0.095 |
| NARF | -0.03 | 6.4 | 6e-04 | 0.096 |
| PLEKHA1 | 0.02 | 5.0 | 6e-04 | 0.096 |
| ZNF385A | -0.02 | 5.2 | 6e-04 | 0.096 |
| LRG1 | -0.02 | 5.1 | 7e-04 | 0.096 |
| ANGPTL6 | -0.02 | 5.1 | 7e-04 | 0.096 |
| FAF1 | 0.02 | 4.3 | 7e-04 | 0.096 |
| NPW | -0.02 | 5.0 | 7e-04 | 0.096 |
| LINC01619 | 0.03 | 3.8 | 7e-04 | 0.096 |
| IFNA17 | -0.15 | 3.9 | 7e-04 | 0.096 |
| LINC00566 | 0.02 | 4.2 | 7e-04 | 0.096 |
| ACADS | -0.02 | 5.3 | 7e-04 | 0.096 |
| NEK1 | 0.03 | 4.5 | 7e-04 | 0.096 |
| ERVH48-1 | -0.03 | 4.2 | 7e-04 | 0.096 |
| ZNF296 | -0.03 | 5.2 | 7e-04 | 0.096 |
| ANKRA2 | 0.02 | 5.1 | 7e-04 | 0.096 |
| AGXT | -0.02 | 5.3 | 7e-04 | 0.099 |
| FAM168A | -0.02 | 4.9 | 7e-04 | 0.099 |
| MOB3B | -0.03 | 5.0 | 7e-04 | 0.099 |
| NAF1 | 0.06 | 5.3 | 7e-04 | 0.099 |
| TFAP2E | -0.02 | 4.9 | 7e-04 | 0.099 |
| MROH6 | -0.02 | 6.0 | 8e-04 | 0.099 |
| TRIB3 | -0.02 | 5.5 | 8e-04 | 0.099 |
| CCNQP1 | -0.02 | 5.5 | 8e-04 | 0.099 |
| FUOM | -0.02 | 5.6 | 8e-04 | 0.099 |
| GHRL | -0.02 | 4.7 | 8e-04 | 0.099 |

**Supplementary Figures**

**
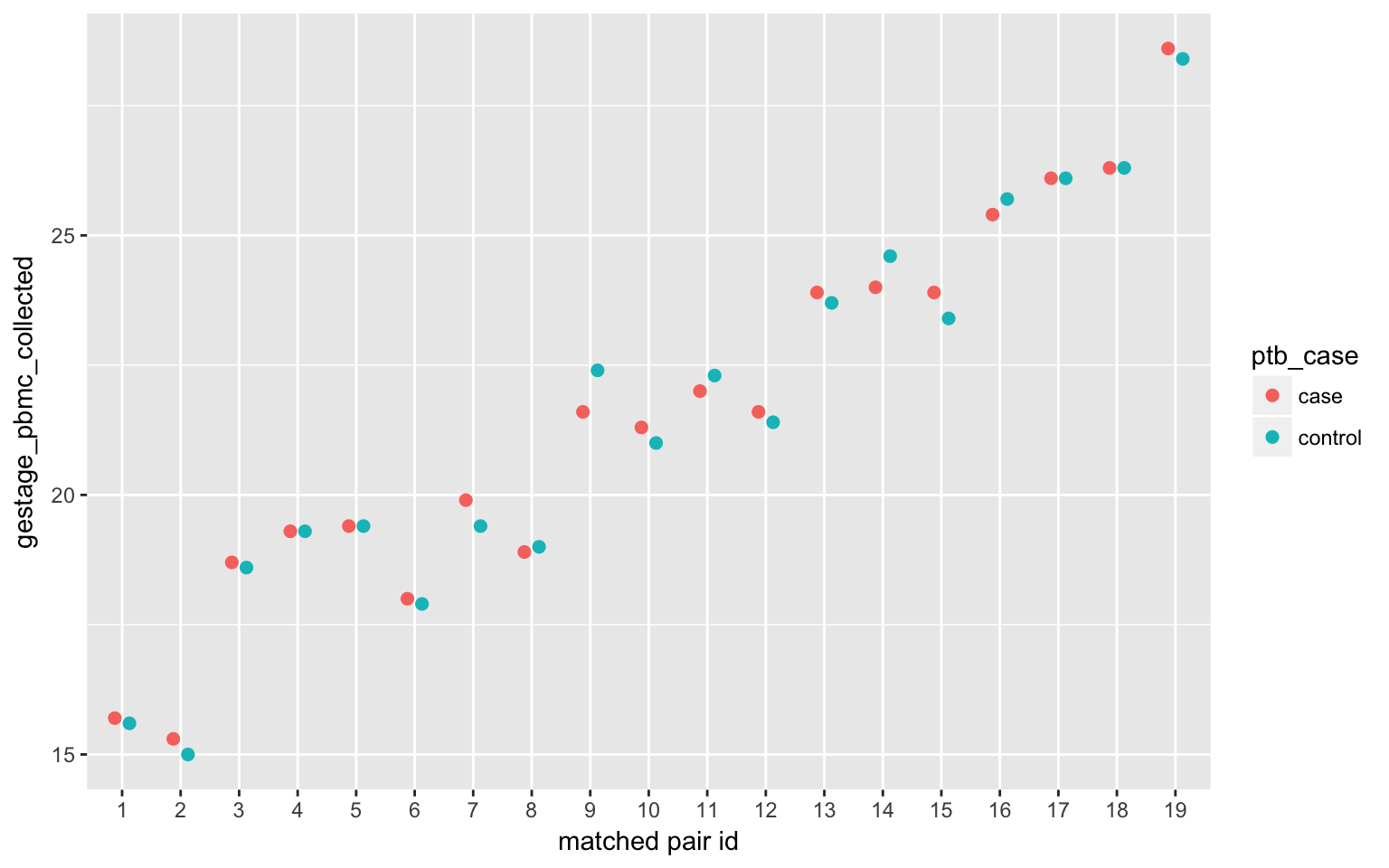
**

**Supplementary Figure 1A:** Matched stimulated samples with the smallest average absolute distance across all the matched pairs.


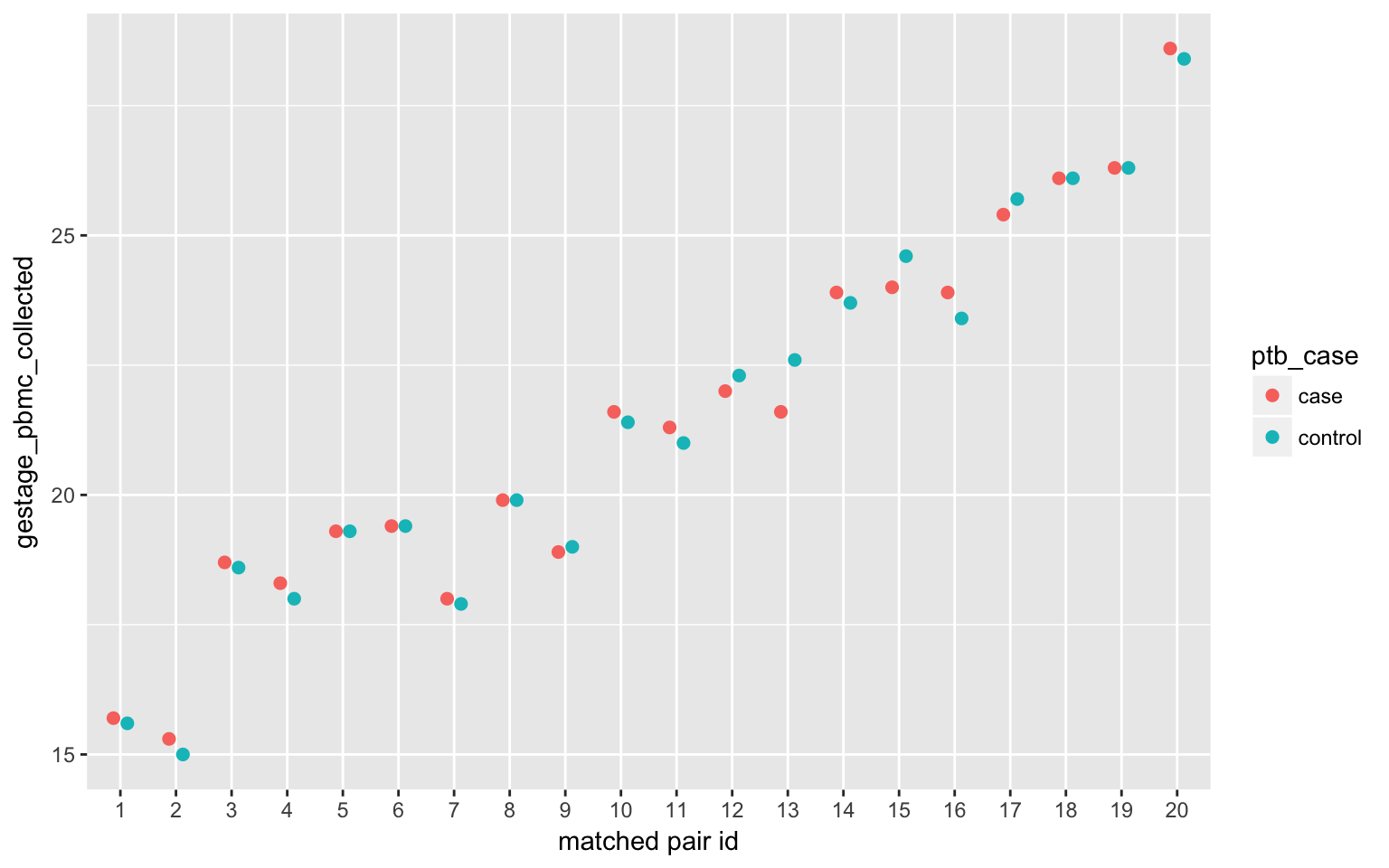
**Supplementary Figure 1B:** Matched unstimulated samples with the smallest average absolute distance across all the matched pairs.


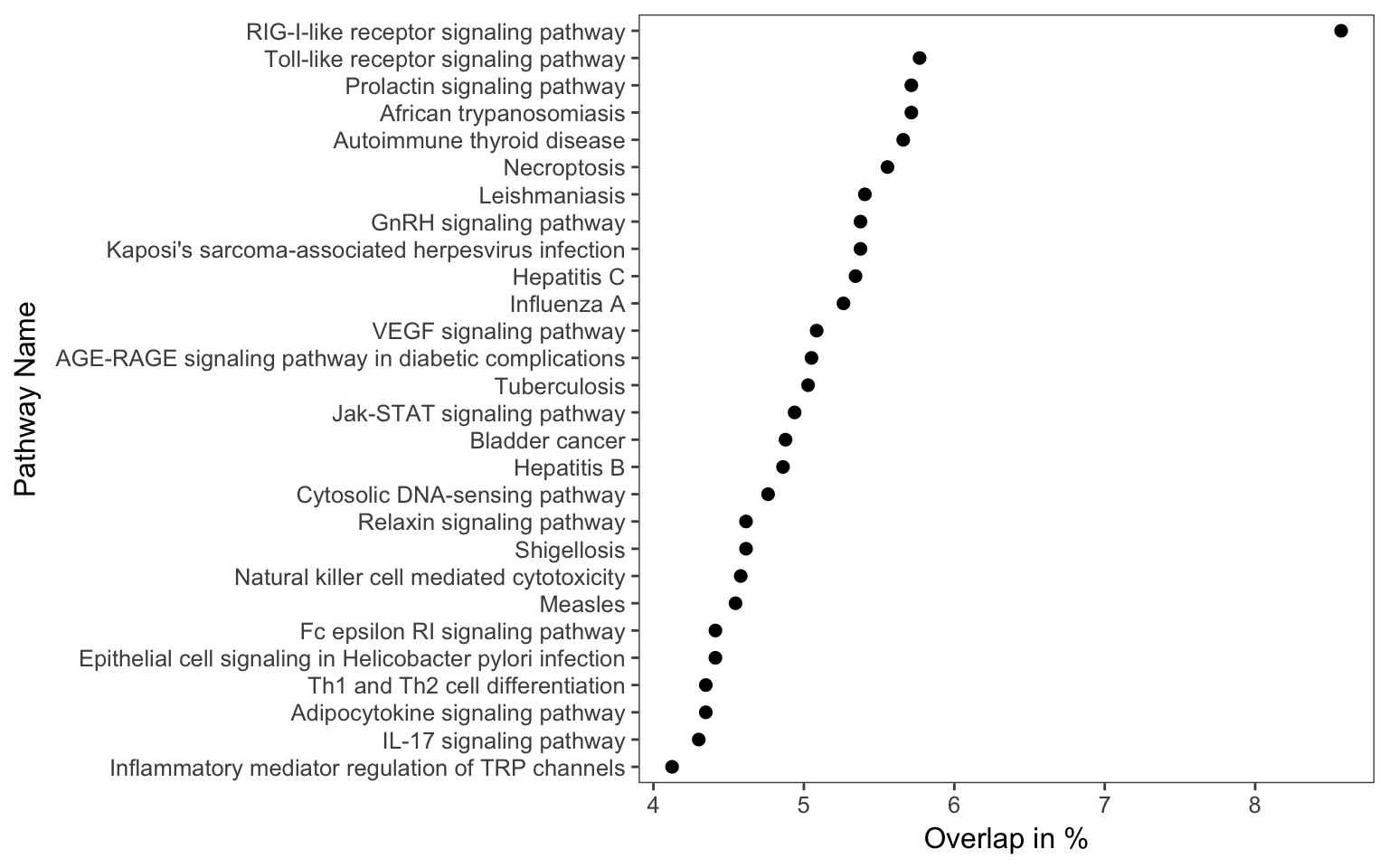


**Supplementary Figure 2.** Pathway analysis. Overlap between our discovered network with known pathways in KEGG. Number of genes that our network on known pathways have in common. We only show pathways with at least 5% gene overlap.
