# Supplementary material for: Influenza-Induced Interferon Lambda Response Is Associated With Longer Time to Delivery Among Pregnant Kenyan Women
Source: Front Immunol. 2020 Mar 17;11:452. doi: 10.3389/fimmu.2020.00452 (PMC7089959; doi:10.3389/fimmu.2020.00452)
Supplement: Supplementary file 2 [file Presentation_1.zip › CaseControl_Microarray_Combined.pdf]

Case-Control with Gestational Age Matching

Christof Seiler

18 November 2019

1 Combine Tables

Combine sample tables from stimulated and unstimulated matchings for table in paper.

```
library("readr")
library("dplyr")
library("magrittr")
library("ggplot2")
sample_table_matched_stim = read_csv("sample_table_matched_stim.csv")
sample_table_matched_unstim = read_csv("sample_table_matched_unstim.csv")
sample_table = bind_rows(sample_table_matched_stim,
                         sample_table_matched_unstim)
sample_table %<>% distinct(ptidno, .keep_all = TRUE)
sample_table %<>% dplyr::select(-pair)
write_csv(sample_table,path = "sample_table_case_control_combined.csv")
```

Characteristics of the study population.

```
sample_table %<>% mutate(
  time_to_delivery = gestage_pbmc_collected - gestage_delivery
  )
sample_table %>%
  group_by(ptb_case) %>%
  summarize(median_time_to_delivery = median(time_to_delivery))
## # A tibble: 2 x 2
##   ptb_case median_time_to_delivery
##   <chr>                      <dbl>
## 1 case                        -6.7
## 2 control                    -19
wilcox.test(time_to_delivery ~ ptb_case, data = sample_table)
## Warning in wilcox.test.default(x = c(-5.6, -18.3, -14.7, -11.6, -10.2,
## -14.9, : cannot compute exact p-value with ties
##
##  Wilcoxon rank sum test with continuity correction
##
## data:  time_to_delivery by ptb_case
## W = 417.5, p-value = 6.98e-07
## alternative hypothesis: true location shift is not equal to 0
ggplot(sample_table, aes(time_to_delivery, fill = ptb_case)) +
  geom_histogram(bins = 10, alpha = 0.5, position = "identity")
```

Case-Control with Gestational Age Matching

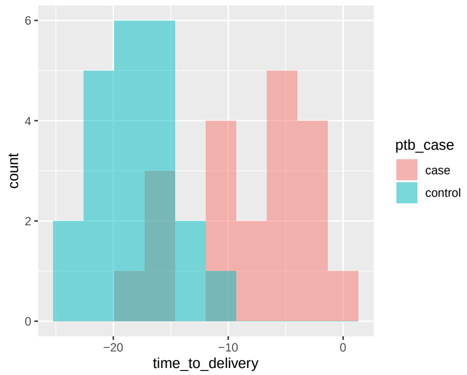

Session info.

```
sessionInfo()
## R version 3.5.1 (2018-07-02)
## Platform: x86_64-apple-darwin15.6.0 (64-bit)
## Running under: macOS  10.15.1
##
## Matrix products: default
## BLAS: /Library/Frameworks/R.framework/Versions/3.5/Resources/lib/libRblas.0.dylib
## LAPACK: /Library/Frameworks/R.framework/Versions/3.5/Resources/lib/libRlapack.dylib
##
## locale:
## [1] en_US.UTF-8/en_US.UTF-8/en_US.UTF-8/C/en_US.UTF-8/en_US.UTF-8
##
## attached base packages:
## [1] stats     graphics  grDevices utils     datasets  methods   base
##
## other attached packages:
## [1] ggplot2_3.2.1    magrittr_1.5     dplyr_0.8.3      readr_1.3.1
## [5] BiocStyle_2.10.0
##
## loaded via a namespace (and not attached):
##  [1] Rcpp_1.0.3         pillar_1.4.2       compiler_3.5.1
##  [4] BiocManager_1.30.4 tools_3.5.1        zeallot_0.1.0
##  [7] digest_0.6.22      evaluate_0.13      tibble_2.1.3
## [10] gtable_0.3.0       pkgconfig_2.0.3    rlang_0.4.1
## [13] cli_1.1.0          yaml_2.2.0         xfun_0.6
## [16] withr_2.1.2        stringr_1.4.0      knitr_1.22
## [19] vctrs_0.2.0        hms_0.5.2          grid_3.5.1
## [22] tidyselect_0.2.5   glue_1.3.1         R6_2.4.1
## [25] fansi_0.4.0        rmarkdown_1.12     bookdown_0.9
## [28] purrr_0.3.3        backports_1.1.5    scales_1.0.0
## [31] htmltools_0.3.6    assertthat_0.2.1   colorspace_1.4-1
## [34] labeling_0.3       utf8_1.1.4         stringi_1.4.3
## [37] lazyeval_0.2.2     munsell_0.5.0      crayon_1.3.4
```
