# Supplementary material for: Influenza-Induced Interferon Lambda Response Is Associated With Longer Time to Delivery Among Pregnant Kenyan Women
Source: Front Immunol. 2020 Mar 17;11:452. doi: 10.3389/fimmu.2020.00452 (PMC7089959; doi:10.3389/fimmu.2020.00452)
Supplement: Supplementary file 2 [file Presentation_1.zip › CaseControl_Microarray_Unstim.pdf]

Case-Control with Gestational Age Matching

Christof Seiler

12 February 2020

Contents

1 Goal . . . 2
2 Prerequisites . . . 2
3 Import Data . . . 2
4 Quality Control . . . 7
5 Preprocessing . . . 8
6 Data Exploration . . . 9
7 Differential Expression Analyses . . . 11
8 Patient Variability . . . 15
9 Power Analysis . . . 18
10 Gene Set Enrichment Analysis . . . 20
Session Info . . . 20

1 Goal

1. Load and normalize data using `oligo`
2. Differential analysis using `limma`

2 Prerequisites

Install necessary packages from bioconductor repository. Run this code only once to install packages.

```
pkgs_needed = c("oligo","limma","hta20transcriptcluster.db","pd.hta.2.0",
                "affycoretools","genefilter","MatchIt","optmatch","ggfortify",
                "magrittr","statmod","readr","dplyr","readxl","stringr",
                "tibble","ggrepel","tidyr","locfdr")
letsinstall = setdiff(pkgs_needed, installed.packages())
if (length(letsinstall) > 0) {
  source("http://bioconductor.org/biocLite.R")
  biocLite(letsinstall)
}
```

Load packages.

```
library("oligo")
library("limma")
library("hta20transcriptcluster.db")
library("affycoretools")
library("genefilter")
library("MatchIt")
library("ggfortify")
library("magrittr")
library("statmod")
library("readr")
library("dplyr")
library("readxl")
library("stringr")
library("tibble")
library("ggrepel")
library("tidyr")
library("locfdr")
```

3 Import Data

Read sample tables. Merge with sample information table.

```
sample_table = read_csv("sample_table_from_word.csv")
## Parsed with column specification:
## cols(
##   ptidno = col_double(),
##   visitcode = col_double(),
##   gestage_delivery = col_double(),
```

```
##   gestage_pbmc_collected = col_double(),
##   ptb_case = col_character(),
##   ptb_casenum = col_double(),
##   ptb_controlnum = col_double()
## )
new_matched = read_excel("MSS Case_Control Matches (Masked)_new_matched.xlsx")
sample_table %<>% dplyr::left_join(new_matched,by = "ptidno",
                                   suffix = c("", "2"))
ggplot(sample_table, aes(as.factor(ptb_casenum), gestage_pbmc_collected,
                         color = ptb_case)) +
  geom_jitter(position=position_dodge(width = 0.5),size = 2) +
  xlab("matched pair id")
```

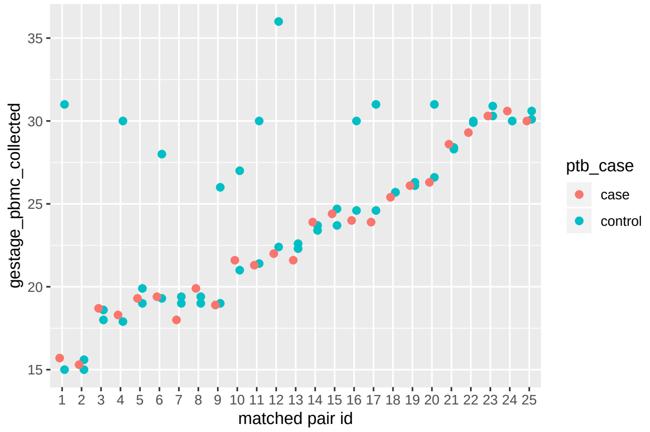


```
sample_table %<>% dplyr::select(ptidno,
                                ptb_case,
                                gestage_pbmc_collected,
                                gestage_delivery,
                                sample_id = SampleID)
```

Find CEL files in current folder.

```
file_names_cel = list.files("./",pattern = "CEL")
params$treatment
## [1] "unstim"
if(params$treatment == "stim") {
  file_names_cel = file_names_cel[str_detect(
    string = file_names_cel,
    pattern = "Bayless_H1N1|\\+\\_\\(HTA")]
} else {
  file_names_cel = file_names_cel[str_detect(
    string = file_names_cel,
    pattern = "Bayless_US|\\-\\_\\(HTA|\\_\\_\\(HTA|\\-\\_\\(HTA")]
}
tb_file_name = lapply(sample_table$sample_id,function(id) {
  pattern = paste0("[ _]",id)
  name = file_names_cel[which(str_detect(file_names_cel,pattern))]
  if(length(name)==0) name = NA
```

```
  tibble(sample_id = id,file_name = name)
}) %>% bind_rows()
sample_table %<>% dplyr::left_join(tb_file_name,by = "sample_id")
sample_table %<>% na.omit
table(sample_table$ptb_case)
##
##    case control
##      20      38
```

Match samples.

```
set.seed(0xdada2)
sample_table$group = sample_table$ptb_case == "case"
mout = matchit(group ~ gestage_pbmc_collected,
               data = sample_table,
               method = "optimal",
               ratio = 1)
## Warning in optmatch::fullmatch(d, min.controls = ratio, max.controls = ratio, : Without 'data' argument th
##     to be the same as your original data.
summary(mout)
##
## Call:
## matchit(formula = group ~ gestage_pbmc_collected, data = sample_table,
##     method = "optimal", ratio = 1)
##
## Summary of balance for all data:
##                        Means Treated Means Control SD Control Mean Diff
## distance                      0.3752        0.3288     0.1148    0.0464
## gestage_pbmc_collected       21.4100       23.6605     5.2473   -2.2505
##                        eQQ Med eQQ Mean eQQ Max
## distance                0.0586   0.0525  0.1042
## gestage_pbmc_collected  1.8000   2.2350  7.4000
##
##
## Summary of balance for matched data:
##                        Means Treated Means Control SD Control Mean Diff
## distance                      0.3752        0.3753     0.0887    -1e-04
## gestage_pbmc_collected       21.4100       21.4100     3.6864     0e+00
##                        eQQ Med eQQ Mean eQQ Max
## distance                0.0041   0.0055   0.017
## gestage_pbmc_collected  0.2000   0.2300   0.700
##
## Percent Balance Improvement:
##                        Mean Diff. eQQ Med eQQ Mean eQQ Max
## distance                  99.7989 93.0472  89.6035 83.7009
## gestage_pbmc_collected   100.0000 88.8889  89.7092 90.5405
##
## Sample sizes:
##           Control Treated
## All            38      20
## Matched        20      20
```

```
## Unmatched      18       0
## Discarded       0       0
sample_table$pair = NA
A = rownames(mout$match.matrix) %>% as.integer
B = mout$match.matrix %>% as.integer
for(i in 1:nrow(mout$match.matrix)) {
  sample_table$pair[A[i]] = i
  sample_table$pair[B[i]] = i
}
sample_table %<>% dplyr::select(-group)
sample_table %<>% na.omit
sample_table %>%
  dplyr::select(sample_id,ptidno,pair,ptb_case,gestage_pbmc_collected,
                gestage_delivery) %>%
  arrange(pair,ptb_case) %>%
  print(n = Inf)
## # A tibble: 40 x 6
##    sample_id   ptidno  pair ptb_case gestage_pbmc_collected gestage_delivery
##    <chr>        <dbl> <int> <chr>                     <dbl>            <dbl>
##  1 1.2       14104760     1 case                       15.7             21.3
##  2 2.2       14104580     1 control                    15.6             37.4
##  3 2.1       14101980     2 case                       15.3             33.6
##  4 2.3       14105160     2 control                    15               39.3
##  5 3.3       14225030     3 case                       18.7             33.4
##  6 3.2       14223910     3 control                    18.6             38
##  7 4.2       14100420     4 case                       18.3             33
##  8 3.1       14105820     4 control                    18               38
##  9 5.3       14222170     5 case                       19.3             30.9
## 10 6.3       14223900     5 control                    19.3             39
## 11 6.1       14220060     6 case                       19.4             29.6
## 12 8.1       14104890     6 control                    19.4             41
## 13 7.2       14100460     7 case                       18               32.9
## 14 4.3       14102700     7 control                    17.9             40
## 15 8.3       14224240     8 case                       19.9             23
## 16 5.2       14103060     8 control                    19.9             42.3
## 17 9.2       14102840     9 case                       18.9             24.1
## 18 9.1       14102770     9 control                    19               38.3
## 19 10.1      14223520    10 case                       21.6             27.9
## 20 11.1      14104710    10 control                    21.4             39.9
## 21 11.2      14102560    11 case                       21.3             31.9
## 22 10.3      14224520    11 control                    21               37.6
## 23 12.1      14220350    12 case                       22               27.3
## 24 13.1      14100770    12 control                    22.3             39.1
## 25 13.2      14224670    13 case                       21.6             26.7
## 26 13.3      14100390    13 control                    22.6             37.3
## 27 14.3      14222790    14 case                       23.9             31
## 28 14.2      14105140    14 control                    23.7             43
## 29 16.1      14102780    15 case                       24               33.7
## 30 17.3      14100120    15 control                    24.6             39.6
## 31 17.2      14105630    16 case                       23.9             31.3
## 32 14.1      14100400    16 control                    23.4             39.6
```

```
## 33 18.3       14222340    17 case                          25.4            27.6
## 34 18.1       14104350    17 control                       25.7            38.4
## 35 19.1       14103660    18 case                          26.1            29.1
## 36 19.3       14102370    18 control                       26.1            41
## 37 20.2       14100230    19 case                          26.3            26.7
## 38 19.2       14225270    19 control                       26.3            39
## 39 21.3       14106020    20 case                          28.6            31.7
## 40 21.2       14220100    20 control                       28.4            39
write_csv(sample_table,path = paste0("sample_table_matched_",params$treatment,".csv"))
ggplot(sample_table, aes(as.factor(pair), gestage_pbmc_collected,
                         color = ptb_case)) +
  geom_jitter(position=position_dodge(width = 0.5),size = 2) +
  xlab("matched pair id")
```

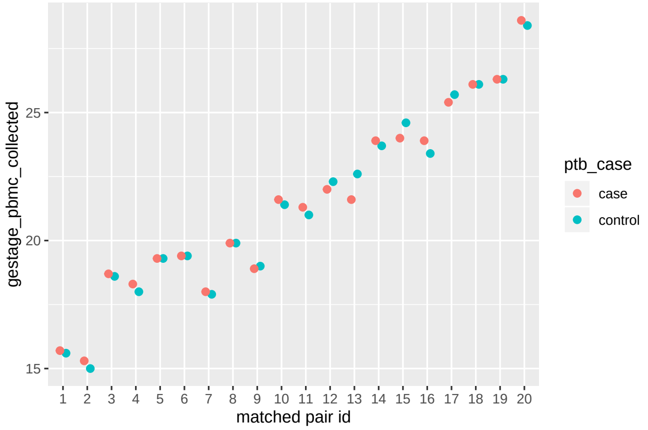

Then load Affymetrix CEL files. At this stage, Bioconductor will automatically download the necessary annotation packages and install them for us.

```
pd = as(as.data.frame(sample_table), "AnnotatedDataFrame")
rawData = read.celfiles(sample_table$file_name,
                        phenoData = pd,
                        sampleNames = sample_table$sample_id)
## Loading required package: pd.hta.2.0
## Loading required package: RSQLite
## Loading required package: DBI
## Platform design info loaded.
## Reading in : Nicholas Bayless_US 1.2_(HTA-2_0).CEL
## Reading in : Nicholas Bayless_US 2.1_(HTA-2_0).CEL
## Reading in : Nicholas Bayless_US 2.3_(HTA-2_0).CEL
## Reading in : Nicholas Bayless_US 2.2_(HTA-2_0).CEL
## Reading in : Nicholas Bayless_US 3.3_(HTA-2_0).CEL
## Reading in : Nicholas Bayless_US 3.2_(HTA-2_0).CEL
## Reading in : Nicholas Bayless_US 3.1_(HTA-2_0).CEL
## Reading in : Nicholas Bayless_US 4.2_(HTA-2_0).CEL
## Reading in : Nicholas Bayless_US 4.3_(HTA-2_0).CEL
## Reading in : Nicholas Bayless_US 5.3_(HTA-2_0).CEL
## Reading in : Nicholas Bayless_US 5.2_(HTA-2_0).CEL
## Reading in : Nicholas Bayless_US 6.1_(HTA-2_0).CEL
```

```
## Reading in : Nicholas Bayless_US 6.3_(HTA-2_0).CEL
## Reading in : Nicholas Bayless_7.2 -_(HTA-2_0).CEL
## Reading in : Nicholas Bayless_8.3 -_(HTA-2_0).CEL
## Reading in : Nicholas Bayless_8.1 -_(HTA-2_0).CEL
## Reading in : Nicholas Bayless_9.2 -_(HTA-2_0).CEL
## Reading in : Nicholas Bayless_9.1 -_(HTA-2_0).CEL
## Reading in : Nicholas Bayless_10.1 -_(HTA-2_0).CEL
## Reading in : Nicholas Bayless_10.3 -_(HTA-2_0).CEL
## Reading in : Nicholas Bayless_11.2 -_(HTA-2_0).CEL
## Reading in : Nicholas Bayless_11.1 -_(HTA-2_0).CEL
## Reading in : Nicholas Bayless_12.1 -_(HTA-2_0).CEL
## Reading in : Nicholas Bayless_US 13.2_(HTA-2_0).CEL
## Reading in : Nicholas Bayless_US 13.1_(HTA-2_0).CEL
## Reading in : Nicholas Bayless_US 13.3_(HTA-2_0).CEL
## Reading in : Nicholas Bayless_US 14.3_(HTA-2_0).CEL
## Reading in : Nicholas Bayless_US 14.2_(HTA-2_0).CEL
## Reading in : Nicholas Bayless_US 14.1_(HTA-2_0).CEL
## Reading in : Nicholas Bayless_US 16.1_(HTA-2_0).CEL
## Reading in : Nicholas Bayless_US 17.2_(HTA-2_0).CEL
## Reading in : Nicholas Bayless_US 17.3_(HTA-2_0).CEL
## Reading in : Nicholas Bayless_US 18.3_(HTA-2_0).CEL
## Reading in : Nicholas Bayless_US 18.1_(HTA-2_0).CEL
## Reading in : Nicholas Bayless_US 19.1_(HTA-2_0).CEL
## Reading in : Nicholas Bayless_US 19.3_(HTA-2_0).CEL
## Reading in : Nicholas Bayless_US 19.2_(HTA-2_0).CEL
## Reading in : Nicholas Bayless_US 20.2_(HTA-2_0).CEL
## Reading in : Nicholas Bayless_US 21.3_(HTA-2_0).CEL
## Reading in : Nicholas Bayless_US 21.2_(HTA-2_0).CEL
## Warning in read.celfiles(sample_table$file_name, phenoData = pd, sampleNames
## = sample_table$sample_id): 'channel' automatically added to varMetadata in
## phenoData.
rawData
## HTAFeatureSet (storageMode: lockedEnvironment)
## assayData: 6892960 features, 40 samples
##   element names: exprs
## protocolData
##   rowNames: 1 2 ... 40 (40 total)
##   varLabels: exprs dates
##   varMetadata: labelDescription channel
## phenoData
##   rowNames: 1 2 ... 40 (40 total)
##   varLabels: ptidno ptb_case ... pair (7 total)
##   varMetadata: labelDescription channel
## featureData: none
## experimentData: use 'experimentData(object)'
## Annotation: pd.hta.2.0
```

4 Quality Control

MA plots on the first three samples.

```
MAplot(rawData[, 1:3], pairs=TRUE)
```

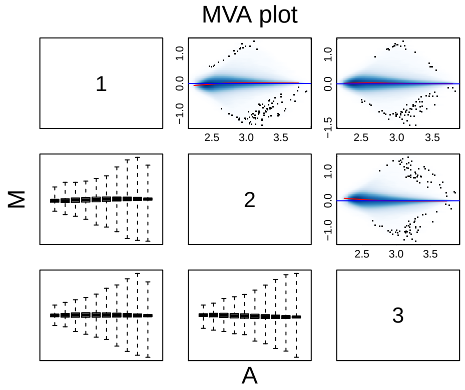

5 Preprocessing

Background subtraction, normalization and summarization using median-polish.

```
eset = rma(rawData)
## Background correcting
## Normalizing
## Calculating Expression
```

Get rid of background probes and annotate using functions in `affycoretools` package.

```
dbGetQuery(db(pd.hta.2.0), "select * from type_dict;")
##   type                                                type_id
## 1    1                                                   main
## 2    2                           Antigenomic background control
## 3    3                                 control->affx->bac_spike
## 4    4                               control->affx->polya_spike
## 5    5 ERCC (External RNA Controls Consortium) step control
## 6    6      Exonic normalization control (Positive Control)
## 7    7    Intronic normalization control (Negative Control)
## 8    8                                       Positive Control
table(getMainProbes("pd.hta.2.0")$type)
##
##     1     2     3     4     5     6     7
## 67516    23     4     4   155   698   646
eset = getMainProbes(eset)
```

Filter probes that we cannot map to symbols.

```
e2s = toTable(hta20transcriptclusterSYMBOL)
prob_ids = rownames(exprs(eset))
keep_ids = which(prob_ids %in% e2s$probe_id)
eset = ExpressionSet(assayData = exprs(eset)[keep_ids,],
                     phenoData = phenoData(eset),
                     experimentData = experimentData(eset),
                     annotation = annotation(eset))
```

Save to file.

```
class(eset)
## [1] "ExpressionSet"
## attr(,"package")
## [1] "Biobase"
show(eset)
## ExpressionSet (storageMode: lockedEnvironment)
## assayData: 29503 features, 40 samples
##   element names: exprs
## protocolData: none
## phenoData
##   sampleNames: 1 2 ... 40 (40 total)
##   varLabels: ptidno ptb_case ... pair (7 total)
##   varMetadata: labelDescription channel
## featureData: none
## experimentData: use 'experimentData(object)'
## Annotation: pd.hta.2.0
exprs(eset)[1:10, 1:2]
##                          1         2
## TC01000003.hg.1  2.124867  2.271436
## TC01000007.hg.1 11.059400 10.764831
## TC01000010.hg.1  3.202259  2.941917
## TC01000018.hg.1  6.939298  7.623561
## TC01000019.hg.1  5.839381  5.748863
## TC01000020.hg.1  5.660097  5.819165
## TC01000021.hg.1  5.654589  5.693005
## TC01000022.hg.1  5.674174  5.616228
## TC01000023.hg.1  6.026052  6.319220
## TC01000024.hg.1  5.800042  5.859162
save(eset,file = "eset.Rdata")
```

Write processed expressions to file for GEO upload.

```
geo_exprs_rma = exprs(eset)
condition = ifelse(pData(eset)$ptb_case == "control",
                   yes = "Term", no = "Preterm")
sample_name = paste0(pData(eset)$sample_id, "_",
                     condition, "_",
                     ifelse(params$treatment == "stim",
                            yes = "H1N1", "Mock"))
colnames(geo_exprs_rma) = sample_name
geo_exprs_rma %<>% as_tibble(rownames = "ID_REF")
write_csv(geo_exprs_rma, path = paste0("case_control_rma_", params$treatment, ".csv"))
```

6 Data Exploration

PCA plot of normalized expressions.

```
res_pca = prcomp(t(exprs(eset)), scale. = FALSE)
screeplot(res_pca)
```

Case-Control with Gestational Age Matching

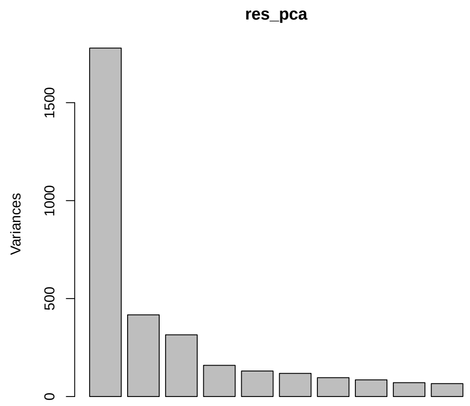


```
sample_table_annotated = eset@phenoData@data
sample_table_annotated$pair %<>% as.factor
asp_ratio = res_pca$sdev[2]^2/res_pca$sdev[1]^2
autoplot(res_pca,
         data = sample_table_annotated,
         shape = "ptb_case",
         colour = "gestage_pbmc_collected",
         size = 3,
         asp = asp_ratio)
```

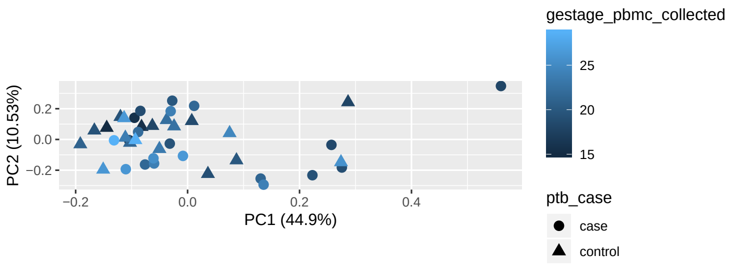


```
autoplot(res_pca,
         data = sample_table_annotated,
         shape = "ptb_case",
         colour = "gestage_delivery",
         size = 3,
         asp = asp_ratio)
```

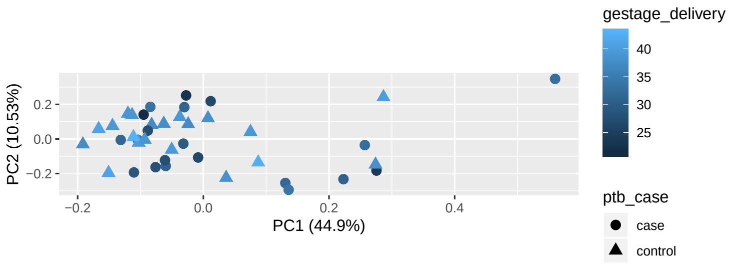


```
rownames(sample_table_annotated) = paste0(sample_table_annotated$pair,
                                          "_",
                                          sample_table_annotated$sample_id)
autoplot(res_pca,
```

```
                  data = sample_table_annotated,
                  shape = "ptb_case",
                  colour = "pair",
                  size = 3,
                  label = TRUE,
                  asp = asp_ratio)
```

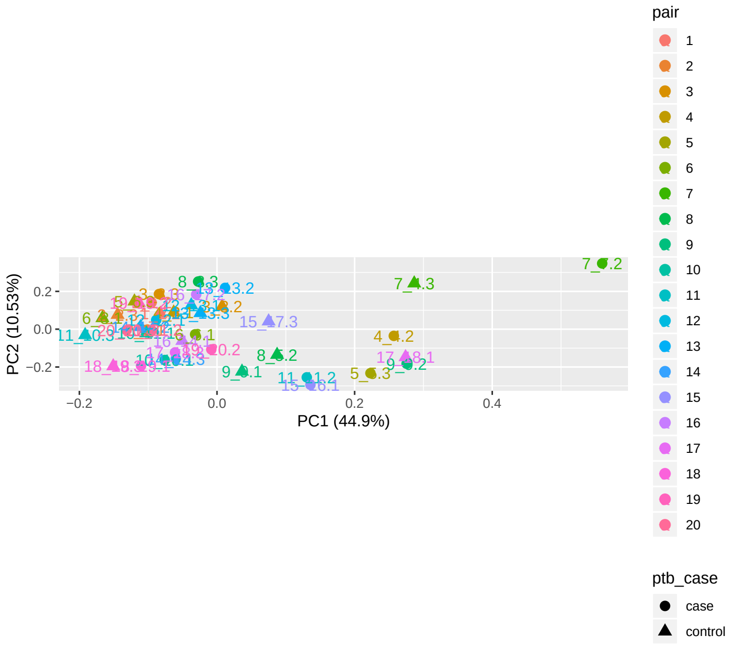

7 Differential Expression Analyses

Use `limma` for linear models to assess difference in expression. Paired analysis as described in Section 9.4.1 on page 42 in the limma vignette.

Automatic independent filtering as described in DESeq2 doc:

1. Filter genes based on mean expression
2. Fit linear model
3. Compute moderated t-tests
4. Count number of rejections at FDR of 10%

Pick the threshold that maximizes the number of discoveries.

```
mean_expr = rowMeans(exprs(eset))
thres_candidates = seq(min(mean_expr),quantile(mean_expr, probs = 0.95),1)
fit_list = lapply(thres_candidates, function(thres) {
  cat("Automatic independent filtering: thres = ", thres,"\n")
  # threshold
  eset_thres = ExpressionSet(assayData = exprs(eset)[mean_expr >= thres,],
                             phenoData = phenoData(eset),
                             experimentData = experimentData(eset),
                             annotation = annotation(eset))
```

```
  # fit model
  targets = eset@phenoData@data
  pair = factor(targets$pair)
  treat = factor(targets$ptb_case, levels = c("control","case"))
  design = model.matrix(~ pair + treat)
  fit = lmFit(eset_thres, design)
  eBayes(fit)
})
## Automatic independent filtering: thres =  1.415861
## Automatic independent filtering: thres =  2.415861
## Automatic independent filtering: thres =  3.415861
## Automatic independent filtering: thres =  4.415861
## Automatic independent filtering: thres =  5.415861
## Automatic independent filtering: thres =  6.415861
num_sig = sapply(fit_list, function(fit) {
  gene_table = topTable(fit, coef = "treatcase", adjust = "BH",
                        number = nrow(fit))
  gene_table %<>% dplyr::filter(adj.P.Val < 0.1)
  nrow(gene_table)
})
num_sig
## [1] 0 0 0 0 0 0
fit = fit_list[[which.max(num_sig)]]
```

The `topTable` command provides us a way of ranking genes for further evaluation. In the case below, we adjust for multiple testing by FDR.

```
gene_table = topTable(fit, coef = "treatcase", adjust = "BH",
                      number = nrow(fit))
hist(gene_table$P.Value,breaks = 20)
```

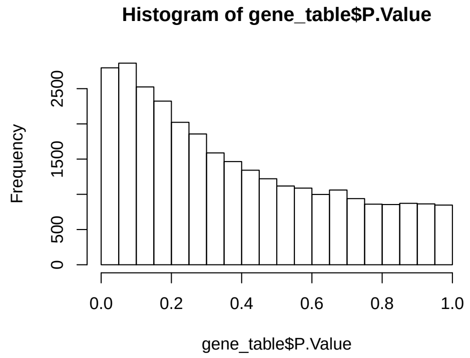


```
sum(gene_table$adj.P.Val < 0.1)
## [1] 0
```

Volcano plots for quality control.

```r
volcanoplot(fit, coef = "treatcase", highlight = 10)
```

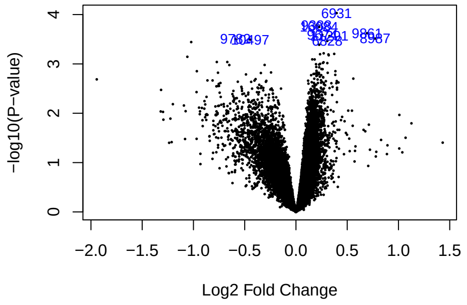

Map between manufacturer identifiers and gene symbols.

```r
map_gene_symbol = function(gene_table) {
  prob_ids = rownames(gene_table)
  symbol = sapply(prob_ids,function(prob_id) {
    matching_symbol = e2s$symbol[prob_id==e2s$probe_id]
    if(length(matching_symbol)==0) matching_symbol = "No_Symbol_Found"
    matching_symbol
  }) %>% unlist
  gene_table = cbind(gene_table,symbol=symbol,stringsAsFactors=FALSE)
  gene_table
}
gene_table = map_gene_symbol(gene_table)
print(head(gene_table, n = 10))
##                        logFC  AveExpr         t      P.Value adj.P.Val
## TC04001942.hg.1  0.3964871 3.316441  4.736341 9.335941e-05 0.5114967
## TC06002060.hg.1  0.2001396 2.642368  4.506959 1.646325e-04 0.5114967
## TC11000211.hg.1  0.2261746 5.190697  4.475880 1.777978e-04 0.5114967
## TC06003369.hg.1  0.6938335 6.947984  4.356977 2.386576e-04 0.5114967
## TC06002625.hg.1  0.2560004 3.762744  4.326441 2.573996e-04 0.5114967
## TC07002768.hg.1  0.3270357 5.441849  4.302524 2.730997e-04 0.5114967
## TC06001226.hg.1  0.7722665 6.874004  4.255594 3.067389e-04 0.5114967
## TC06003141.hg.1 -0.5904733 4.303887 -4.246605 3.136386e-04 0.5114967
## TC07000793.hg.1 -0.4470670 6.128909 -4.235625 3.222778e-04 0.5114967
## TC04001652.hg.1  0.3054274 4.363445  4.212081 3.416088e-04 0.5114967
##                          B      symbol
## TC04001942.hg.1 -0.2228733 SEPSECS-AS1
## TC06002060.hg.1 -0.5255553     FAM184A
## TC11000211.hg.1 -0.5670429       SPON1
## TC06003369.hg.1 -0.7267408      TUBB2B
## TC06002625.hg.1 -0.7679904     ALDH5A1
## TC07002768.hg.1 -0.8003627     ZNF890P
## TC06001226.hg.1 -0.8640440      TUBB2A
## TC06003141.hg.1 -0.8762644       TIAM2
## TC07000793.hg.1 -0.8912032     TSPAN33
## TC04001652.hg.1 -0.9232694   LOC729870
```

Write to text file.

```r
file_name_processed = paste0("case_control_", params$treatment, ".csv")
file_name_processed
## [1] "case_control_unstim.csv"
write_csv(gene_table, path = file_name_processed)
```

Add gene names to standard volcano plot.

```r
logfc_thres = 1 # logFC threshold
pvalue_thres = 3.5 # -log10 of unadjusted p-value
gene_table %<>% mutate(
  show = ifelse(abs(logFC) > logfc_thres | pvalue_thres < -log10(P.Value),
                "yes", "no")
)
gvolcano = ggplot(gene_table, aes(logFC, -log10(P.Value), color = show)) +
  geom_point() +
  geom_vline(xintercept = c(-logfc_thres,logfc_thres), linetype = 2) +
  geom_hline(yintercept = pvalue_thres, linetype = 2) +
  geom_text_repel(
    data = dplyr::filter(gene_table, show == "yes"),
    aes(label = symbol),
    size = 5,
    box.padding = unit(0.35, "lines"),
    point.padding = unit(0.3, "lines")
  ) +
  xlab("log2 fold change (up-regulated in preterm -->)") +
  ylab("-log10 p-value") +
  theme(legend.position = "none") +
  scale_colour_manual(values = c("gray", "black")) +
  ggtitle(ifelse(params$treatment == "unstim",
                 "Unstimulated Samples",
                 "Stimulated Samples"))
gvolcano
```

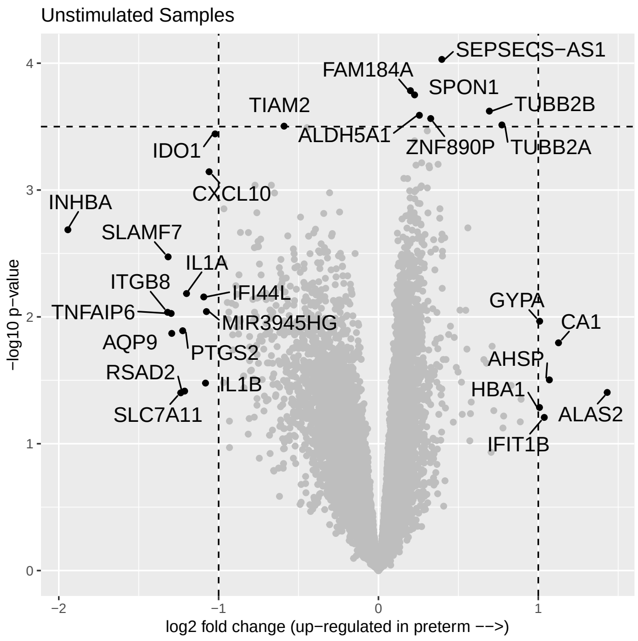


```r
save(gvolcano, file = paste0("gvolcano_",params$treatment,".Rdata"))
```

8 Patient Variability

Visualize the pair-to-pair variability.

```r
# prepare tables
targets = eset@phenoData@data
targets %<>% mutate(id = 1:nrow(targets))
GA_affy = read_csv("GA_affy.csv")
## Parsed with column specification:
## cols(
##   `Sample name` = col_character(),
##   title = col_character(),
##   `CEL file` = col_character(),
##   `source name` = col_character(),
##   organism = col_character(),
##   `characteristics: condition` = col_character(),
##   `characteristics: treatment` = col_character(),
##   `characteristics: rin` = col_double(),
##   `characteristics: run_day` = col_datetime(format = ""),
##   `characteristics: run_batch` = col_double(),
##   `characteristics: viable_cell_count` = col_double(),
##   `characteristics: viability` = col_double(),
##   `characteristics: ptidno` = col_double(),
##   `characteristics: gestage_delivery` = col_double(),
##   `characteristics: gestage_enroll` = col_double(),
##   molecule = col_character(),
##   label = col_character(),
##   description = col_logical(),
```

```
##   `chip name or GEO platform id` = col_character()
## )
GA_affy %<>% dplyr::filter(
  `characteristics: treatment` ==
    ifelse(params$treatment == "stim", yes = "H1N1", "Mock")
  )
GA_affy %<>% dplyr::rename(ptidno = `characteristics: ptidno`)
targets %<>% left_join(GA_affy, by = "ptidno")
tb_case = targets[targets$ptb_case == "case", ]
tb_control = targets[targets$ptb_case == "control", ]
tb = left_join(tb_control, tb_case, by = "pair",
               suffix = c(".control", ".case"))

# take diff within pairs
tb_exrs = exprs(eset)
X = tb_exrs[ ,tb$id.control]
Y = tb_exrs[ ,tb$id.case]
D = Y - X

# implement my own paired t-test
t_stat = apply(D, MARGIN = 1,
               function(x) t.test(x)$statistic)
hist(t_stat, breaks = 100)
```

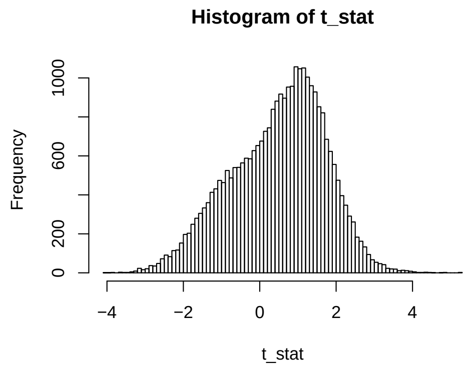


```
# plot
tb_D = D %>% as_tibble %>% mutate(gene_id = 1:nrow(D))
tb_D %<>% gather(pair, value, -gene_id)
tb_D$pair %<>% factor(levels = sort(unique(as.integer(tb_D$pair))))
ggplot(tb_D, aes(value, group = pair)) +
  geom_density() +
  xlim(c(-2, 2))
## Warning: Removed 2926 rows containing non-finite values (stat_density).
```

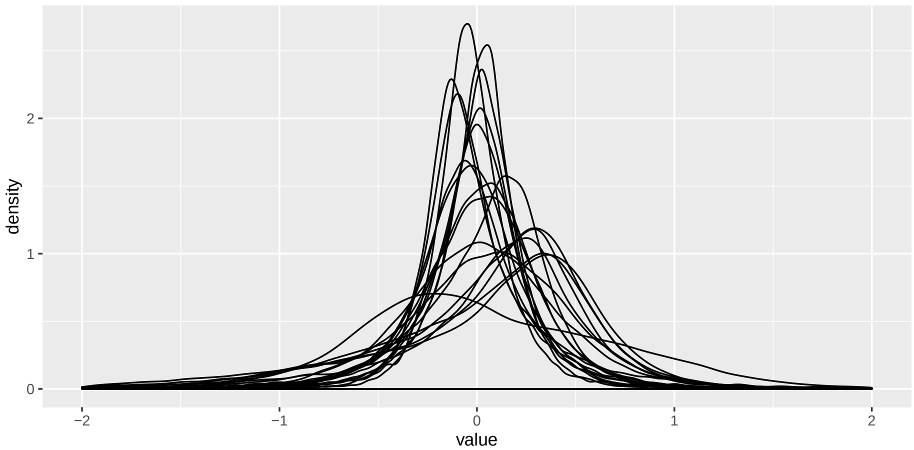


```
ggplot(tb_D, aes(value)) +
  geom_density() +
  facet_wrap(~pair) +
  xlim(c(-2, 2))
## Warning: Removed 2926 rows containing non-finite values (stat_density).
```

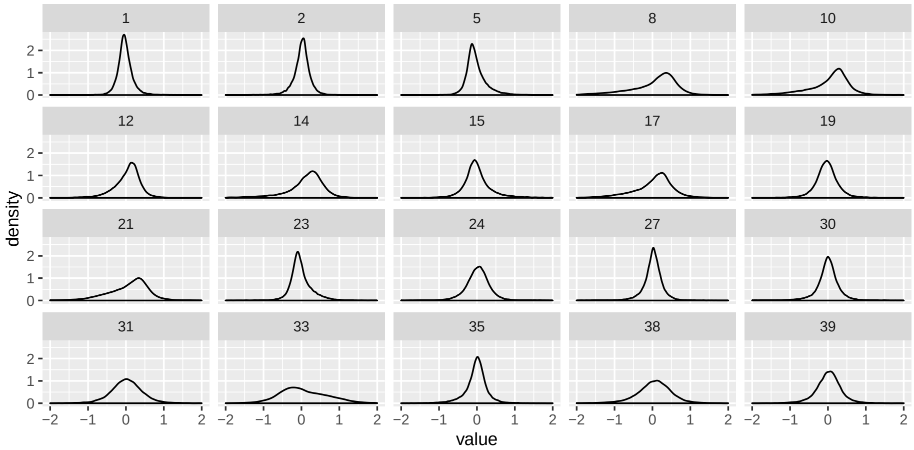

The variability does not seem to be related to `run_day` and `run_batch`.

```
table(targets$pair, targets$`characteristics: run_day`)
##
##      2019-02-11 2019-02-19 2019-02-24 2019-02-26 2019-03-01 2019-03-07
##   1           2          0          0          0          0          0
##   2           2          0          0          0          0          0
##   3           2          0          0          0          0          0
##   4           2          0          0          0          0          0
##   5           2          0          0          0          0          0
##   6           1          1          0          0          0          0
##   7           1          1          0          0          0          0
##   8           1          1          0          0          0          0
##   9           0          2          0          0          0          0
##   10          0          1          1          0          0          0
##   11          0          1          1          0          0          0
##   12          0          0          1          1          0          0
```

```
##   13          0          0          0          2          0          0
##   14          0          0          0          2          0          0
##   15          0          0          0          0          2          0
##   16          0          0          0          1          1          0
##   17          0          0          0          0          0          2
##   18          0          0          0          0          0          2
##   19          0          0          0          0          0          2
##   20          0          0          0          0          0          2
table(targets$pair, targets$`characteristics: run_batch`)
##
##      1 2 3
##   1  2 0 0
##   2  2 0 0
##   3  0 2 0
##   4  0 2 0
##   5  0 0 2
##   6  1 0 1
##   7  1 1 0
##   8  1 0 1
##   9  0 2 0
##   10 1 1 0
##   11 1 1 0
##   12 2 0 0
##   13 2 0 0
##   14 2 0 0
##   15 2 0 0
##   16 2 0 0
##   17 2 0 0
##   18 2 0 0
##   19 1 1 0
##   20 0 2 0
```

9 Power Analysis

Power analysis using local FDR methodology.

```
# fit model
targets = eset@phenoData@data
pair = factor(targets$pair)
treat = factor(targets$ptb_case, levels = c("control","case"))
design = model.matrix(~ treat + pair)
fit = lmFit(eset, design)

# ordinary t-statistic
t_stat = fit$coef[, "treatcase"] / fit$stdev.unscaled[, "treatcase"] / fit$sigma
z_stat = qnorm(pt(t_stat, df = ncol(exprs(eset))-2))
hist(t_stat, breaks = 100)
```

Case-Control with Gestational Age Matching

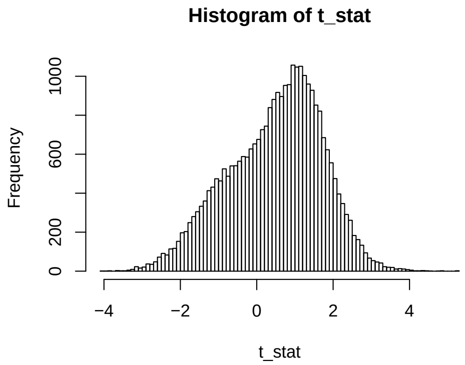


```r
hist(z_stat, breaks = 100)
```

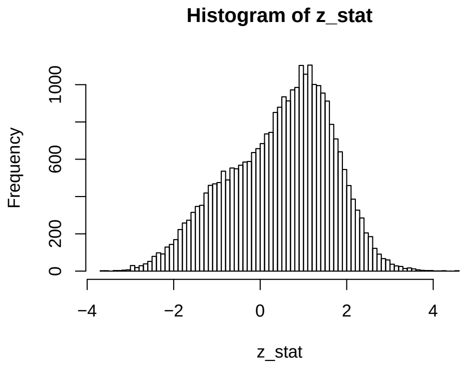


```r
locfdr_res = locfdr(z_stat, df = 7, plot = 4)
```

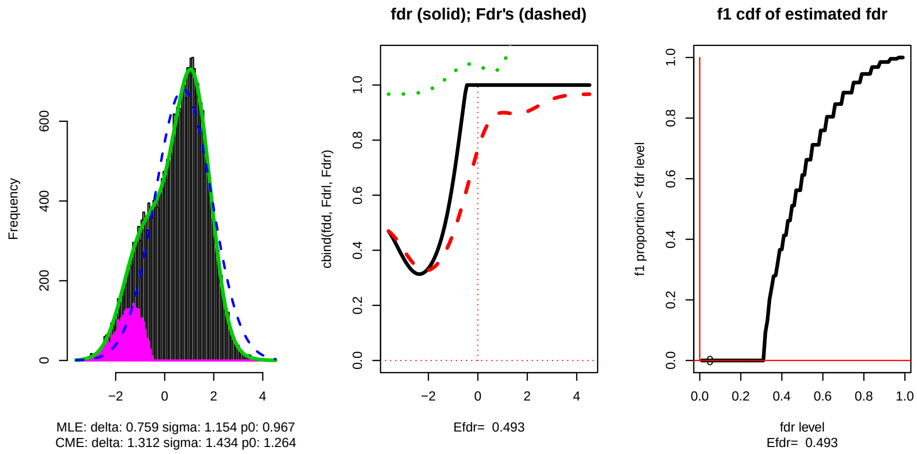


```r
locfdr_res$Efdr
##      Efdr     Eleft    Eright  Efdrtheo    Eleft0   Eright0
## 0.4926096 0.4926096 1.0000000 0.3135995 0.8643666 0.2658268
locfdr_res = locfdr(z_stat, df = 7, plot = 1)
```

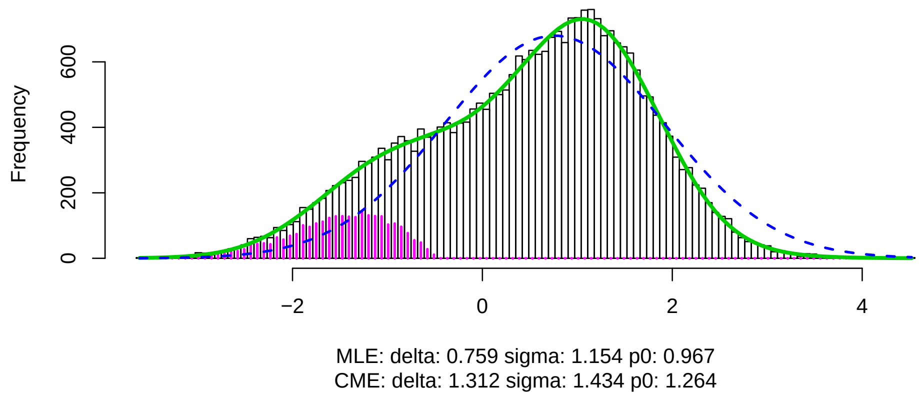

Large values Efdr > 0.4 indicate low power (according to Seciton 3 of “Size, power and false discovery rates”, Efron 2007).

10 Gene Set Enrichment Analysis

Standard KEGG analysis.

```
fit %<>% eBayes
pathway_results = kegga(fit, species.KEGG = "hsa")
## No DE genes
topKEGG(pathway_results)
## data frame with 0 columns and 0 rows
```

Session Info

```
sessionInfo()
## R version 3.5.1 (2018-07-02)
## Platform: x86_64-apple-darwin15.6.0 (64-bit)
## Running under: macOS  10.15.3
##
## Matrix products: default
## BLAS: /Library/Frameworks/R.framework/Versions/3.5/Resources/lib/libRblas.0.dylib
## LAPACK: /Library/Frameworks/R.framework/Versions/3.5/Resources/lib/libRlapack.dylib
##
## locale:
## [1] en_US.UTF-8/en_US.UTF-8/en_US.UTF-8/C/en_US.UTF-8/en_US.UTF-8
##
## attached base packages:
## [1] stats4    parallel  stats     graphics  grDevices utils     datasets
## [8] methods   base
##
## other attached packages:
##  [1] pd.hta.2.0_3.12.2               DBI_1.0.0
##  [3] RSQLite_2.1.1                   locfdr_1.1-8
```

```
##  [5] tidyr_1.0.0                     ggrepel_0.8.1
##  [7] tibble_2.1.3                    stringr_1.4.0
##  [9] readxl_1.3.1                    dplyr_0.8.3
## [11] readr_1.3.1                     statmod_1.4.32
## [13] magrittr_1.5                    ggfortify_0.4.7
## [15] ggplot2_3.2.1                   MatchIt_3.0.2
## [17] genefilter_1.64.0               affycoretools_1.54.0
## [19] hta20transcriptcluster.db_8.7.0 org.Hs.eg.db_3.7.0
## [21] AnnotationDbi_1.44.0            limma_3.38.3
## [23] oligo_1.46.0                    Biostrings_2.50.2
## [25] XVector_0.22.0                  IRanges_2.16.0
## [27] S4Vectors_0.20.1                Biobase_2.42.0
## [29] oligoClasses_1.44.0             BiocGenerics_0.28.0
## [31] BiocStyle_2.10.0
##
## loaded via a namespace (and not attached):
##   [1] utf8_1.1.4                  R.utils_2.8.0
##   [3] tidyselect_0.2.5            htmlwidgets_1.3
##   [5] grid_3.5.1                  BiocParallel_1.16.6
##   [7] munsell_0.5.0               codetools_0.2-16
##   [9] preprocessCore_1.44.0       withr_2.1.2
##  [11] colorspace_1.4-1            Category_2.48.1
##  [13] OrganismDbi_1.24.0          knitr_1.22
##  [15] rstudioapi_0.10             labeling_0.3
##  [17] GenomeInfoDbData_1.2.0      hwriter_1.3.2
##  [19] bit64_0.9-7                 farver_2.0.1
##  [21] vctrs_0.2.1                 xfun_0.6
##  [23] biovizBase_1.30.1           affxparser_1.54.0
##  [25] R6_2.4.1                    GenomeInfoDb_1.18.2
##  [27] optmatch_0.9-11             locfit_1.5-9.1
##  [29] AnnotationFilter_1.6.0      bitops_1.0-6
##  [31] reshape_0.8.8               DelayedArray_0.8.0
##  [33] assertthat_0.2.1            scales_1.1.0
##  [35] nnet_7.3-12                 gtable_0.3.0
##  [37] affy_1.60.0                 ggbio_1.30.0
##  [39] svd_0.5                     ensembldb_2.6.8
##  [41] rlang_0.4.2                 zeallot_0.1.0
##  [43] splines_3.5.1               rtracklayer_1.42.2
##  [45] lazyeval_0.2.2              acepack_1.4.1
##  [47] dichromat_2.0-0             checkmate_1.9.4
##  [49] BiocManager_1.30.4          yaml_2.2.0
##  [51] reshape2_1.4.3              abind_1.4-5
##  [53] GenomicFeatures_1.34.8      backports_1.1.5
##  [55] Hmisc_4.2-0                 RBGL_1.58.2
##  [57] tools_3.5.1                 bookdown_0.9
##  [59] ellipsis_0.3.0              affyio_1.52.0
##  [61] gplots_3.0.1.1              ff_2.2-14
##  [63] RColorBrewer_1.1-2          Rcpp_1.0.3
##  [65] plyr_1.8.5                  base64enc_0.1-3
##  [67] progress_1.2.2              zlibbioc_1.28.0
##  [69] purrr_0.3.3                 RCurl_1.95-4.12
```

```
##  [71] prettyunits_1.1.0           rpart_4.1-15
##  [73] SummarizedExperiment_1.12.0 cluster_2.0.9
##  [75] data.table_1.12.6           SparseM_1.77
##  [77] ProtGenerics_1.14.0         matrixStats_0.55.0
##  [79] hms_0.5.2                   evaluate_0.13
##  [81] xtable_1.8-4                XML_3.98-1.19
##  [83] gcrma_2.54.0                gridExtra_2.3
##  [85] RItools_0.1-17              compiler_3.5.1
##  [87] biomaRt_2.38.0              KernSmooth_2.23-15
##  [89] crayon_1.3.4                ReportingTools_2.22.1
##  [91] R.oo_1.22.0                 htmltools_0.3.6
##  [93] GOstats_2.48.0              Formula_1.2-3
##  [95] geneplotter_1.60.0          MASS_7.3-51.4
##  [97] Matrix_1.2-17               cli_2.0.1
##  [99] R.methodsS3_1.7.1           gdata_2.18.0
## [101] GenomicRanges_1.34.0        pkgconfig_2.0.3
## [103] GenomicAlignments_1.18.1    foreign_0.8-71
## [105] foreach_1.4.4               annotate_1.60.1
## [107] AnnotationForge_1.24.0      VariantAnnotation_1.28.13
## [109] digest_0.6.23               graph_1.60.0
## [111] rmarkdown_1.12              cellranger_1.1.0
## [113] htmlTable_1.13.2            edgeR_3.24.3
## [115] GSEABase_1.44.0             curl_4.3
## [117] Rsamtools_1.34.1            gtools_3.8.1
## [119] lifecycle_0.1.0             PFAM.db_3.7.0
## [121] fansi_0.4.1                 BSgenome_1.50.0
## [123] pillar_1.4.3                lattice_0.20-38
## [125] GGally_1.4.0                httr_1.4.0
## [127] survival_2.44-1.1           GO.db_3.7.0
## [129] glue_1.3.1                  iterators_1.0.10
## [131] bit_1.1-14                  Rgraphviz_2.26.0
## [133] stringi_1.4.5               blob_1.1.1
## [135] DESeq2_1.22.2               latticeExtra_0.6-28
## [137] caTools_1.17.1.2            memoise_1.1.0
```
