# Supplementary material for: Influenza-Induced Interferon Lambda Response Is Associated With Longer Time to Delivery Among Pregnant Kenyan Women
Source: Front Immunol. 2020 Mar 17;11:452. doi: 10.3389/fimmu.2020.00452 (PMC7089959; doi:10.3389/fimmu.2020.00452)
Supplement: Supplementary file 2 [file Presentation_1.zip › TimeToDelivery_Microarray_H1N1.pdf]

Separate Mock/H1N1 Time to Delivery Analysis on Microarray Data

Christof Seiler

3 February 2020

Contents

1 Goal 2
2 Prerequisites 2
3 Import Data 3
4 Preprocessing 5
5 Data Exploration 5
6 Differential Expression Analyses 7
7 Network Analysis 11
7.1 Download Gene Network 11
7.2 Find Subgraph 12
7.3 Visualize Network 13
7.4 Single Genes Expression 15
8 Pathways Analysis 16
9 Time to Delivery Prediction 18
Session Info 19

1 Goal

- Load and normalize data using `oligo`
- Differential analysis using `limma`
- Network analysis using `BioNet`
- Pathway analysis using `KEGGREST`

2 Prerequisites

Install necessary packages from bioconductor repository. Run this code only once to install packages.

```
pkgs_needed = c("oligo","limma","affycoretools","genefilter","glmnet",
                "hta20transcriptcluster.db","ggfortify","magrittr",
                "statmod","stringr","tibble","dplyr",
                "STRINGdb","BioNet","DLBCL","org.Hs.eg.db","KEGGREST",
                "igraph","intergraph","ggnetwork","ggthemes","readr")
letsinstall = setdiff(pkgs_needed, installed.packages())
if (length(letsinstall) > 0) {
  source("http://bioconductor.org/biocLite.R")
  biocLite(letsinstall)
}
```

Load packages.

```
library("oligo")
library("limma")
library("affycoretools")
library("genefilter")
library("glmnet")
library("hta20transcriptcluster.db")
library("ggfortify")
library("magrittr")
library("statmod")
library("stringr")
library("tibble")
library("dplyr")
library("STRINGdb")
library("BioNet")
library("DLBCL")
library("org.Hs.eg.db")
library("KEGGREST")
library("igraph")
library("intergraph")
library("ggnetwork")
library("ggthemes")
library("sna")
library("statnet.common")
library("network")
library("readr")
theme_set(theme_few())
```

```
scale_colour_discrete = function(...) scale_colour_few()
```

3 Import Data

Then load Affymetrix CEL files. At this stage, Bioconductor will automatically download the necessary annotation packages and install them for us. Add time to delivery variable.

```
sample_table = read.csv("sample_table.csv")
cel_filenames = paste0(as.character(sample_table$array_name),".CEL")
sample_table %<>% mutate(
  cel_filename = cel_filenames,
  sample_name = paste(sample_table$sample_id,
                      sample_table$condition,
                      sample_table$treatment,sep = "_")
  )
write_csv(sample_table, path = "sample_table_cel.csv")
params$treatment
## [1] "H1N1"
sample_table %<>% filter(treatment == params$treatment)
sample_table$time_to_delivery = sample_table$gestage_enroll -
  sample_table$gestage_delivery
pd = as(sample_table, "AnnotatedDataFrame")
cel_filenames = paste0(as.character(sample_table$array_name),".CEL")
rawData = read.celfiles(cel_filenames, phenoData = pd,
                        sampleNames = sample_table$sample_name)
## Loading required package: pd.hta.2.0
## Loading required package: RSQLite
## Loading required package: DBI
## Platform design info loaded.
## Reading in : Nicholas Bayless_H1N1 1.1_(HTA-2_0).CEL
## Reading in : Nicholas Bayless_H1N1 1.2_(HTA-2_0).CEL
## Reading in : Nicholas Bayless_H1N1 1.3_(HTA-2_0).CEL
## Reading in : Nicholas Bayless_H1N1 2.1_(HTA-2_0).CEL
## Reading in : Nicholas Bayless_H1N1 2.2_(HTA-2_0).CEL
## Reading in : Nicholas Bayless_H1N1 2.3_(HTA-2_0).CEL
## Reading in : Nicholas Bayless_H1N1 3.2_(HTA-2_0).CEL
## Reading in : Nicholas Bayless_H1N1 3.3_(HTA-2_0).CEL
## Reading in : Nicholas Bayless_H1N1 4.3_(HTA-2_0).CEL
## Reading in : Nicholas Bayless_H1N1 5.1_(HTA-2_0).CEL
## Reading in : Nicholas Bayless_H1N1 5.3_(HTA-2_0).CEL
## Reading in : Nicholas Bayless_H1N1 6.1_(HTA-2_0).CEL
## Reading in : Nicholas Bayless_H1N1 6.2_(HTA-2_0).CEL
## Reading in : Nicholas Bayless_H1N1 6.3_(HTA-2_0).CEL
## Reading in : Nicholas Bayless_7.1 +_(HTA-2_0).CEL
## Reading in : Nicholas Bayless_7.2 +_(HTA-2_0).CEL
## Reading in : Nicholas Bayless_7.3 +_(HTA-2_0).CEL
## Reading in : Nicholas Bayless_8.1 +_(HTA-2_0).CEL
## Reading in : Nicholas Bayless_8.2 +_(HTA-2_0).CEL
## Reading in : Nicholas Bayless_8.3 +_(HTA-2_0).CEL
## Reading in : Nicholas Bayless_9.1 +_(HTA-2_0).CEL
```

```
## Reading in : Nicholas Bayless_9.2 +_(HTA-2_0).CEL
## Reading in : Nicholas Bayless_9.3 +_(HTA-2_0).CEL
## Reading in : Nicholas Bayless_10.1 +_(HTA-2_0).CEL
## Reading in : Nicholas Bayless_10.2 +_(HTA-2_0).CEL
## Reading in : Nicholas Bayless_10.3 +_(HTA-2_0).CEL
## Reading in : Nicholas Bayless_11.1 +_(HTA-2_0).CEL
## Reading in : Nicholas Bayless_11.2 +_(HTA-2_0).CEL
## Reading in : Nicholas Bayless_11.3 +_(HTA-2_0).CEL
## Reading in : Nicholas Bayless_12.1 +_(HTA-2_0).CEL
## Reading in : Nicholas Bayless_12.2 +_(HTA-2_0).CEL
## Reading in : Nicholas Bayless_H1N1 13.1_(HTA-2_0).CEL
## Reading in : Nicholas Bayless_H1N1 13.2_(HTA-2_0).CEL
## Reading in : Nicholas Bayless_H1N1 13.3_(HTA-2_0).CEL
## Reading in : Nicholas Bayless_H1N1 14.1_(HTA-2_0).CEL
## Reading in : Nicholas Bayless_H1N1 14.2_(HTA-2_0).CEL
## Reading in : Nicholas Bayless_H1N1 14.3_(HTA-2_0).CEL
## Reading in : Nicholas Bayless_H1N1 16.1_(HTA-2_0).CEL
## Reading in : Nicholas Bayless_H1N1 16.2_(HTA-2_0).CEL
## Reading in : Nicholas Bayless_H1N1 16.3_(HTA-2_0).CEL
## Reading in : Nicholas Bayless_H1N1 17.1_(HTA-2_0).CEL
## Reading in : Nicholas Bayless_H1N1 17.2_(HTA-2_0).CEL
## Reading in : Nicholas Bayless_H1N1 17.3_(HTA-2_0).CEL
## Reading in : Nicholas Bayless_H1N1 18.1_(HTA-2_0).CEL
## Reading in : Nicholas Bayless_H1N1 18.3_(HTA-2_0).CEL
## Reading in : Nicholas Bayless_H1N1 19.1_(HTA-2_0).CEL
## Reading in : Nicholas Bayless_H1N1 19.2_(HTA-2_0).CEL
## Reading in : Nicholas Bayless_H1N1 19.3_(HTA-2_0).CEL
## Reading in : Nicholas Bayless_H1N1 20.1_(HTA-2_0).CEL
## Reading in : Nicholas Bayless_H1N1 20.2_(HTA-2_0).CEL
## Reading in : Nicholas Bayless_H1N1 20.3_(HTA-2_0).CEL
## Reading in : Nicholas Bayless_H1N1 21.1_(HTA-2_0).CEL
## Reading in : Nicholas Bayless_H1N1 21.2_(HTA-2_0).CEL
## Reading in : Nicholas Bayless_H1N1 21.3_(HTA-2_0).CEL
## Warning in read.celfiles(cel_filenames, phenoData = pd, sampleNames =
## sample_table$sample_name): 'channel' automatically added to varMetadata in
## phenoData.
rawData
## HTAFeatureSet (storageMode: lockedEnvironment)
## assayData: 6892960 features, 54 samples
##   element names: exprs
## protocolData
##   rowNames: 1 2 ... 54 (54 total)
##   varLabels: exprs dates
##   varMetadata: labelDescription channel
## phenoData
##   rowNames: 1 2 ... 54 (54 total)
##   varLabels: X sample_id ... time_to_delivery (16 total)
##   varMetadata: labelDescription channel
## featureData: none
## experimentData: use 'experimentData(object)'
## Annotation: pd.hta.2.0
```

4 Preprocessing

Background subtraction, normalization and summarization using median-polish.

```
eset = rma(rawData)
## Background correcting
## Normalizing
## Calculating Expression
```

Get rid of background probes and annotate using functions in `affycoretools` package.

```
dbGetQuery(db(pd.hta.2.0), "select * from type_dict;")
##   type                                        type_id
## 1    1                                           main
## 2    2                  Antigenomic background control
## 3    3                        control->affx->bac_spike
## 4    4                      control->affx->polya_spike
## 5    5 ERCC (External RNA Controls Consortium) step control
## 6    6      Exonic normalization control (Positive Control)
## 7    7    Intronic normalization control (Negative Control)
## 8    8                               Positive Control
table(getMainProbes("pd.hta.2.0")$type)
##
##     1     2     3     4     5     6     7
## 67516    23     4     4   155   698   646
eset = getMainProbes(eset)
```

Filter probes that we cannot map to symbols.

```
e2s = toTable(hta20transcriptclusterSYMBOL)
prob_ids = rownames(exprs(eset))
keep_ids = which(prob_ids %in% e2s$probe_id)
eset = ExpressionSet(assayData = exprs(eset)[keep_ids,],
                     phenoData = phenoData(eset),
                     experimentData = experimentData(eset),
                     annotation = annotation(eset))
```

Write processed expressions to file for GEO upload.

```
geo_exprs_rma = exprs(eset)
colnames(geo_exprs_rma) = pData(eset)$sample_name
geo_exprs_rma %<>% as_tibble(rownames = "ID_REF")
write_csv(geo_exprs_rma, path = paste0("time_to_delivery_rma_", params$treatment, ".csv"))
```

5 Data Exploration

PCA plot of normalized expressions.

```
res_pca = prcomp(t(exprs(eset)),scale. = FALSE)
screeplot(res_pca)
```

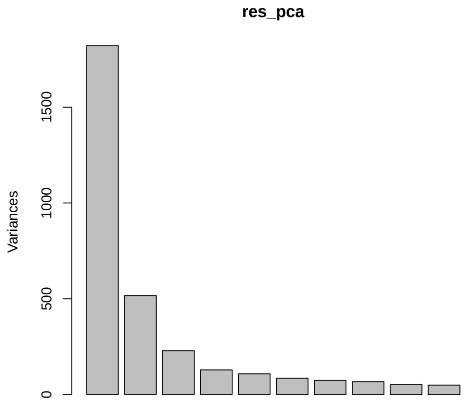


```r
sample_table = eset@phenoData@data
asp_ratio = res_pca$sdev[2]^2/res_pca$sdev[1]^2
autoplot(res_pca, data = sample_table, colour = 'treatment',
         shape = 'condition', size = 3, asp = asp_ratio)
```

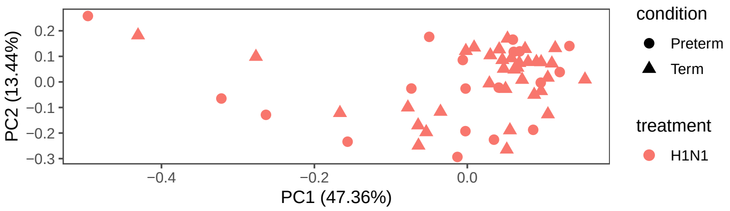


```r
rownames(sample_table) = paste(sample_table$sample_id,
                               sample_table$treatment,sep = "_")
autoplot(res_pca, data = sample_table,
         shape = FALSE, label = TRUE, label.size = 3, asp = asp_ratio)
```

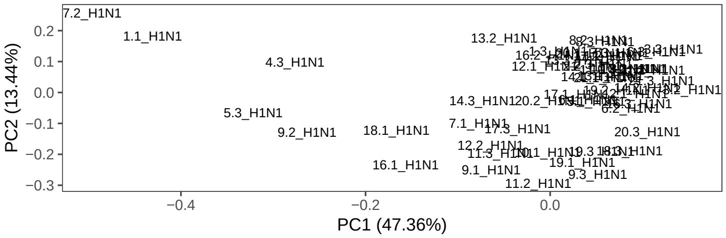

Check for batch effects.

```r
sample_table$run_batch %<>% as.factor()
sample_table$run_day %<>% factor(levels(sample_table$run_day)[c(2:5,1,6)])
autoplot(res_pca, data = sample_table, colour = 'run_day',
         shape = 'condition', size = 3, asp = asp_ratio)
```

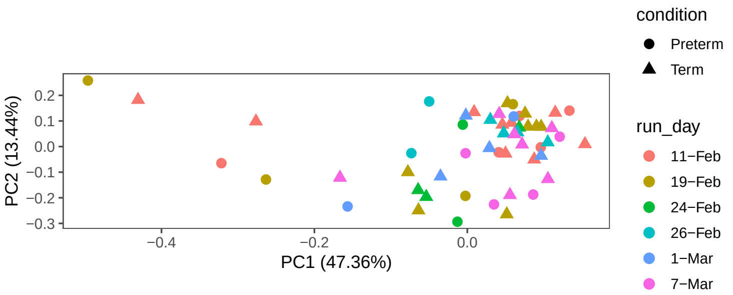


```
autoplot(res_pca, data = sample_table, colour = 'run_batch',
         shape = 'condition', size = 3, asp = asp_ratio)
```

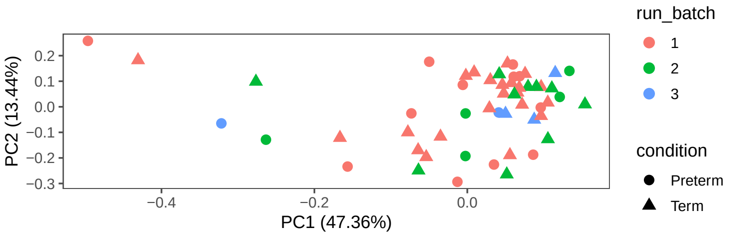

6 Differential Expression Analyses

Use `limma` for linear models to assess difference in expression. Define design matrix.

```
design = model.matrix(~ time_to_delivery + gestage_delivery, sample_table)
colnames(design) = str_replace(string = colnames(design),
                               pattern = ":",replacement = "_") %>%
  str_replace(string = .,pattern = "treatment",replacement = "")
colnames(design)
## [1] "(Intercept)"      "time_to_delivery" "gestage_delivery"
```

Choose mean expression threshold that maximizes number of differentially expressed genes. This approach is called automatic independent filtering and used in DESeq2.

```
mean_expr = rowMeans(exprs(eset))
thres_candidates = seq(min(mean_expr),quantile(mean_expr, probs = 0.95),1)
fit_list = lapply(thres_candidates, function(thres) {
  cat("Automatic independent filtering: thres = ", thres,"\n")
  # threshold
  eset_thres = ExpressionSet(assayData = exprs(eset)[mean_expr >= thres,],
                             phenoData = phenoData(eset),
                             experimentData = experimentData(eset),
                             annotation = annotation(eset))
  # fit model
  fit = lmFit(eset_thres, design)
  eBayes(fit)
})
## Automatic independent filtering: thres =  1.349877
## Automatic independent filtering: thres =  2.349877
## Automatic independent filtering: thres =  3.349877
```

```
## Automatic independent filtering: thres =  4.349877
## Automatic independent filtering: thres =  5.349877
## Automatic independent filtering: thres =  6.349877
num_sig = sapply(fit_list, function(fit) {
  coeffs = colnames(design)[-1]
  gene_table_combined = lapply(coeffs,function(coeff_name) {
    gene_table = topTable(fit, coef = coeff_name, adjust = "BH",
                          number = nrow(fit))
    gene_table %>% dplyr::filter(adj.P.Val < 0.1) %>%
      add_column(coeff = coeff_name)
  }) %>% bind_rows()
  nrow(gene_table_combined)
})
num_sig
## [1]  48  80 181 168   9   0
fit = fit_list[[which.max(num_sig)]]
```

Save results in a list of tables.

```
coeffs = colnames(design)[-1]
gene_table_list = lapply(coeffs, function(coeff_name) {
  gene_table = topTable(fit,
                        coef = coeff_name,
                        adjust = "BH",
                        number = nrow(fit))
  hist(gene_table$P.Value,breaks = 100,main = coeff_name)
  gene_table
})
```

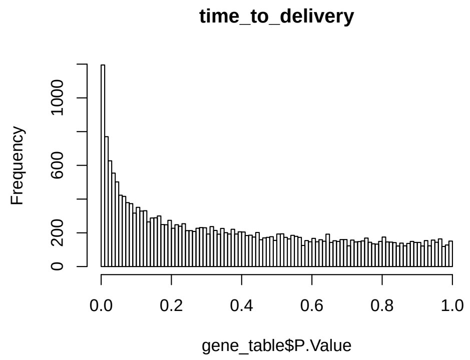

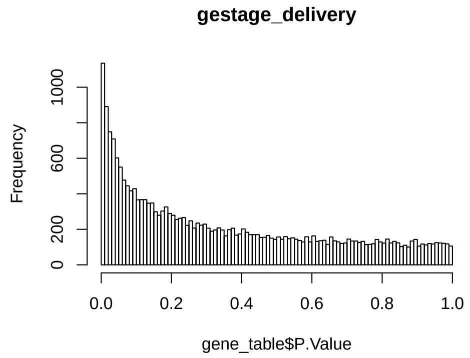

```
names(gene_table_list) = coeffs
```

Volcano plots for quality control.

```
for(coeff_name in coeffs) {
  cat(coeff_name,"\n")
  volcanoplot(fit,coef = coeff_name,highlight = 10)
}
## time_to_delivery
```

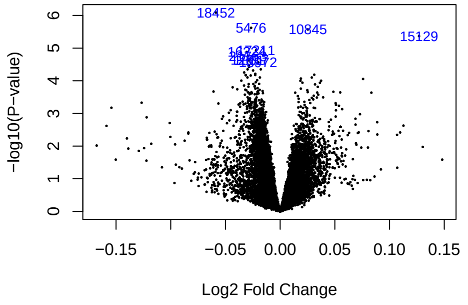


```
## gestage_delivery
```

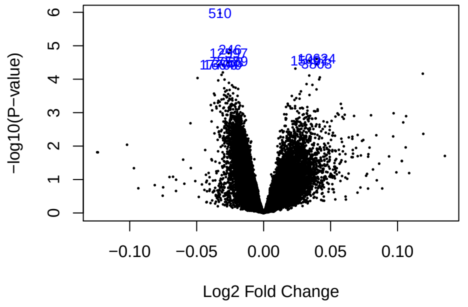

Map between manufacturer identifiers and gene symbols.

```
e2s = toTable(hta20transcriptclusterSYMBOL)
map_gene_symbol = function(gene_table) {
  prob_ids = rownames(gene_table)
  symbol = sapply(prob_ids,function(prob_id) {
    matching_symbol = e2s$symbol[prob_id==e2s$probe_id]
    if(length(matching_symbol)==0) matching_symbol = "No_Symbol_Found"
    matching_symbol
  }) %>% unlist
  gene_table = cbind(gene_table,symbol=symbol,stringsAsFactors=FALSE)
  gene_table
}
gene_table_list = lapply(gene_table_list,map_gene_symbol)
```

Print genes that are below an FDR of 0.1.

```
for(i in 1:length(gene_table_list)) {
  separator = "-----------"
  cat(separator,names(gene_table_list)[i],separator,"\n")
```

```
  gene_table_subset = subset(gene_table_list[[i]],adj.P.Val < 0.1)
  gene_table_subset %<>% as_tibble()
  print(gene_table_subset)
  cat(separator,names(gene_table_list)[i],separator,"\n\n")
}
## ----------- time_to_delivery -----------
## # A tibble: 170 x 7
##      logFC AveExpr     t     P.Value adj.P.Val     B symbol
##      <dbl>   <dbl> <dbl>       <dbl>     <dbl> <dbl> <chr>
##  1 -0.0587    4.87 -5.57 0.000000817    0.0181  5.15 IFNL1
##  2 -0.0265    4.44 -5.28 0.00000239     0.0198  4.10 NEURL1B
##  3  0.0254    4.67  5.24 0.00000268     0.0198  3.99 INPP5A
##  4  0.127     5.67  5.11 0.00000429     0.0238  3.53 SNORD18A
##  5 -0.0220    4.81 -4.83 0.0000117      0.0487  2.55 KRT33A
##  6 -0.0306    5.22 -4.79 0.0000132      0.0487  2.43 PAQR4
##  7 -0.0295    5.59 -4.71 0.0000179      0.0566  2.14 MICB
##  8 -0.0279    5.02 -4.64 0.0000227      0.0583  1.90 RBMS2
##  9 -0.0286    4.68 -4.63 0.0000237      0.0583  1.86 ATP1A4
## 10 -0.0202    5.00 -4.59 0.0000273      0.0605  1.73 GAS6
## # ... with 160 more rows
## ----------- time_to_delivery -----------
##
## ----------- gestage_delivery -----------
## # A tibble: 11 x 7
##      logFC AveExpr     t    P.Value adj.P.Val     B symbol
##      <dbl>   <dbl> <dbl>      <dbl>     <dbl> <dbl> <chr>
##  1 -0.0326    4.77 -5.50 0.00000106    0.0236  4.98 LSP1P5
##  2 -0.0250    4.86 -4.79 0.0000133     0.0821  2.51 TFAP2E
##  3 -0.0261    3.75 -4.73 0.0000167     0.0821  2.29 PRG2
##  4  0.0396    5.20  4.61 0.0000246     0.0821  1.92 MAPK8
##  5  0.0344    6.15  4.57 0.0000283     0.0821  1.78 KCTD5
##  6 -0.0202    5.32 -4.57 0.0000287     0.0821  1.77 MTX1
##  7 -0.0265    6.06 -4.54 0.0000316     0.0821  1.68 BOLA1
##  8  0.0396    4.45  4.51 0.0000350     0.0821  1.58 PRKCI
##  9 -0.0334    4.46 -4.50 0.0000368     0.0821  1.53 ETV4
## 10 -0.0307    3.83 -4.50 0.0000370     0.0821  1.52 FNDC8
## 11  0.0237    4.67  4.42 0.0000482     0.0971  1.27 INPP5A
## ----------- gestage_delivery -----------
```

Write to text file.

```
for(i in 1:length(gene_table_list)) {
  file_name_processed = paste0(
    "time_to_delivery_",
    names(gene_table_list)[i],
    "_",
    params$treatment,
    ".csv"
    )
  cat("writting:", file_name_processed, "\n")
  gene_table_list[[i]] %>%
```

```
    as_tibble() %>%
    write_csv(path = file_name_processed)
}
## writting: time_to_delivery_time_to_delivery_H1N1.csv
## writting: time_to_delivery_gestage_delivery_H1N1.csv
```

7 Network Analysis

Network analysis on time to event table.

```
gene_table = gene_table_list[[1]]
```

7.1 Download Gene Network

Download proteins for human species (code is 9606). Consider interactions at 0.9 confidence. From the STRING website: "In STRING, each protein-protein interaction is annotated with one or more 'scores'. Importantly, these scores do not indicate the strength or the specificity of the interaction. Instead, they are indicators of confidence, i.e. how likely STRING judges an interaction to be true, given the available evidence. All scores rank from 0 to 1, with 1 being the highest possible confidence. A score of 0.5 would indicate that roughly every second interaction might be erroneous (i.e., a false positive)."

```
string_db = STRINGdb$new(version="10", species = 9606,
                         score_threshold = 900, input_directory = "")
```

Check how many proteins are in the database.

```
string_proteins = string_db$get_proteins()
dim(string_proteins)
## [1] 20457     4
```

Map gene names to identifiers used in the database.

```
mapped = string_db$map(gene_table, "symbol", removeUnmappedRows = TRUE)
## Warning:  we couldn't map to STRING 20% of your identifiers
interactions = string_db$get_interactions(mapped$STRING_id)
interactions = data.frame(from = interactions$from,
                          to = interactions$to,
                          combined_score = interactions$combined_score)
dim(interactions)
## [1] 113416      3
head(interactions)
##                    from                    to combined_score
## 1 9606.ENSP00000003084 9606.ENSP00000302234            902
## 2 9606.ENSP00000003084 9606.ENSP00000302961            979
## 3 9606.ENSP00000003084 9606.ENSP00000305372            927
## 4 9606.ENSP00000003084 9606.ENSP00000308236            919
## 5 9606.ENSP00000003084 9606.ENSP00000308541            935
## 6 9606.ENSP00000003084 9606.ENSP00000309591            961
```

7.2 Find Subgraph

Fit a Beta-Uniform model.

```
pval = mapped$P.Value
names(pval) = mapped$STRING_id
fb = fitBumModel(pval)
## Warning in bumOptim(x = x, starts): One or both parameters are on the limit
## of the defined parameter space
```

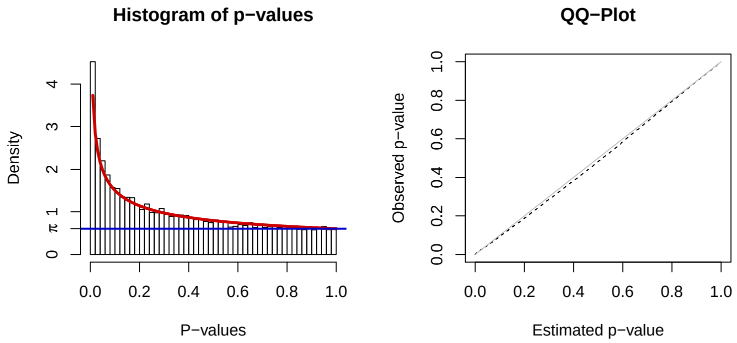


```
fb
## Beta-Uniform-Mixture (BUM) model
##
## 17532 pvalues fitted
##
## Mixture parameter (lambda):  0.000
## shape parameter (a):         0.605
## log-likelihood:          2640.5
```

Set the `fdr` parameter which can be interpreted as the FDR of the subgraph. Smaller values will produce a smaller maximum subgraph. You should try a few values (e.g. 0.05, 0.01, 0.001) to obtain a reasonable small subgraph that permits biological interpretation. First, we convert the interaction table into an igraph object and make the nodes names human readible. Then we search for the optimal subgraph.

```
fdr_vec = seq(0.1,0.01,-0.01)
network = graph_from_data_frame(interactions)
module_list = mclapply(fdr_vec, function(fdr) {
  scores = scoreNodes(network, fb, fdr = fdr)
  module = runFastHeinz(network, scores)
  module
}, mc.cores = 4)
module_lengths = sapply(module_list,function(module) length(V(module)))
tb_module = tibble(id = 1:length(module_lengths),
                   num_nodes = module_lengths,
                   fdr = fdr_vec)
ggplot(tb_module, aes(fdr,num_nodes)) +
  geom_line() +
  geom_point(size = 2)
```

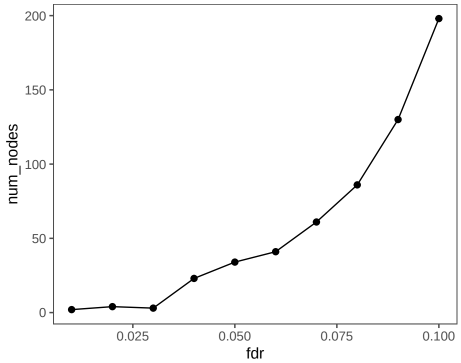


```
model_sel = tb_module %>% filter(fdr == 0.05) %>% .$id
module = module_list[[model_sel]]
module
## IGRAPH 381b5e6 DN-- 34 55 --
## + attr: name (v/c), score (v/n), combined_score (e/n)
## + edges from 381b5e6 (vertex names):
##  [1] 9606.ENSP00000007722->9606.ENSP00000371067
##  [2] 9606.ENSP00000219070->9606.ENSP00000219271
##  [3] 9606.ENSP00000219070->9606.ENSP00000350941
##  [4] 9606.ENSP00000219070->9606.ENSP00000353483
##  [5] 9606.ENSP00000219070->9606.ENSP00000380334
##  [6] 9606.ENSP00000229135->9606.ENSP00000270139
##  [7] 9606.ENSP00000229135->9606.ENSP00000349365
##  [8] 9606.ENSP00000229135->9606.ENSP00000369553
## + ... omitted several edges
```

7.3 Visualize Network

Differential expression is coloured in red (upregulated), green (downregulated), and white (neutral). Shapes represent scores: rectangles are negative and circles are positive.

Note that in limma, for continuous predictors, the log-fold changes (log-fc) are the regression coefficients.

```
plot_network = function(color_name) {
  if(length(V(module)) < 2)
    return("need two or more nodes")
  set.seed(0xdada)
  module_df = ggnetwork(module, layout = "kamadakawai")
  module_df$x = c(module_df$x)
  module_df$y = c(module_df$y)
  module_df$xend = c(module_df$xend)
  module_df$yend = c(module_df$yend)
  module_df %<>% mutate(sign_score = factor(sign(module_df$score)))
  ids = sapply(module_df$vertex.names,
               function(id) which(id == mapped$STRING_id)[1])
  module_df %<>% mutate(t_statistic = mapped$t[ids])
  module_df %<>% mutate(coefficient = mapped$logFC[ids])
  module_df %<>% mutate(symbol = mapped$symbol[ids])
```

```
    ggplot(module_df, aes(x = x, y = y, xend = xend, yend = yend)) +
      geom_edges() +
      geom_nodelabel_repel(aes(label = symbol),
                           box.padding = unit(1, "lines"),
                           alpha = 0.3) +
      geom_nodes(aes_string(shape = "sign_score", color = color_name),
                 size = 6) +
      ggtitle(paste0("Time to Delivery (FDR = ",fdr_vec[model_sel],")")) +
      scale_color_gradient2(midpoint = 0, low = "blue", mid = "white",
                            high = "red", space = "Lab" ) +
      theme_blank()
  }
  set.seed(0xdada)
  network_t_statistic = plot_network("t_statistic")
  save(network_t_statistic,
       file = paste0("network_t_statistic_",params$treatment,".Rdata"))
  network_t_statistic
```

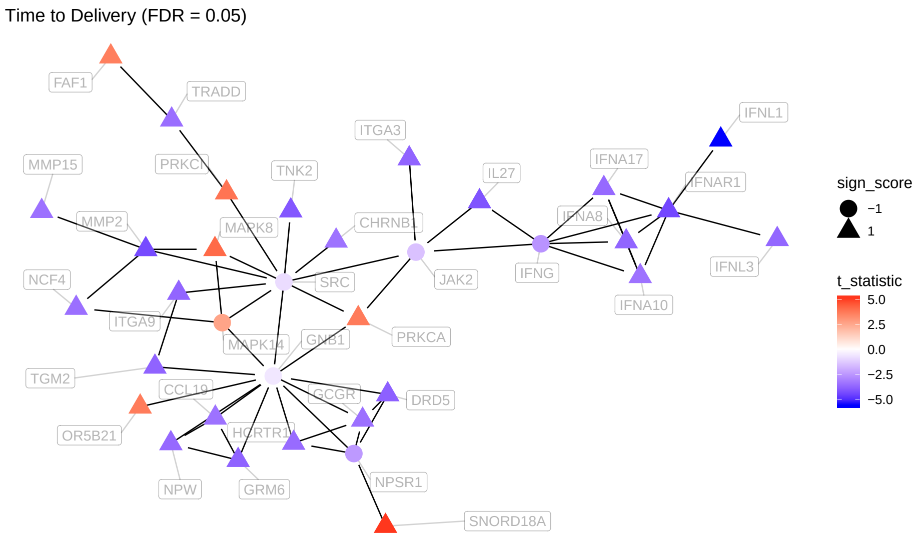


```
  network_log_coefficient = plot_network("coefficient")
  save(network_log_coefficient,
       file = paste0("network_log_coefficient_",params$treatment,".Rdata"))
  network_log_coefficient
```

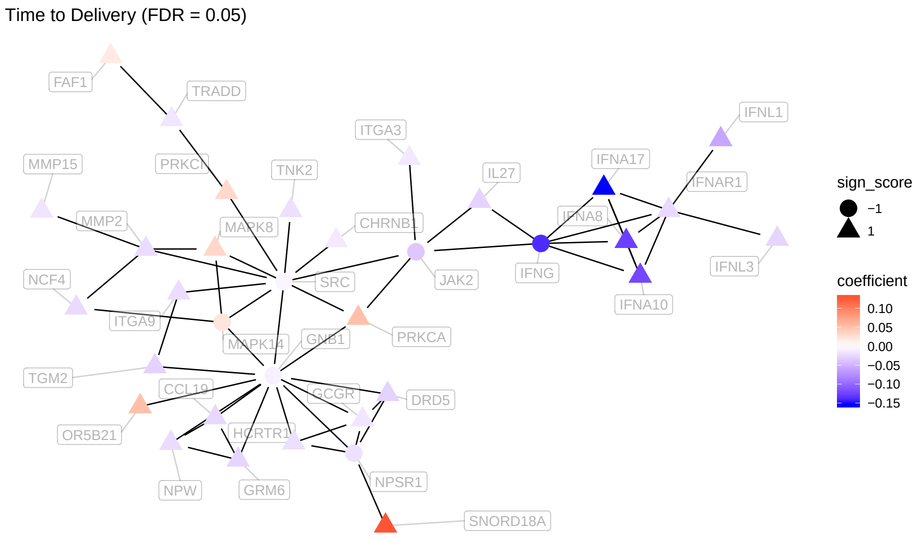

7.4 Single Genes Expression

Scatter plot for some genes in the network.

```
plot_node = function(gene_name) {
  probe_id = e2s[which(e2s$symbol == gene_name), "probe_id"]
  tb_expr = tibble(
    expr = exprs(eset)[which(rownames(exprs(eset)) == probe_id),],
    time_to_delivery = pData(eset)$time_to_delivery,
    gestage_delivery = pData(eset)$gestage_delivery
  )
  fit_expr = lm(expr ~ gestage_delivery, tb_expr)
  fit_time = lm(time_to_delivery ~ gestage_delivery, tb_expr)
  tb_expr %<>% mutate(res_expr = residuals(fit_expr))
  tb_expr %<>% mutate(res_time_to_delivery = residuals(fit_time))
  ggplot(tb_expr, aes(res_time_to_delivery, res_expr)) +
    geom_smooth(method = lm, se = FALSE, color = "grey", size = 2) +
    geom_point(size = 2) +
    ggtitle(gene_name) +
    xlab("resid(lm(time_to_delivery ~ gestage_delivery)") +
    ylab("resid(lm(expr ~ gestage_delivery))")
}
plot_node("IFNL1")
```

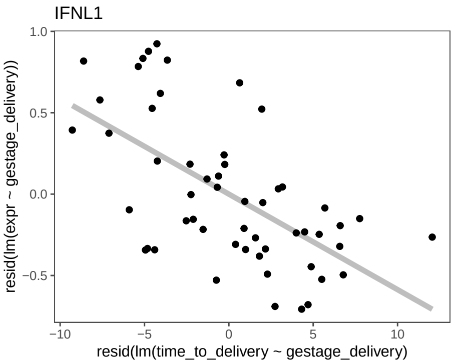


```
plot_node("SNORD18A")
```

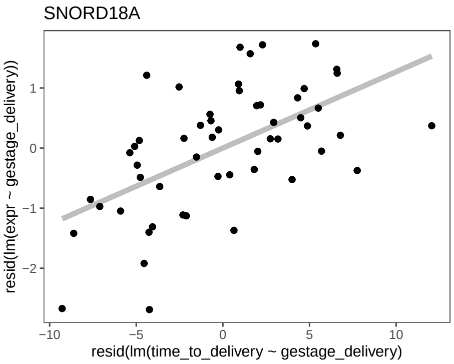

8 Pathways Analysis

Calculate number of genes that our network on known pathways have in common. Only keep pathways that overlap at least 5% of genes. Visualize pathway overlap.

```
plot_pathway = function(module) {
  if(length(V(module)) < 2)
    return("need two or more nodes")

  # get human pathways form kegg database
  pathways = keggList("pathway", "hsa")
  human_pathways = sub("path:", "", names(pathways))

  # kegg server only allow 10 request at the time
  n_request = ceiling(length(human_pathways)/10)
  chunk = function(x, n) split(x, sort(rank(x) %% n))
  chunks_pathways = chunk(1:length(human_pathways),n_request)

  # download from kegg server
  list_chunk_pathways = lapply(chunks_pathways,
                               function(one_chunk_pathways) {
    cat("download chunk:",one_chunk_pathways,"\n")
    pathway_ids = human_pathways[one_chunk_pathways]
    setNames(keggGet(pathway_ids), pathway_ids)
    })
```

```
    # flatten list of lists
    all_pathways = unlist(list_chunk_pathways, recursive=FALSE)

    # check for all human pathways
    module_df = ggnetwork(module)
    ids = sapply(module_df$vertex.names,
                 function(id) which(id == mapped$STRING_id)[1])
    tb_pways = lapply(all_pathways, function(pway) {
      gene_desc = pway$GENE[c(F,T)]
      if(length(gene_desc) > 0) {
        gene_symbol = sapply(strsplit(gene_desc, split = ";"),
                             function(desc) desc[1])
        overlap = mean(gene_symbol %in% unique(mapped$symbol[ids]))
      } else {
        overlap = 0
      }
      tibble(
        name = pway$NAME,
        overlap = overlap
        )
    }) %>% bind_rows
    tb_pways %<>%
      dplyr::filter(overlap > 0.04) %>%
      dplyr::arrange(desc(overlap))
    tb_pways$name %<>% str_replace(" - Homo sapiens \\(human\\)","")
    tb_pways$name %<>% factor(levels = rev(tb_pways$name))
    ggplot(tb_pways, aes(x = 100*overlap,y = name)) +
      geom_point(size = 2) +
      theme(legend.position="none") +
      xlab("Overlap in %") +
      ylab("Pathway Name")
  }
  plot_pathway(module)
  ## download chunk:  1  2  3  4  5  6  7  8  9
  ## download chunk:  10  11  12  13  14  15  16  17  18  19
  ## download chunk:  20  21  22  23  24  25  26  27  28  29
  ## download chunk:  30  31  32  33  34  35  36  37  38  39
  ## download chunk:  40  41  42  43  44  45  46  47  48  49
  ## download chunk:  50  51  52  53  54  55  56  57  58  59
  ## download chunk:  60  61  62  63  64  65  66  67  68  69
  ## download chunk:  70  71  72  73  74  75  76  77  78  79
  ## download chunk:  80  81  82  83  84  85  86  87  88  89
  ## download chunk:  90  91  92  93  94  95  96  97  98  99
  ## download chunk:  100  101  102  103  104  105  106  107  108  109
  ## download chunk:  110  111  112  113  114  115  116  117  118  119
  ## download chunk:  120  121  122  123  124  125  126  127  128  129
  ## download chunk:  130  131  132  133  134  135  136  137  138  139
  ## download chunk:  140  141  142  143  144  145  146  147  148  149
  ## download chunk:  150  151  152  153  154  155  156  157  158  159
  ## download chunk:  160  161  162  163  164  165  166  167  168  169
```

```
## download chunk: 170 171 172 173 174 175 176 177 178 179
## download chunk: 180 181 182 183 184 185 186 187 188 189
## download chunk: 190 191 192 193 194 195 196 197 198 199
## download chunk: 200 201 202 203 204 205 206 207 208 209
## download chunk: 210 211 212 213 214 215 216 217 218 219
## download chunk: 220 221 222 223 224 225 226 227 228 229
## download chunk: 230 231 232 233 234 235 236 237 238 239
## download chunk: 240 241 242 243 244 245 246 247 248 249
## download chunk: 250 251 252 253 254 255 256 257 258 259
## download chunk: 260 261 262 263 264 265 266 267 268 269
## download chunk: 270 271 272 273 274 275 276 277 278 279
## download chunk: 280 281 282 283 284 285 286 287 288 289
## download chunk: 290 291 292 293 294 295 296 297 298 299
## download chunk: 300 301 302 303 304 305 306 307 308 309
## download chunk: 310 311 312 313 314 315 316 317 318 319
## download chunk: 320 321 322 323 324 325 326 327 328
## download chunk: 329 330 331 332 333 334 335 336 337
```

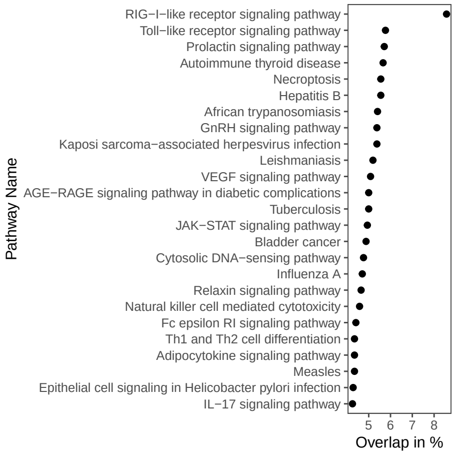

9 Time to Delivery Prediction

Use Lasso to predict time to delivery from gene expressions.

```
set.seed(0xdada)
exprs_table = t(exprs(eset))
sample_table = eset@phenoData@data
glmnet_cv = cv.glmnet(x = exprs_table,
                      y = sample_table$time_to_delivery)
plot(glmnet_cv)
```

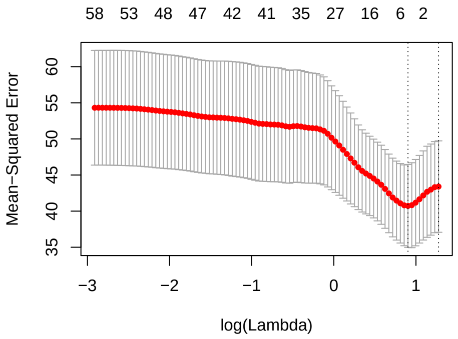


```
glmnet_fit = glmnet(x = exprs_table,
                    y = sample_table$time_to_delivery,
                    lambda = glmnet_cv$lambda.min)
nonzero = which(coef(glmnet_fit) > 0)
prob_ids_nonzero = rownames(coef(glmnet_fit))[nonzero]
e2s[which(e2s$probe_id %in% prob_ids_nonzero),]
##             probe_id  symbol
## 6306 TC04000513.hg.1 MIR3684
```

Session Info

```
sessionInfo()
## R version 3.5.1 (2018-07-02)
## Platform: x86_64-apple-darwin15.6.0 (64-bit)
## Running under: macOS  10.15.3
##
## Matrix products: default
## BLAS: /Library/Frameworks/R.framework/Versions/3.5/Resources/lib/libRblas.0.dylib
## LAPACK: /Library/Frameworks/R.framework/Versions/3.5/Resources/lib/libRlapack.dylib
##
## locale:
## [1] en_US.UTF-8/en_US.UTF-8/en_US.UTF-8/C/en_US.UTF-8/en_US.UTF-8
##
## attached base packages:
## [1] stats4    parallel  stats     graphics  grDevices utils     datasets
## [8] methods   base
##
## other attached packages:
##  [1] pd.hta.2.0_3.12.2            DBI_1.0.0
##  [3] RSQLite_2.1.1                readr_1.3.1
##  [5] sna_2.4                      network_1.15
##  [7] statnet.common_4.2.0         ggthemes_4.1.1
##  [9] ggnetwork_0.5.1              intergraph_2.0-2
## [11] igraph_1.2.4.1               KEGGREST_1.22.0
## [13] DLBCL_1.22.0                 BioNet_1.42.0
## [15] RBGL_1.58.2                  graph_1.60.0
## [17] STRINGdb_1.22.0              dplyr_0.8.3
## [19] tibble_2.1.3                 stringr_1.4.0
```

```
## [21] statmod_1.4.32                   magrittr_1.5
## [23] ggfortify_0.4.7                  ggplot2_3.2.1
## [25] hta20transcriptcluster.db_8.7.0 org.Hs.eg.db_3.7.0
## [27] AnnotationDbi_1.44.0             glmnet_2.0-18
## [29] foreach_1.4.4                    Matrix_1.2-17
## [31] genefilter_1.64.0                affycoretools_1.54.0
## [33] limma_3.38.3                     oligo_1.46.0
## [35] Biostrings_2.50.2                XVector_0.22.0
## [37] IRanges_2.16.0                   S4Vectors_0.20.1
## [39] Biobase_2.42.0                   oligoClasses_1.44.0
## [41] BiocGenerics_0.28.0              BiocStyle_2.10.0
##
## loaded via a namespace (and not attached):
##   [1] utf8_1.1.4                  proto_1.0.0
##   [3] R.utils_2.8.0               tidyselect_0.2.5
##   [5] htmlwidgets_1.3             grid_3.5.1
##   [7] BiocParallel_1.16.6         munsell_0.5.0
##   [9] codetools_0.2-16            preprocessCore_1.44.0
##  [11] chron_2.3-53                withr_2.1.2
##  [13] colorspace_1.4-1            Category_2.48.1
##  [15] OrganismDbi_1.24.0          knitr_1.22
##  [17] rstudioapi_0.10             labeling_0.3
##  [19] GenomeInfoDbData_1.2.0      hwriter_1.3.2
##  [21] farver_2.0.1                bit64_0.9-7
##  [23] coda_0.19-3                 vctrs_0.2.1
##  [25] xfun_0.6                    biovizBase_1.30.1
##  [27] affxparser_1.54.0           R6_2.4.1
##  [29] GenomeInfoDb_1.18.2         locfit_1.5-9.1
##  [31] AnnotationFilter_1.6.0      bitops_1.0-6
##  [33] reshape_0.8.8               DelayedArray_0.8.0
##  [35] assertthat_0.2.1            scales_1.1.0
##  [37] nnet_7.3-12                 gtable_0.3.0
##  [39] affy_1.60.0                 ggbio_1.30.0
##  [41] ensembldb_2.6.8             rlang_0.4.2
##  [43] zeallot_0.1.0               splines_3.5.1
##  [45] rtracklayer_1.42.2          lazyeval_0.2.2
##  [47] acepack_1.4.1               dichromat_2.0-0
##  [49] checkmate_1.9.4             BiocManager_1.30.4
##  [51] yaml_2.2.0                  reshape2_1.4.3
##  [53] GenomicFeatures_1.34.8      backports_1.1.5
##  [55] Hmisc_4.2-0                 tools_3.5.1
##  [57] bookdown_0.9                affyio_1.52.0
##  [59] gplots_3.0.1.1              ff_2.2-14
##  [61] RColorBrewer_1.1-2          gsubfn_0.7
##  [63] Rcpp_1.0.3                  hash_2.2.6.1
##  [65] plyr_1.8.5                  base64enc_0.1-3
##  [67] progress_1.2.2              zlibbioc_1.28.0
##  [69] purrr_0.3.3                 RCurl_1.95-4.12
##  [71] prettyunits_1.1.0           rpart_4.1-15
##  [73] sqldf_0.4-11                ggrepel_0.8.1
##  [75] SummarizedExperiment_1.12.0 cluster_2.0.9
```

```
##  [77] data.table_1.12.6          ProtGenerics_1.14.0
##  [79] matrixStats_0.55.0         hms_0.5.2
##  [81] evaluate_0.13              xtable_1.8-4
##  [83] XML_3.98-1.19              gcrma_2.54.0
##  [85] gridExtra_2.3              compiler_3.5.1
##  [87] biomaRt_2.38.0             KernSmooth_2.23-15
##  [89] crayon_1.3.4               ReportingTools_2.22.1
##  [91] R.oo_1.22.0                htmltools_0.3.6
##  [93] GOstats_2.48.0             Formula_1.2-3
##  [95] tidyr_1.0.0                geneplotter_1.60.0
##  [97] cli_2.0.1                  R.methodsS3_1.7.1
##  [99] gdata_2.18.0               GenomicRanges_1.34.0
## [101] pkgconfig_2.0.3            GenomicAlignments_1.18.1
## [103] foreign_0.8-71             annotate_1.60.1
## [105] AnnotationForge_1.24.0     VariantAnnotation_1.28.13
## [107] digest_0.6.23              rmarkdown_1.12
## [109] htmlTable_1.13.2           edgeR_3.24.3
## [111] GSEABase_1.44.0            curl_4.3
## [113] Rsamtools_1.34.1           gtools_3.8.1
## [115] lifecycle_0.1.0            PFAM.db_3.7.0
## [117] fansi_0.4.1                BSgenome_1.50.0
## [119] pillar_1.4.3               lattice_0.20-38
## [121] GGally_1.4.0               httr_1.4.0
## [123] plotrix_3.7-5              survival_2.44-1.1
## [125] GO.db_3.7.0                glue_1.3.1
## [127] png_0.1-7                  iterators_1.0.10
## [129] bit_1.1-14                 Rgraphviz_2.26.0
## [131] stringi_1.4.5              blob_1.1.1
## [133] DESeq2_1.22.2              latticeExtra_0.6-28
## [135] caTools_1.17.1.2           memoise_1.1.0
```
