# Supplementary material for: Influenza-Induced Interferon Lambda Response Is Associated With Longer Time to Delivery Among Pregnant Kenyan Women
Source: Front Immunol. 2020 Mar 17;11:452. doi: 10.3389/fimmu.2020.00452 (PMC7089959; doi:10.3389/fimmu.2020.00452)
Supplement: Supplementary file 2 [file Presentation_1.zip › TimeToDelivery_Microarray_Mock.pdf]

Separate Mock/H1N1 Time to Delivery Analysis on Microarray Data

Christof Seiler

3 February 2020

Contents

1 Goal . . . 2
2 Prerequisites . . . 2
3 Import Data . . . 3
4 Preprocessing . . . 5
5 Data Exploration . . . 5
6 Differential Expression Analyses . . . 7
7 Network Analysis . . . 10
7.1 Download Gene Network . . . 10
7.2 Find Subgraph . . . 11
7.3 Visualize Network . . . 12
7.4 Single Genes Expression . . . 13
8 Pathways Analysis . . . 14
9 Time to Delivery Prediction . . . 16
Session Info . . . 16

1 Goal

- Load and normalize data using `oligo`
- Differential analysis using `limma`
- Network analysis using `BioNet`
- Pathway analysis using `KEGGREST`

2 Prerequisites

Install necessary packages from bioconductor repository. Run this code only once to install packages.

```
pkgs_needed = c("oligo","limma","affycoretools","genefilter","glmnet",
                "hta20transcriptcluster.db","ggfortify","magrittr",
                "statmod","stringr","tibble","dplyr",
                "STRINGdb","BioNet","DLBCL","org.Hs.eg.db","KEGGREST",
                "igraph","intergraph","ggnetwork","ggthemes","readr")
letsinstall = setdiff(pkgs_needed, installed.packages())
if (length(letsinstall) > 0) {
  source("http://bioconductor.org/biocLite.R")
  biocLite(letsinstall)
}
```

Load packages.

```
library("oligo")
library("limma")
library("affycoretools")
library("genefilter")
library("glmnet")
library("hta20transcriptcluster.db")
library("ggfortify")
library("magrittr")
library("statmod")
library("stringr")
library("tibble")
library("dplyr")
library("STRINGdb")
library("BioNet")
library("DLBCL")
library("org.Hs.eg.db")
library("KEGGREST")
library("igraph")
library("intergraph")
library("ggnetwork")
library("ggthemes")
library("sna")
library("statnet.common")
library("network")
library("readr")
theme_set(theme_few())
```

```
scale_colour_discrete = function(...) scale_colour_few()
```

3 Import Data

Then load Affymetrix CEL files. At this stage, Bioconductor will automatically download the necessary annotation packages and install them for us. Add time to delivery variable.

```
sample_table = read.csv("sample_table.csv")
cel_filenames = paste0(as.character(sample_table$array_name),".CEL")
sample_table %<>% mutate(
  cel_filename = cel_filenames,
  sample_name = paste(sample_table$sample_id,
                      sample_table$condition,
                      sample_table$treatment,sep = "_")
  )
write_csv(sample_table, path = "sample_table_cel.csv")
params$treatment
## [1] "Mock"
sample_table %<>% filter(treatment == params$treatment)
sample_table$time_to_delivery = sample_table$gestage_enroll -
  sample_table$gestage_delivery
pd = as(sample_table, "AnnotatedDataFrame")
cel_filenames = paste0(as.character(sample_table$array_name),".CEL")
rawData = read.celfiles(cel_filenames, phenoData = pd,
                        sampleNames = sample_table$sample_name)
## Loading required package: pd.hta.2.0
## Loading required package: RSQLite
## Loading required package: DBI
## Platform design info loaded.
## Reading in : Nicholas Bayless_US 1.1_(HTA-2_0).CEL
## Reading in : Nicholas Bayless_US 1.2_(HTA-2_0).CEL
## Reading in : Nicholas Bayless_US 1.3_(HTA-2_0).CEL
## Reading in : Nicholas Bayless_US 2.1_(HTA-2_0).CEL
## Reading in : Nicholas Bayless_US 2.2_(HTA-2_0).CEL
## Reading in : Nicholas Bayless_US 2.3_(HTA-2_0).CEL
## Reading in : Nicholas Bayless_US 3.2_(HTA-2_0).CEL
## Reading in : Nicholas Bayless_US 3.3_(HTA-2_0).CEL
## Reading in : Nicholas Bayless_US 4.3_(HTA-2_0).CEL
## Reading in : Nicholas Bayless_US 5.1_(HTA-2_0).CEL
## Reading in : Nicholas Bayless_US 5.3_(HTA-2_0).CEL
## Reading in : Nicholas Bayless_US 6.1_(HTA-2_0).CEL
## Reading in : Nicholas Bayless_US 6.2_(HTA-2_0).CEL
## Reading in : Nicholas Bayless_US 6.3_(HTA-2_0).CEL
## Reading in : Nicholas Bayless_7.1 -_(HTA-2_0).CEL
## Reading in : Nicholas Bayless_7.2 -_(HTA-2_0).CEL
## Reading in : Nicholas Bayless_7.3 -_(HTA-2_0).CEL
## Reading in : Nicholas Bayless_8.1 -_(HTA-2_0).CEL
## Reading in : Nicholas Bayless_8.2 -_(HTA-2_0).CEL
## Reading in : Nicholas Bayless_8.3 -_(HTA-2_0).CEL
## Reading in : Nicholas Bayless_9.1 -_(HTA-2_0).CEL
```

```
## Reading in : Nicholas Bayless_9.2 -_(HTA-2_0).CEL
## Reading in : Nicholas Bayless_9.3 -_(HTA-2_0).CEL
## Reading in : Nicholas Bayless_10.1 -_(HTA-2_0).CEL
## Reading in : Nicholas Bayless_10.2 -_(HTA-2_0).CEL
## Reading in : Nicholas Bayless_10.3 -_(HTA-2_0).CEL
## Reading in : Nicholas Bayless_11.1 -_(HTA-2_0).CEL
## Reading in : Nicholas Bayless_11.2 -_(HTA-2_0).CEL
## Reading in : Nicholas Bayless_11.3 -_(HTA-2_0).CEL
## Reading in : Nicholas Bayless_12.1 -_(HTA-2_0).CEL
## Reading in : Nicholas Bayless_12.2 -_(HTA-2_0).CEL
## Reading in : Nicholas Bayless_US 13.1_(HTA-2_0).CEL
## Reading in : Nicholas Bayless_US 13.2_(HTA-2_0).CEL
## Reading in : Nicholas Bayless_US 13.3_(HTA-2_0).CEL
## Reading in : Nicholas Bayless_US 14.1_(HTA-2_0).CEL
## Reading in : Nicholas Bayless_US 14.2_(HTA-2_0).CEL
## Reading in : Nicholas Bayless_US 14.3_(HTA-2_0).CEL
## Reading in : Nicholas Bayless_US 16.1_(HTA-2_0).CEL
## Reading in : Nicholas Bayless_US 16.2_(HTA-2_0).CEL
## Reading in : Nicholas Bayless_US 16.3_(HTA-2_0).CEL
## Reading in : Nicholas Bayless_US 17.1_(HTA-2_0).CEL
## Reading in : Nicholas Bayless_US 17.2_(HTA-2_0).CEL
## Reading in : Nicholas Bayless_US 17.3_(HTA-2_0).CEL
## Reading in : Nicholas Bayless_US 18.1_(HTA-2_0).CEL
## Reading in : Nicholas Bayless_US 18.3_(HTA-2_0).CEL
## Reading in : Nicholas Bayless_US 19.1_(HTA-2_0).CEL
## Reading in : Nicholas Bayless_US 19.2_(HTA-2_0).CEL
## Reading in : Nicholas Bayless_US 19.3_(HTA-2_0).CEL
## Reading in : Nicholas Bayless_US 20.1_(HTA-2_0).CEL
## Reading in : Nicholas Bayless_US 20.2_(HTA-2_0).CEL
## Reading in : Nicholas Bayless_US 20.3_(HTA-2_0).CEL
## Reading in : Nicholas Bayless_US 21.1_(HTA-2_0).CEL
## Reading in : Nicholas Bayless_US 21.2_(HTA-2_0).CEL
## Reading in : Nicholas Bayless_US 21.3_(HTA-2_0).CEL
## Warning in read.celfiles(cel_filenames, phenoData = pd, sampleNames =
## sample_table$sample_name): 'channel' automatically added to varMetadata in
## phenoData.
rawData
## HTAFeatureSet (storageMode: lockedEnvironment)
## assayData: 6892960 features, 54 samples
##   element names: exprs
## protocolData
##   rowNames: 1 2 ... 54 (54 total)
##   varLabels: exprs dates
##   varMetadata: labelDescription channel
## phenoData
##   rowNames: 1 2 ... 54 (54 total)
##   varLabels: X sample_id ... time_to_delivery (16 total)
##   varMetadata: labelDescription channel
## featureData: none
## experimentData: use 'experimentData(object)'
## Annotation: pd.hta.2.0
```

4 Preprocessing

Background subtraction, normalization and summarization using median-polish.

```
eset = rma(rawData)
## Background correcting
## Normalizing
## Calculating Expression
```

Get rid of background probes and annotate using functions in `affycoretools` package.

```
dbGetQuery(db(pd.hta.2.0), "select * from type_dict;")
##   type                                          type_id
## 1    1                                             main
## 2    2                     Antigenomic background control
## 3    3                           control->affx->bac_spike
## 4    4                         control->affx->polya_spike
## 5    5 ERCC (External RNA Controls Consortium) step control
## 6    6      Exonic normalization control (Positive Control)
## 7    7    Intronic normalization control (Negative Control)
## 8    8                                  Positive Control
table(getMainProbes("pd.hta.2.0")$type)
##
##     1     2     3     4     5     6     7
## 67516    23     4     4   155   698   646
eset = getMainProbes(eset)
```

Filter probes that we cannot map to symbols.

```
e2s = toTable(hta20transcriptclusterSYMBOL)
prob_ids = rownames(exprs(eset))
keep_ids = which(prob_ids %in% e2s$probe_id)
eset = ExpressionSet(assayData = exprs(eset)[keep_ids,],
                     phenoData = phenoData(eset),
                     experimentData = experimentData(eset),
                     annotation = annotation(eset))
```

Write processed expressions to file for GEO upload.

```
geo_exprs_rma = exprs(eset)
colnames(geo_exprs_rma) = pData(eset)$sample_name
geo_exprs_rma %<>% as_tibble(rownames = "ID_REF")
write_csv(geo_exprs_rma, path = paste0("time_to_delivery_rma_", params$treatment, ".csv"))
```

5 Data Exploration

PCA plot of normalized expressions.

```
res_pca = prcomp(t(exprs(eset)),scale. = FALSE)
screeplot(res_pca)
```

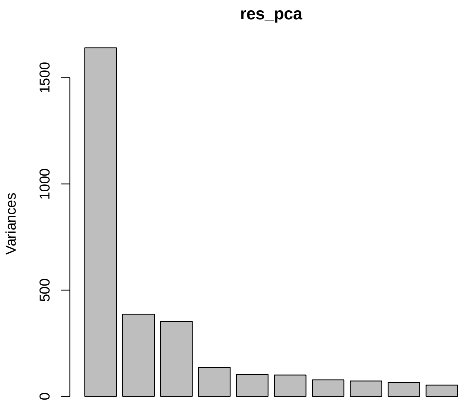


```
sample_table = eset@phenoData@data
asp_ratio = res_pca$sdev[2]^2/res_pca$sdev[1]^2
autoplot(res_pca, data = sample_table, colour = 'treatment',
         shape = 'condition', size = 3, asp = asp_ratio)
```

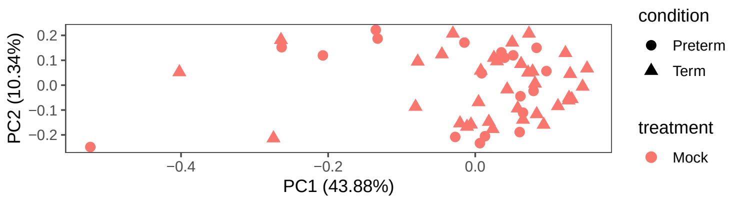


```
rownames(sample_table) = paste(sample_table$sample_id,
                               sample_table$treatment,sep = "_")
autoplot(res_pca, data = sample_table,
         shape = FALSE, label = TRUE, label.size = 3, asp = asp_ratio)
```

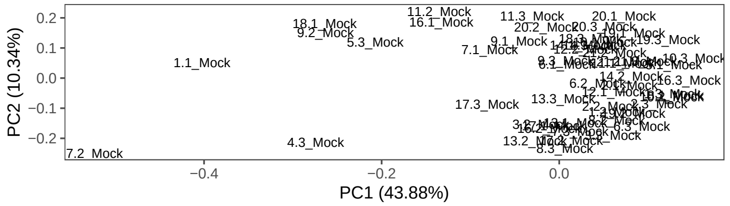

Check for batch effects.

```
sample_table$run_batch %<>% as.factor()
sample_table$run_day %<>% factor(levels(sample_table$run_day)[c(2:5,1,6)])
autoplot(res_pca, data = sample_table, colour = 'run_day',
         shape = 'condition', size = 3, asp = asp_ratio)
```

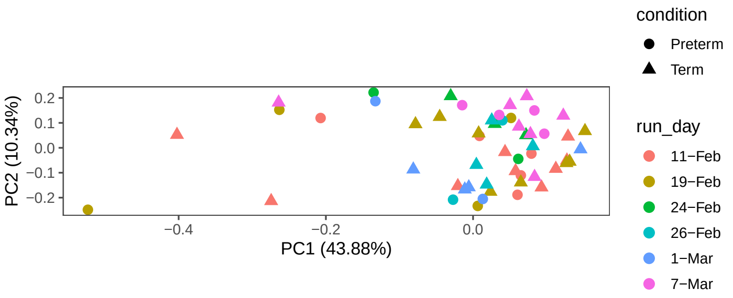


```
autoplot(res_pca, data = sample_table, colour = 'run_batch',
         shape = 'condition', size = 3, asp = asp_ratio)
```

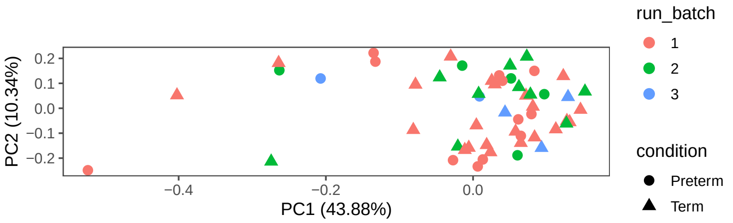

6 Differential Expression Analyses

Use `limma` for linear models to assess difference in expression. Define design matrix.

```
design = model.matrix(~ time_to_delivery + gestage_delivery, sample_table)
colnames(design) = str_replace(string = colnames(design),
                               pattern = ":",replacement = "_") %>%
  str_replace(string = .,pattern = "treatment",replacement = "")
colnames(design)
## [1] "(Intercept)"      "time_to_delivery" "gestage_delivery"
```

Choose mean expression threshold that maximizes number of differentially expressed genes. This approach is called automatic independent filtering and used in DESeq2.

```
mean_expr = rowMeans(exprs(eset))
thres_candidates = seq(min(mean_expr),quantile(mean_expr, probs = 0.95),1)
fit_list = lapply(thres_candidates, function(thres) {
  cat("Automatic independent filtering: thres = ", thres,"\n")
  # threshold
  eset_thres = ExpressionSet(assayData = exprs(eset)[mean_expr >= thres,],
                             phenoData = phenoData(eset),
                             experimentData = experimentData(eset),
                             annotation = annotation(eset))
  # fit model
  fit = lmFit(eset_thres, design)
  eBayes(fit)
})
## Automatic independent filtering: thres =  1.469981
## Automatic independent filtering: thres =  2.469981
## Automatic independent filtering: thres =  3.469981
## Automatic independent filtering: thres =  4.469981
```

```
## Automatic independent filtering: thres =  5.469981
## Automatic independent filtering: thres =  6.469981
num_sig = sapply(fit_list, function(fit) {
  coeffs = colnames(design)[-1]
  gene_table_combined = lapply(coeffs,function(coeff_name) {
    gene_table = topTable(fit, coef = coeff_name, adjust = "BH",
                          number = nrow(fit))
    gene_table %>% dplyr::filter(adj.P.Val < 0.1) %>%
      add_column(coeff = coeff_name)
  }) %>% bind_rows()
  nrow(gene_table_combined)
})
num_sig
## [1] 0 0 0 0 0 0
fit = fit_list[[which.max(num_sig)]]
```

Save results in a list of tables.

```
coeffs = colnames(design)[-1]
gene_table_list = lapply(coeffs, function(coeff_name) {
  gene_table = topTable(fit,
                        coef = coeff_name,
                        adjust = "BH",
                        number = nrow(fit))
  hist(gene_table$P.Value,breaks = 100,main = coeff_name)
  gene_table
})
```

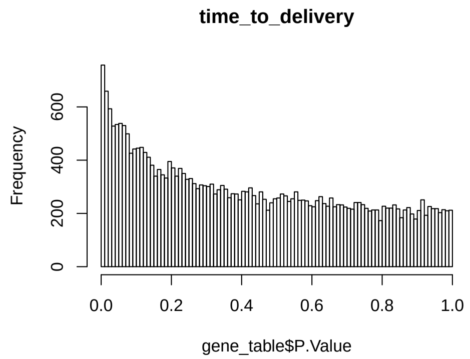

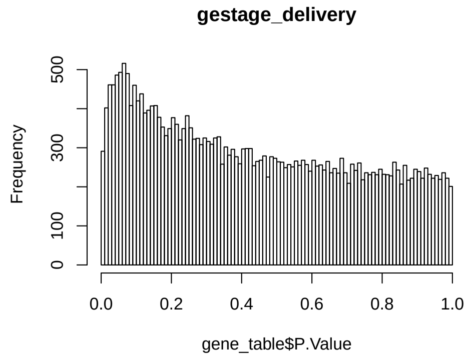

```
names(gene_table_list) = coeffs
```

Volcano plots for quality control.

```
for(coeff_name in coeffs) {
  cat(coeff_name,"\n")
  volcanoplot(fit,coef = coeff_name,highlight = 10)
}
## time_to_delivery
```

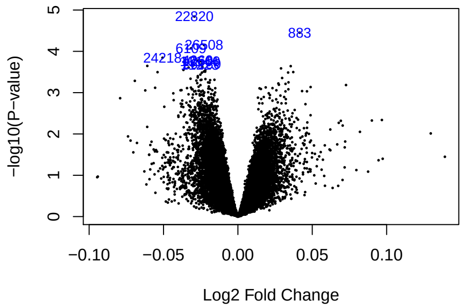


```
## gestage_delivery
```

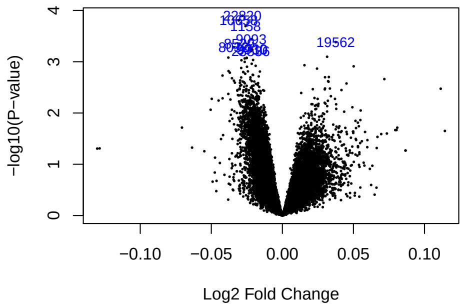

Map between manufacturer identifiers and gene symbols.

```
e2s = toTable(hta20transcriptclusterSYMBOL)
map_gene_symbol = function(gene_table) {
  prob_ids = rownames(gene_table)
  symbol = sapply(prob_ids,function(prob_id) {
    matching_symbol = e2s$symbol[prob_id==e2s$probe_id]
    if(length(matching_symbol)==0) matching_symbol = "No_Symbol_Found"
    matching_symbol
  }) %>% unlist
  gene_table = cbind(gene_table,symbol=symbol,stringsAsFactors=FALSE)
  gene_table
}
gene_table_list = lapply(gene_table_list,map_gene_symbol)
```

Print genes that are below an FDR of 0.1.

```
for(i in 1:length(gene_table_list)) {
  separator = "-----------"
  cat(separator,names(gene_table_list)[i],separator,"\n")
```

```
  gene_table_subset = subset(gene_table_list[[i]],adj.P.Val < 0.1)
  gene_table_subset %<>% as_tibble()
  print(gene_table_subset)
  cat(separator,names(gene_table_list)[i],separator,"\n\n")
}
## ----------- time_to_delivery -----------
## # A tibble: 0 x 7
## # ... with 7 variables: logFC <dbl>, AveExpr <dbl>, t <dbl>, P.Value <dbl>,
## #   adj.P.Val <dbl>, B <dbl>, symbol <chr>
## ----------- time_to_delivery -----------
##
## ----------- gestage_delivery -----------
## # A tibble: 0 x 7
## # ... with 7 variables: logFC <dbl>, AveExpr <dbl>, t <dbl>, P.Value <dbl>,
## #   adj.P.Val <dbl>, B <dbl>, symbol <chr>
## ----------- gestage_delivery -----------
```

Write to text file.

```
for(i in 1:length(gene_table_list)) {
  file_name_processed = paste0(
    "time_to_delivery_",
    names(gene_table_list)[i],
    "_",
    params$treatment,
    ".csv"
    )
  cat("writting:", file_name_processed, "\n")
  gene_table_list[[i]] %>%
    as_tibble() %>%
    write_csv(path = file_name_processed)
}
## writting: time_to_delivery_time_to_delivery_Mock.csv
## writting: time_to_delivery_gestage_delivery_Mock.csv
```

7 Network Analysis

Network analysis on time to event table.

```
gene_table = gene_table_list[[1]]
```

7.1 Download Gene Network

Download proteins for human species (code is 9606). Consider interactions at 0.9 confidence. From the STRING website: "In STRING, each protein-protein interaction is annotated with one or more 'scores'. Importantly, these scores do not indicate the strength or the specificity of the interaction. Instead, they are indicators of confidence, i.e. how likely STRING judges an interaction to be true, given the available evidence. All scores rank from 0 to 1, with 1 being the highest possible confidence. A score of 0.5 would indicate that roughly every second interaction might be erroneous (i.e., a false positive)."

```
string_db = STRINGdb$new(version="10", species = 9606,
                         score_threshold = 900, input_directory = "")
```

Check how many proteins are in the database.

```
string_proteins = string_db$get_proteins()
dim(string_proteins)
## [1] 20457     4
```

Map gene names to identifiers used in the database.

```
mapped = string_db$map(gene_table, "symbol", removeUnmappedRows = TRUE)
## Warning:  we couldn't map to STRING 26% of your identifiers
interactions = string_db$get_interactions(mapped$STRING_id)
interactions = data.frame(from = interactions$from,
                          to = interactions$to,
                          combined_score = interactions$combined_score)
dim(interactions)
## [1] 193485      3
head(interactions)
##                    from                   to combined_score
## 1 9606.ENSP00000003084 9606.ENSP00000302234            902
## 2 9606.ENSP00000003084 9606.ENSP00000302961            979
## 3 9606.ENSP00000003084 9606.ENSP00000305372            927
## 4 9606.ENSP00000003084 9606.ENSP00000308236            919
## 5 9606.ENSP00000003084 9606.ENSP00000308541            935
## 6 9606.ENSP00000003084 9606.ENSP00000309591            961
```

7.2 Find Subgraph

Fit a Beta-Uniform model.

```
pval = mapped$P.Value
names(pval) = mapped$STRING_id
fb = fitBumModel(pval)
## Warning in bumOptim(x = x, starts): One or both parameters are on the limit
## of the defined parameter space
```

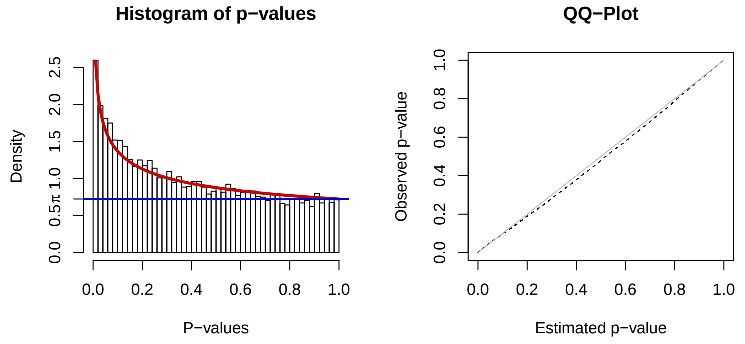


```
fb
## Beta-Uniform-Mixture (BUM) model
```

```
##
## 21768 pvalues fitted
##
## Mixture parameter (lambda):  0.000
## shape parameter (a):         0.724
## log-likelihood:          1271.6
```

Set the `fdr` parameter which can be interpreted as the FDR of the subgraph. Smaller values will produce a smaller maximum subgraph. You should try a few values (e.g. 0.05, 0.01, 0.001) to obtain a reasonable small subgraph that permits biological interpretation. First, we convert the interaction table into an igraph object and make the nodes names human readible. Then we search for the optimal subgraph.

```
fdr_vec = seq(0.1,0.01,-0.01)
network = graph_from_data_frame(interactions)
module_list = mclapply(fdr_vec, function(fdr) {
  scores = scoreNodes(network, fb, fdr = fdr)
  module = runFastHeinz(network, scores)
  module
}, mc.cores = 4)
module_lengths = sapply(module_list,function(module) length(V(module)))
tb_module = tibble(id = 1:length(module_lengths),
                   num_nodes = module_lengths,
                   fdr = fdr_vec)
ggplot(tb_module, aes(fdr,num_nodes)) +
  geom_line() +
  geom_point(size = 2)
```

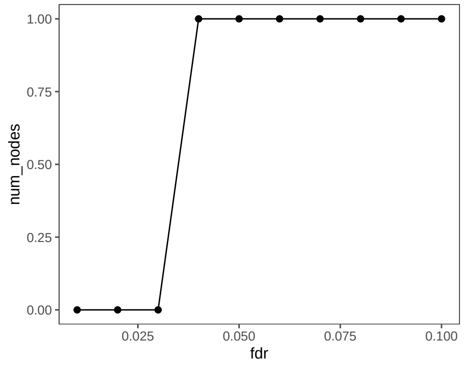


```
model_sel = tb_module %>% filter(fdr == 0.05) %>% .$id
module = module_list[[model_sel]]
module
## IGRAPH 0d4bab5 DN-- 1 0 --
## + attr: name (v/c), score (v/n), combined_score (e/n)
## + edges from 0d4bab5 (vertex names):
```

7.3 Visualize Network

Differential expression is coloured in red (upregulated), green (downregulated), and white (neutral). Shapes represent scores: rectangles are negative and circles are positive.

Note that in limma, for continuous predictors, the log-fold changes (log-fc) are the regression coefficients.

```
plot_network = function(color_name) {
  if(length(V(module)) < 2)
    return("need two or more nodes")
  set.seed(0xdada)
  module_df = ggnetwork(module, layout = "kamadakawai")
  module_df$x = c(module_df$x)
  module_df$y = c(module_df$y)
  module_df$xend = c(module_df$xend)
  module_df$yend = c(module_df$yend)
  module_df %<>% mutate(sign_score = factor(sign(module_df$score)))
  ids = sapply(module_df$vertex.names,
               function(id) which(id == mapped$STRING_id)[1])
  module_df %<>% mutate(t_statistic = mapped$t[ids])
  module_df %<>% mutate(coefficient = mapped$logFC[ids])
  module_df %<>% mutate(symbol = mapped$symbol[ids])
  ggplot(module_df, aes(x = x, y = y, xend = xend, yend = yend)) +
    geom_edges() +
    geom_nodelabel_repel(aes(label = symbol),
                         box.padding = unit(1, "lines"),
                         alpha = 0.3) +
    geom_nodes(aes_string(shape = "sign_score", color = color_name),
               size = 6) +
    ggtitle(paste0("Time to Delivery (FDR = ",fdr_vec[model_sel],")")) +
    scale_color_gradient2(midpoint = 0, low = "blue", mid = "white",
                          high = "red", space = "Lab" ) +
    theme_blank()
}
set.seed(0xdada)
network_t_statistic = plot_network("t_statistic")
save(network_t_statistic,
     file = paste0("network_t_statistic_",params$treatment,".Rdata"))
network_t_statistic
## [1] "need two or more nodes"
network_log_coefficient = plot_network("coefficient")
save(network_log_coefficient,
     file = paste0("network_log_coefficient_",params$treatment,".Rdata"))
network_log_coefficient
## [1] "need two or more nodes"
```

7.4 Single Genes Expression

Scatter plot for some genes in the network.

```
plot_node = function(gene_name) {
  probe_id = e2s[which(e2s$symbol == gene_name), "probe_id"]
  tb_expr = tibble(
    expr = exprs(eset)[which(rownames(exprs(eset)) == probe_id),],
    time_to_delivery = pData(eset)$time_to_delivery,
```

```
    gestage_delivery = pData(eset)$gestage_delivery
  )
  fit_expr = lm(expr ~ gestage_delivery, tb_expr)
  fit_time = lm(time_to_delivery ~ gestage_delivery, tb_expr)
  tb_expr %<>% mutate(res_expr = residuals(fit_expr))
  tb_expr %<>% mutate(res_time_to_delivery = residuals(fit_time))
  ggplot(tb_expr, aes(res_time_to_delivery, res_expr)) +
    geom_smooth(method = lm, se = FALSE, color = "grey", size = 2) +
    geom_point(size = 2) +
    ggtitle(gene_name) +
    xlab("resid(lm(time_to_delivery ~ gestage_delivery)") +
    ylab("resid(lm(expr ~ gestage_delivery))")
}
plot_node("IFNL1")
```

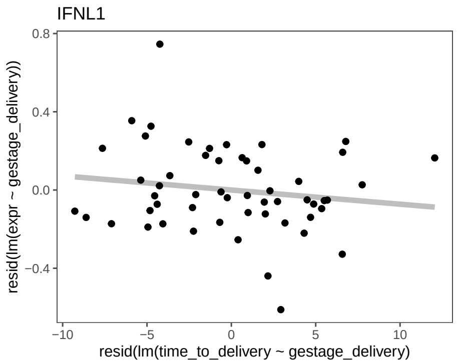


```
plot_node("SNORD18A")
```

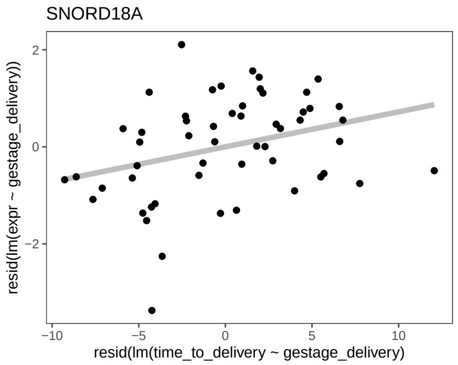

8 Pathways Analysis

Calculate number of genes that our network on known pathways have in common. Only keep pathways that overlap at least 5% of genes. Visualize pathway overlap.

```
plot_pathway = function(module) {
  if(length(V(module)) < 2)
    return("need two or more nodes")
```

```
    # get human pathways form kegg database
    pathways = keggList("pathway", "hsa")
    human_pathways = sub("path:", "", names(pathways))

    # kegg server only allow 10 request at the time
    n_request = ceiling(length(human_pathways)/10)
    chunk = function(x, n) split(x, sort(rank(x) %% n))
    chunks_pathways = chunk(1:length(human_pathways),n_request)

    # download from kegg server
    list_chunk_pathways = lapply(chunks_pathways,
                                 function(one_chunk_pathways) {
      cat("download chunk:",one_chunk_pathways,"\n")
      pathway_ids = human_pathways[one_chunk_pathways]
      setNames(keggGet(pathway_ids), pathway_ids)
      })

    # flatten list of lists
    all_pathways = unlist(list_chunk_pathways, recursive=FALSE)

    # check for all human pathways
    module_df = ggnetwork(module)
    ids = sapply(module_df$vertex.names,
                 function(id) which(id == mapped$STRING_id)[1])
    tb_pways = lapply(all_pathways, function(pway) {
      gene_desc = pway$GENE[c(F,T)]
      if(length(gene_desc) > 0) {
        gene_symbol = sapply(strsplit(gene_desc, split = ";"),
                             function(desc) desc[1])
        overlap = mean(gene_symbol %in% unique(mapped$symbol[ids]))
      } else {
        overlap = 0
      }
      tibble(
        name = pway$NAME,
        overlap = overlap
        )
    }) %>% bind_rows
    tb_pways %<>%
      dplyr::filter(overlap > 0.04) %>%
      dplyr::arrange(desc(overlap))
    tb_pways$name %<>% str_replace(" - Homo sapiens \\(human\\)","")
    tb_pways$name %<>% factor(levels = rev(tb_pways$name))
    ggplot(tb_pways, aes(x = 100*overlap,y = name)) +
      geom_point(size = 2) +
      theme(legend.position="none") +
      xlab("Overlap in %") +
      ylab("Pathway Name")
  }
  plot_pathway(module)
  ## [1] "need two or more nodes"
```

9 Time to Delivery Prediction

Use Lasso to predict time to delivery from gene expressions.

```
set.seed(0xdada)
exprs_table = t(exprs(eset))
sample_table = eset@phenoData@data
glmnet_cv = cv.glmnet(x = exprs_table,
                      y = sample_table$time_to_delivery)
plot(glmnet_cv)
```

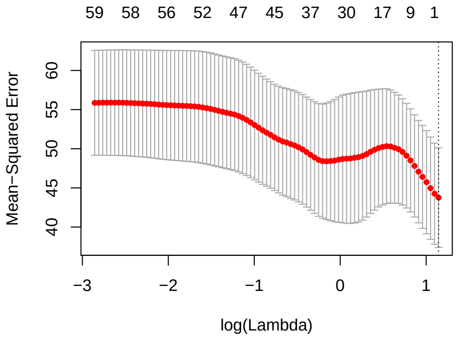


```
glmnet_fit = glmnet(x = exprs_table,
                    y = sample_table$time_to_delivery,
                    lambda = glmnet_cv$lambda.min)
nonzero = which(coef(glmnet_fit) > 0)
prob_ids_nonzero = rownames(coef(glmnet_fit))[nonzero]
e2s[which(e2s$probe_id %in% prob_ids_nonzero),]
## [1] probe_id symbol
## <0 rows> (or 0-length row.names)
```

Session Info

```
sessionInfo()
## R version 3.5.1 (2018-07-02)
## Platform: x86_64-apple-darwin15.6.0 (64-bit)
## Running under: macOS  10.15.3
##
## Matrix products: default
## BLAS: /Library/Frameworks/R.framework/Versions/3.5/Resources/lib/libRblas.0.dylib
## LAPACK: /Library/Frameworks/R.framework/Versions/3.5/Resources/lib/libRlapack.dylib
##
## locale:
## [1] en_US.UTF-8/en_US.UTF-8/en_US.UTF-8/C/en_US.UTF-8/en_US.UTF-8
##
## attached base packages:
## [1] stats4    parallel  stats     graphics  grDevices utils     datasets
## [8] methods   base
##
```

```
## other attached packages:
##  [1] pd.hta.2.0_3.12.2                DBI_1.0.0
##  [3] RSQLite_2.1.1                    readr_1.3.1
##  [5] sna_2.4                          network_1.15
##  [7] statnet.common_4.2.0             ggthemes_4.1.1
##  [9] ggnetwork_0.5.1                  intergraph_2.0-2
## [11] igraph_1.2.4.1                   KEGGREST_1.22.0
## [13] DLBCL_1.22.0                     BioNet_1.42.0
## [15] RBGL_1.58.2                      graph_1.60.0
## [17] STRINGdb_1.22.0                  dplyr_0.8.3
## [19] tibble_2.1.3                     stringr_1.4.0
## [21] statmod_1.4.32                   magrittr_1.5
## [23] ggfortify_0.4.7                  ggplot2_3.2.1
## [25] hta20transcriptcluster.db_8.7.0 org.Hs.eg.db_3.7.0
## [27] AnnotationDbi_1.44.0             glmnet_2.0-18
## [29] foreach_1.4.4                    Matrix_1.2-17
## [31] genefilter_1.64.0                affycoretools_1.54.0
## [33] limma_3.38.3                     oligo_1.46.0
## [35] Biostrings_2.50.2                XVector_0.22.0
## [37] IRanges_2.16.0                   S4Vectors_0.20.1
## [39] Biobase_2.42.0                   oligoClasses_1.44.0
## [41] BiocGenerics_0.28.0              BiocStyle_2.10.0
##
## loaded via a namespace (and not attached):
##   [1] utf8_1.1.4                  proto_1.0.0
##   [3] R.utils_2.8.0               tidyselect_0.2.5
##   [5] htmlwidgets_1.3             grid_3.5.1
##   [7] BiocParallel_1.16.6         munsell_0.5.0
##   [9] codetools_0.2-16            preprocessCore_1.44.0
##  [11] chron_2.3-53                withr_2.1.2
##  [13] colorspace_1.4-1            Category_2.48.1
##  [15] OrganismDbi_1.24.0          knitr_1.22
##  [17] rstudioapi_0.10             labeling_0.3
##  [19] GenomeInfoDbData_1.2.0      hwriter_1.3.2
##  [21] farver_2.0.1                bit64_0.9-7
##  [23] coda_0.19-3                 vctrs_0.2.1
##  [25] xfun_0.6                    biovizBase_1.30.1
##  [27] affxparser_1.54.0           R6_2.4.1
##  [29] GenomeInfoDb_1.18.2         locfit_1.5-9.1
##  [31] AnnotationFilter_1.6.0      bitops_1.0-6
##  [33] reshape_0.8.8               DelayedArray_0.8.0
##  [35] assertthat_0.2.1            scales_1.1.0
##  [37] nnet_7.3-12                 gtable_0.3.0
##  [39] affy_1.60.0                 ggbio_1.30.0
##  [41] ensembldb_2.6.8             rlang_0.4.2
##  [43] zeallot_0.1.0               splines_3.5.1
##  [45] rtracklayer_1.42.2          lazyeval_0.2.2
##  [47] acepack_1.4.1               dichromat_2.0-0
##  [49] checkmate_1.9.4             BiocManager_1.30.4
##  [51] yaml_2.2.0                  reshape2_1.4.3
##  [53] GenomicFeatures_1.34.8      backports_1.1.5
```

```
##  [55] Hmisc_4.2-0                  tools_3.5.1
##  [57] bookdown_0.9                 affyio_1.52.0
##  [59] gplots_3.0.1.1               ff_2.2-14
##  [61] RColorBrewer_1.1-2           gsubfn_0.7
##  [63] Rcpp_1.0.3                   hash_2.2.6.1
##  [65] plyr_1.8.5                   base64enc_0.1-3
##  [67] progress_1.2.2               zlibbioc_1.28.0
##  [69] purrr_0.3.3                  RCurl_1.95-4.12
##  [71] prettyunits_1.1.0            rpart_4.1-15
##  [73] sqldf_0.4-11                 ggrepel_0.8.1
##  [75] SummarizedExperiment_1.12.0 cluster_2.0.9
##  [77] data.table_1.12.6            ProtGenerics_1.14.0
##  [79] matrixStats_0.55.0           hms_0.5.2
##  [81] evaluate_0.13                xtable_1.8-4
##  [83] XML_3.98-1.19                gcrma_2.54.0
##  [85] gridExtra_2.3                compiler_3.5.1
##  [87] biomaRt_2.38.0               KernSmooth_2.23-15
##  [89] crayon_1.3.4                 ReportingTools_2.22.1
##  [91] R.oo_1.22.0                  htmltools_0.3.6
##  [93] GOstats_2.48.0               Formula_1.2-3
##  [95] tidyr_1.0.0                  geneplotter_1.60.0
##  [97] cli_2.0.1                    R.methodsS3_1.7.1
##  [99] gdata_2.18.0                 GenomicRanges_1.34.0
## [101] pkgconfig_2.0.3              GenomicAlignments_1.18.1
## [103] foreign_0.8-71               annotate_1.60.1
## [105] AnnotationForge_1.24.0       VariantAnnotation_1.28.13
## [107] digest_0.6.23                rmarkdown_1.12
## [109] htmlTable_1.13.2             edgeR_3.24.3
## [111] GSEABase_1.44.0              curl_4.3
## [113] Rsamtools_1.34.1             gtools_3.8.1
## [115] lifecycle_0.1.0              PFAM.db_3.7.0
## [117] fansi_0.4.1                  BSgenome_1.50.0
## [119] pillar_1.4.3                 lattice_0.20-38
## [121] GGally_1.4.0                 httr_1.4.0
## [123] plotrix_3.7-5                survival_2.44-1.1
## [125] GO.db_3.7.0                  glue_1.3.1
## [127] png_0.1-7                    iterators_1.0.10
## [129] bit_1.1-14                   Rgraphviz_2.26.0
## [131] stringi_1.4.5                blob_1.1.1
## [133] DESeq2_1.22.2                latticeExtra_0.6-28
## [135] caTools_1.17.1.2             memoise_1.1.0
```
