# Supplementary material for: Influenza-Induced Interferon Lambda Response Is Associated With Longer Time to Delivery Among Pregnant Kenyan Women
Source: Front Immunol. 2020 Mar 17;11:452. doi: 10.3389/fimmu.2020.00452 (PMC7089959; doi:10.3389/fimmu.2020.00452)
Supplement: Supplementary file 2 [file Presentation_1.zip › TimeToDelivery_qPCR_Mock.pdf]

Time to Delivery Analysis on Follow-Up qPCR Data

Christof Seiler

3 February 2020

Contents

1 Goal 2
2 Prerequisites 2
3 Import Data 2
4 Fit Model 3
4.1 RGS13 3
4.2 IFNL 7
Session Info 11

1 Goal

Analyze data from qPCR experiments.

2 Prerequisites

Install necessary packages from bioconductor repository. Run this code only once to install packages.

```
pkgs_needed = c("ggplot2","magrittr","readr","dplyr",
                "readxl","stringr","tidyr","car",
                "interactions","ggthemes","cowplot",
                "MASS", "boot")
letsinstall = setdiff(pkgs_needed, installed.packages())
if (length(letsinstall) > 0) {
  source("http://bioconductor.org/biocLite.R")
  biocLite(letsinstall)
}
```

Load packages.

```
library("magrittr")
library("ggplot2")
library("readr")
library("dplyr")
library("readxl")
library("stringr")
library("tidyr")
library("MASS")
library("interactions")
library("ggthemes")
library("cowplot")
library("car")
library("boot")
theme_set(theme_few())
scale_colour_discrete = function(...) scale_colour_few()
if(params$treatment == "H1N1") {
  color_condition = "#FAA43A"
} else {
  color_condition = "#5DA5DA"
}
params$treatment
## [1] "mock"
```

3 Import Data

Read, merge, and tidy sample tables.

```
sample_info_filename = "MSS Case_Control Matches (Masked)_new_matched.xlsx"
data_filename = "PTB Raw qPCR data.xlsx"
```

```
new_matched = read_excel(sample_info_filename)
new_matched %<>% mutate(time_to_delivery_calc =
                          gestage_enroll - gestage_delivery)
qpcr_data = read_excel(data_filename)
qpcr_data$SampleID %<>% as.character
qpcr_data %<>% dplyr::left_join(new_matched,by = "SampleID",
                                suffix = c("", "2"))
qpcr_data %<>% dplyr::select(sample_id = SampleID,
                             ptb_case,
                             time_to_delivery = time_to_delivery_calc,
                             gestage_delivery,
                             RGS13_baseline,
                             IFNL_baseline,
                             IFNL_flu)
qpcr_data %<>% gather(RGS13_baseline, IFNL_baseline, IFNL_flu,
                      key = "key", value = "expr")
qpcr_data %<>% mutate(
  treatment = if_else(condition = str_detect(qpcr_data$key, "baseline"),
                      true = "mock",
                      false = "H1N1"))
qpcr_data %<>% mutate(
  gene_name = sapply(strsplit(qpcr_data$key,split = "_"),
                     function(str) str[[1]]))
qpcr_data %<>% dplyr::select(-key)
qpcr_data$treatment = factor(qpcr_data$treatment,levels = c("mock","H1N1"))
qpcr_data %<>% rename(ptb = ptb_case)
qpcr_data
## # A tibble: 165 x 7
##    sample_id ptb   time_to_delivery gestage_delivery    expr treatment
##    <chr>     <chr>            <dbl>            <dbl>   <dbl> <fct>
##  1 1.1       cont~            -6.57             37.6 0.160   mock
##  2 1.2       case             -5.57             21.3 0.0876  mock
##  3 1.3       cont~           -27.4              42.4 0.00572 mock
##  4 2.1       case            -18.3              33.6 0.0540  mock
##  5 2.2       cont~           -21.9              37.4 0.00703 mock
##  6 2.3       cont~           -24.3              39.3 0.00144 mock
##  7 3.2       cont~           -19.4              38   0.00470 mock
##  8 3.3       case            -14.7              33.4 0.0167  mock
##  9 4.3       cont~           -22.1              40   0.0529  mock
## 10 5.1       cont~           -21.9              40.9 0.0104  mock
## # ... with 155 more rows, and 1 more variable: gene_name <chr>
```

4 Fit Model

4.1 RGS13

Subset to RGS13.

Time to Delivery Analysis on Follow-Up qPCR Data

```r
qpcr_data_RGS13 = qpcr_data %>% dplyr::filter(gene_name == "RGS13")
table(qpcr_data_RGS13$gene_name)
##
## RGS13
##    55
```

Plotting to see possible outliers.

```r
ggplot(qpcr_data_RGS13,aes(expr)) +
  geom_histogram(bins = 30) +
  facet_wrap(~ptb) +
  ggtitle("RGS13")
```

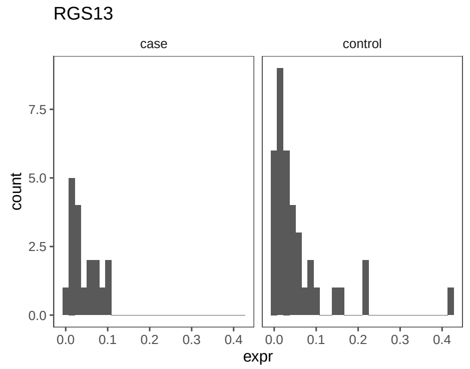


```r
ggplot(qpcr_data_RGS13,aes(x = time_to_delivery, y = expr,
                           color = treatment)) +
  geom_smooth(method = lm, se = FALSE) +
  geom_point() +
  geom_text(aes(label = ifelse(expr > quantile(expr, probs = 0.95),
                               sample_id,"")), hjust = 0, vjust = 0) +
  ggtitle("RGS13")
```

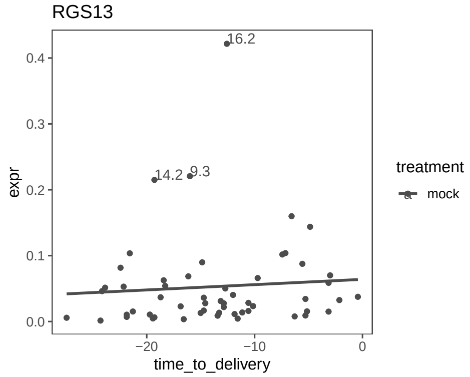


```r
ggplot(qpcr_data_RGS13,aes(x = gestage_delivery, y = expr,
                           color = treatment)) +
  geom_smooth(method = lm, se = FALSE) +
  geom_point() +
  geom_text(aes(label = ifelse(expr > quantile(expr, probs = 0.95),
                               sample_id,"")), hjust = 0, vjust = 0) +
```

```r
    ggtitle("RGS13")
```

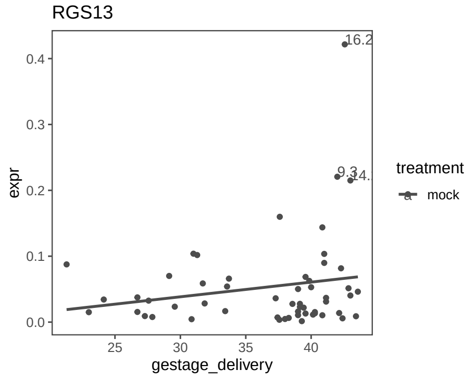

Fit model for `RGS13`.

```r
lm_fit = lm(expr ~ time_to_delivery + gestage_delivery, qpcr_data_RGS13)
summary(lm_fit)
##
## Call:
## lm(formula = expr ~ time_to_delivery + gestage_delivery, data = qpcr_data_RGS13)
##
## Residuals:
##      Min       1Q   Median       3Q      Max
## -0.08642 -0.02985 -0.01733  0.01426  0.32746
##
## Coefficients:
##                   Estimate Std. Error t value Pr(>|t|)
## (Intercept)      -0.104550   0.064954  -1.610  0.11354
## time_to_delivery  0.004714   0.001974   2.388  0.02061 *
## gestage_delivery  0.006059   0.002214   2.736  0.00848 **
## ---
## Signif. codes:  0 '***' 0.001 '**' 0.01 '*' 0.05 '.' 0.1 ' ' 1
##
## Residual standard error: 0.06689 on 52 degrees of freedom
## Multiple R-squared:  0.131,  Adjusted R-squared:  0.09754
## F-statistic: 3.918 on 2 and 52 DF,  p-value: 0.026
```

Fit robust model using rank-based estimates of regression coefficients.

```r
lm_fit = rlm(expr ~ time_to_delivery + gestage_delivery, qpcr_data_RGS13)
summary(lm_fit)
##
## Call: rlm(formula = expr ~ time_to_delivery + gestage_delivery, data = qpcr_data_RGS13)
## Residuals:
##      Min       1Q   Median       3Q      Max
## -0.04962 -0.02148 -0.01265  0.02540  0.36320
##
## Coefficients:
##                  Value   Std. Error t value
## (Intercept)      -0.0210  0.0383    -0.5479
```

```
## time_to_delivery  0.0025  0.0012     2.1358
## gestage_delivery  0.0026  0.0013     1.9913
##
## Residual standard error: 0.03594 on 52 degrees of freedom
```

Remove potential outlier and refit model to see if we still obtain a similar result.

```
qpcr_data_RGS13 %<>% dplyr::filter(sample_id != "16.2")
lm_fit = lm(expr ~ time_to_delivery + gestage_delivery, qpcr_data_RGS13)
summary(lm_fit)
##
## Call:
## lm(formula = expr ~ time_to_delivery + gestage_delivery, data = qpcr_data_RGS13)
##
## Residuals:
##      Min       1Q   Median       3Q      Max
## -0.06230 -0.02892 -0.01508  0.01854  0.16243
##
## Coefficients:
##                   Estimate Std. Error t value Pr(>|t|)
## (Intercept)      -0.041939   0.047497  -0.883   0.3814
## time_to_delivery  0.002920   0.001441   2.026   0.0480 *
## gestage_delivery  0.003507   0.001631   2.150   0.0363 *
## ---
## Signif. codes:  0 '***' 0.001 '**' 0.01 '*' 0.05 '.' 0.1 ' ' 1
##
## Residual standard error: 0.04805 on 51 degrees of freedom
## Multiple R-squared:  0.09024,    Adjusted R-squared:  0.05456
## F-statistic: 2.529 on 2 and 51 DF,  p-value: 0.08966
```

Plot of fitted intercept and slope with `gestage_delivery` fixed at its mean value.

```
coefs = coef(lm_fit)
gmine = ggplot(qpcr_data_RGS13,
               aes(x = time_to_delivery, y = expr,
                   shape = ptb, color = treatment)) +
  geom_point(size = 2) +
  scale_colour_manual(values = color_condition)

mean_gestage = mean(qpcr_data_RGS13$gestage_delivery)

gmine + geom_abline(intercept = coefs["(Intercept)"] +
                      mean_gestage*coefs["gestage_delivery"],
                    slope = coefs["time_to_delivery"],
                    color = color_condition, size = 1) + ggtitle("RGS13")
```

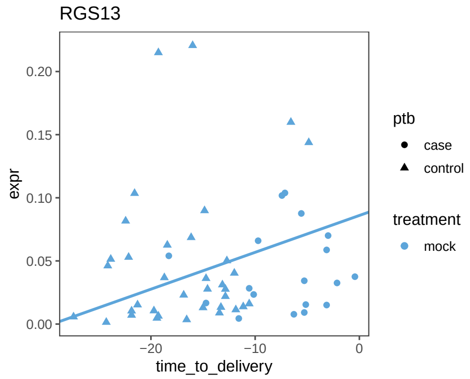

4.2 IFNL

Subset to `IFNL`.

```
qpcr_data_IFNL = qpcr_data %>%
  dplyr::filter(gene_name == "IFNL") %>%
  dplyr::filter(treatment == params$treatment)
```

Plotting to see possible outliers.

```
ggplot(qpcr_data_IFNL,aes(expr)) +
  geom_histogram(bins = 30) +
  facet_wrap(~ptb) +
  ggtitle("IFNL")
```

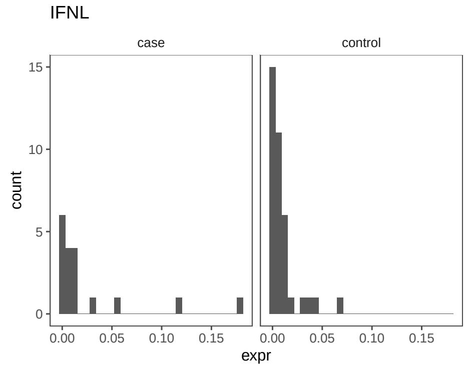


```
ggplot(qpcr_data_IFNL,aes(x = time_to_delivery, y = expr,
                          color = treatment)) +
  geom_smooth(method = lm, se = FALSE) +
  geom_point() +
  geom_text(aes(label = ifelse(expr > quantile(expr, probs = 0.95),
                               sample_id,"")), hjust = 0, vjust = 0) +
  ggtitle("IFNL")
```

Time to Delivery Analysis on Follow-Up qPCR Data

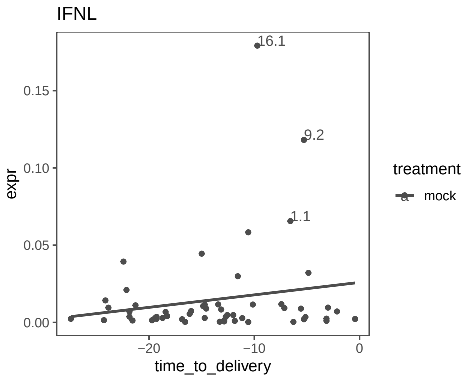


```r
ggplot(qpcr_data_IFNL,aes(x = gestage_delivery, y = expr,
                          color = treatment)) +
  geom_smooth(method = lm, se = FALSE) +
  geom_point() +
  geom_text(aes(label = ifelse(expr > quantile(expr, probs = 0.95),
                               sample_id,"")), hjust = 0, vjust = 0) +
  ggtitle("IFNL")
```

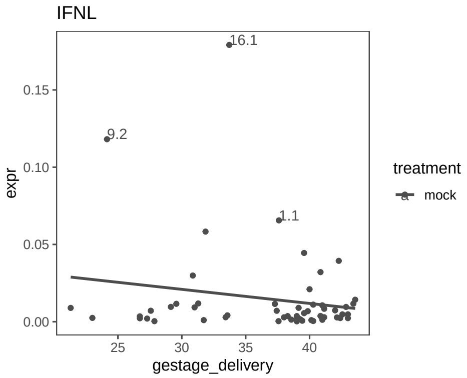

Fit model for `IFNL`.

```r
lm_fit = lm(expr ~ time_to_delivery + gestage_delivery, data = qpcr_data_IFNL)
summary(lm_fit)
##
## Call:
## lm(formula = expr ~ time_to_delivery + gestage_delivery, data = qpcr_data_IFNL)
##
## Residuals:
##       Min        1Q    Median        3Q       Max
## -0.024599 -0.012740 -0.007452 -0.001961  0.160878
##
## Coefficients:
##                    Estimate Std. Error t value Pr(>|t|)
## (Intercept)       0.0408508  0.0293275   1.393    0.170
## time_to_delivery  0.0004611  0.0008913   0.517    0.607
## gestage_delivery -0.0005373  0.0009997  -0.537    0.593
##
## Residual standard error: 0.0302 on 52 degrees of freedom
```

```
## Multiple R-squared:  0.03729,    Adjusted R-squared:  0.0002618
## F-statistic: 1.007 on 2 and 52 DF,  p-value: 0.3723
```

Fit robust linear model fit and bootstrap resampling for confidence intervals and p-values.

```
lm_fit_robust = rlm(expr ~ time_to_delivery + gestage_delivery,
                    data = qpcr_data_IFNL, maxit = 200)
summary(lm_fit_robust)
##
## Call: rlm(formula = expr ~ time_to_delivery + gestage_delivery, data = qpcr_data_IFNL,
##     maxit = 200)
## Residuals:
##       Min        1Q    Median        3Q       Max
## -0.006972 -0.004754 -0.002150  0.004525  0.172038
##
## Coefficients:
##                  Value   Std. Error t value
## (Intercept)       0.0078  0.0066     1.1872
## time_to_delivery  0.0001  0.0002     0.2646
## gestage_delivery  0.0000  0.0002    -0.0254
##
## Residual standard error: 0.00682 on 52 degrees of freedom
# bootstrap confidence interval and p-value
set.seed(0xdada)
lm_fit_robust_boot = Boot(lm_fit_robust, R = 1999)
summary(lm_fit_robust_boot)
##
## Number of bootstrap replications R = 1999
##                     original    bootBias     bootSE     bootMed
## (Intercept)       7.8003e-03  9.6065e-04 0.01075449  7.7908e-03
## time_to_delivery  5.2830e-05  2.6155e-05 0.00028394  5.9898e-05
## gestage_delivery -5.6898e-06 -6.7112e-06 0.00033346 -6.2906e-06
hist(lm_fit_robust_boot, legend="separate")
```

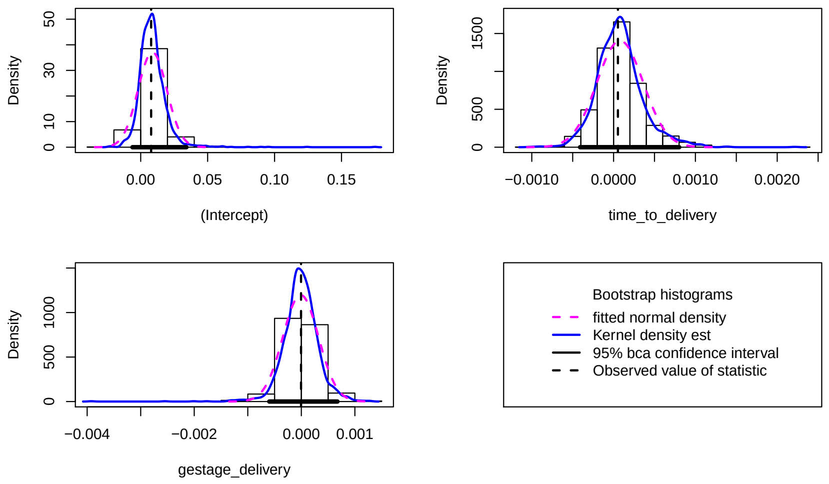


```
Confint(lm_fit_robust_boot, level = 0.95, type = "perc")
## Bootstrap percent confidence intervals
```

```
##
##                        Estimate         2.5 %       97.5 %
## (Intercept)       7.800291e-03 -0.0071847420 0.0296569633
## time_to_delivery  5.282996e-05 -0.0004260056 0.0007422489
## gestage_delivery -5.689807e-06 -0.0006310435 0.0006546222
Confint(lm_fit_robust_boot, level = 0.95, type = "bca")
## Bootstrap bca confidence intervals
##
##                        Estimate         2.5 %       97.5 %
## (Intercept)       7.800291e-03 -0.0061546661 0.0341378634
## time_to_delivery  5.282996e-05 -0.0004095204 0.0008017508
## gestage_delivery -5.689807e-06 -0.0005988178 0.0006742549
# p-value for stimulation x time to delivery interaction term
R = 1999
t_obsv = lm_fit_robust_boot$t0["time_to_delivery"]
t_boot = lm_fit_robust_boot$t[,"time_to_delivery"]-t_obsv
pvalue = (1 + sum(abs(t_boot) > abs(t_obsv))) / (R + 1)
pvalue
## [1] 0.816
```

Refit linear model after removing possible outlier.

```
qpcr_data_IFNL_with_outlier = qpcr_data_IFNL
lm_fit = lm(expr ~ time_to_delivery + gestage_delivery,
            data = qpcr_data_IFNL %>%
              dplyr::filter(sample_id != "1.1"))
summary(lm_fit)
##
## Call:
## lm(formula = expr ~ time_to_delivery + gestage_delivery, data = qpcr_data_IFNL %>%
##     dplyr::filter(sample_id != "1.1"))
##
## Residuals:
##       Min        1Q    Median        3Q       Max
## -0.024237 -0.011042 -0.007063 -0.001783  0.162282
##
## Coefficients:
##                    Estimate Std. Error t value Pr(>|t|)
## (Intercept)       0.0465914  0.0290324   1.605    0.115
## time_to_delivery  0.0001085  0.0009011   0.120    0.905
## gestage_delivery -0.0008509  0.0010004  -0.851    0.399
##
## Residual standard error: 0.02969 on 51 degrees of freedom
## Multiple R-squared:  0.0365, Adjusted R-squared:  -0.001281
## F-statistic: 0.9661 on 2 and 51 DF,  p-value: 0.3874
```

Residual plots.

```
tb_subset = qpcr_data_IFNL
rlm_delivery = rlm(expr ~ gestage_delivery, data = tb_subset)
tb_subset %<>% mutate(residual_expr = rlm_delivery$residuals)
```

```
rlm_delivery = rlm(residual_expr ~ time_to_delivery, data = tb_subset)
a = rlm_delivery$coefficients[[1]]
b = rlm_delivery$coefficients[[2]]
tb_subset %<>% mutate(y_hat = a + b*tb_subset$time_to_delivery)
gmine_with_outlier = ggplot(tb_subset, aes(time_to_delivery, residual_expr,
                                           color = ptb)) +
  geom_abline(intercept = a, slope = b, color = color_condition, size = 1) +
  geom_point() +
  ggtitle(paste0("IFNL - ", params$treatment)) +
  xlab("time to delivery") +
  ylab("residual expression") +
  theme(legend.position = "bottom")
gmine_with_outlier
```

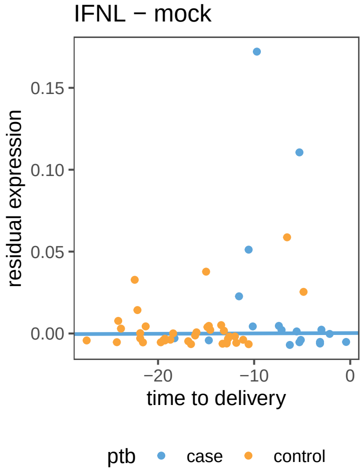


```
save(gmine_with_outlier,
     file = paste0("robust_fit_IFNL_", params$treatment,".Rdata"))
```

Session Info

```
sessionInfo()
## R version 3.5.1 (2018-07-02)
## Platform: x86_64-apple-darwin15.6.0 (64-bit)
## Running under: macOS  10.15.3
##
## Matrix products: default
## BLAS: /Library/Frameworks/R.framework/Versions/3.5/Resources/lib/libRblas.0.dylib
## LAPACK: /Library/Frameworks/R.framework/Versions/3.5/Resources/lib/libRlapack.dylib
##
## locale:
## [1] en_US.UTF-8/en_US.UTF-8/en_US.UTF-8/C/en_US.UTF-8/en_US.UTF-8
##
## attached base packages:
## [1] stats     graphics  grDevices utils     datasets  methods   base
```

```
##
## other attached packages:
##  [1] boot_1.3-22       car_3.0-3         carData_3.0-2
##  [4] cowplot_1.0.0     ggthemes_4.1.1    interactions_1.1.1
##  [7] MASS_7.3-51.4     tidyr_1.0.0       stringr_1.4.0
## [10] readxl_1.3.1      dplyr_0.8.3       readr_1.3.1
## [13] ggplot2_3.2.1     magrittr_1.5      BiocStyle_2.10.0
##
## loaded via a namespace (and not attached):
##  [1] tidyselect_0.2.5   xfun_0.6           purrr_0.3.3
##  [4] pander_0.6.3       haven_2.1.0        colorspace_1.4-1
##  [7] vctrs_0.2.1        generics_0.0.2     htmltools_0.3.6
## [10] yaml_2.2.0         utf8_1.1.4         rlang_0.4.2
## [13] pillar_1.4.3       foreign_0.8-71     glue_1.3.1
## [16] withr_2.1.2        jtools_2.0.1       lifecycle_0.1.0
## [19] munsell_0.5.0      gtable_0.3.0       cellranger_1.1.0
## [22] zip_2.0.2          evaluate_0.13      labeling_0.3
## [25] forcats_0.4.0      knitr_1.22         rio_0.5.16
## [28] curl_4.3           fansi_0.4.1        Rcpp_1.0.3
## [31] scales_1.1.0       backports_1.1.5    BiocManager_1.30.4
## [34] abind_1.4-5        farver_2.0.1       hms_0.5.2
## [37] digest_0.6.23      openxlsx_4.1.0.1   stringi_1.4.5
## [40] bookdown_0.9       grid_3.5.1         cli_2.0.1
## [43] tools_3.5.1        lazyeval_0.2.2     tibble_2.1.3
## [46] crayon_1.3.4       pkgconfig_2.0.3    zeallot_0.1.0
## [49] ellipsis_0.3.0     data.table_1.12.6  assertthat_0.2.1
## [52] rmarkdown_1.12     R6_2.4.1           compiler_3.5.1
```
